# Antimicrobial effects of repeated 405 nm low-level-laser photobiomodulation: an *In-vitro* study on surgical wound pathogens

**DOI:** 10.1007/s10103-026-04897-2

**Published:** 2026-05-27

**Authors:** Carola Kager, Andreas Heltschl, Manuel Holzer

**Affiliations:** 1 University of Applied Sciences Wiener Neustadt, Wiener Neustadt, Austria; 2 University of Applied Sciences Upper Austria, Linz, Austria; 3 Heltschl GmbH, Gallspach, Austria; 4 FH Gesundheitsberufe OÖ, Linz, Austria

**Keywords:** Photobiomodulation (PBM), Low-Level-Laser therapy (LLLT), Antimicrobial resistance (AMR), Wound infection

## Abstract

Antimicrobial resistance represents a major global health threat, requiring alternative non-antibiotic strategies. Light-based methods, particularly photobiomodulation (PBM) with 405 nm, have shown promise against clinically relevant pathogens. This in-vitro study investigated the effect of repeated PBM using a 405 nm low-level laser on the growth dynamics of wound infection-related bacteria. Five ATCC reference strains (*Staphylococcus aureus*, *Enterococcus faecalis*, *Escherichia coli*, *Methicillin-Resistant Staphylococcus aureus* [MRSA] and *Pseudomonas aeruginosa*) were cultured in tryptic soy broth and subjected to six irradiation cycles at 12-hour intervals (9 J/cm^2^ per cycle, 45 mW). Bacterial growth was monitored via optical density at 600 nm (OD_600_). Quantitative analysis included calculation of the area under the growth curve (AUC) and determination of threshold times for OD_600_ values between 0.1 and 0.5. Strain-specific effects were observed. *Escherichia coli* and MRSA exhibited significant AUC reductions compared to controls (*p* < 0.05); with MRSA responding after the first irradiation and *Escherichia coli* showing delayed effects from Stage_4 onwards. *Pseudomonas aeruginosa* showed a non-significant inhibitory trend, while *Enterococcus faecalis* and *Staphylococcus aureus* did not demonstrate significant differences. Repeated PBM with 405 nm selectively inhibited the growth of certain pathogens, underscoring its potential as a strain-specific, non-antibiotic strategy against wound infection-related bacteria. The results indicate a clear need for further research into mechanisms of action, standardized application protocols, and clinical transferability.

**Supplementary Information:**

The online version contains supplementary material available at 10.1007/s10103-026-04897-2.

## Introduction

Antimicrobial resistance (AMR) is one of the most pressing global health threats of the 21st century [1]. In 2019, AMR was linked to an estimated 4.95 million deaths worldwide. This number is likely underestimated due to incomplete surveillance systems and limited diagnostic infrastructure in many regions [2]. In response to this challenge, the World Health Organization (WHO) established the *Global Antimicrobial Resistance and Use Surveillance System* (GLASS). At the European level, the *European Centre for Disease Prevention and Control* (ECDC) systematically monitors healthcare-associated infections (HAIs) and AMR [3–5]. Together, these initiatives highlight the necessity of developing effective strategies to combat antimicrobial resistance worldwide.

Among HAIs, surgical-site infections (SSIs) are of particular concern, representing one of the most frequent postoperative complications. SSIs are associated with prolonged hospitalization, repeated surgical interventions, higher rates of admission to intensive care units, increased mortality, and significantly elevated healthcare costs [4–6]. Between 2018 and 2020, the ECDC documented more than one million surgical procedures across 13 EU/EEA countries, of which 19.680 were complicated by SSIs. The most frequently isolated pathogens included *Enterococcus spp*. (17.6%), *Escherichia coli* (17.2%), *Staphylococcus aureus* (15.2%), and *Pseudomonas aeruginosa* (4%) [4].

Although national surveillance systems suggest that resistance rates in Austria have stabilized [7], the global threat posed by multidrug-resistant organisms remains high [8]. With the development of new antibiotics slowing dramatically, non-antibiotic antimicrobial approaches have gained increasing attention [1, 2, 9]. Several alternative strategies are under investigation, including bacteriophage therapy, antimicrobial peptides, nanotechnology-based solutions, and light-based therapies such as photodynamic therapy (PDT) and photobiomodulation (PBM) [10, 11].

Classical PDT relies on the administration of an exogenous photosensitizer, which, when activated by visible light in the presence of oxygen, generates reactive oxygen species (ROS) capable of killing microbial cells [12–14]. Importantly, an increasing body of evidence indicates that many pathogenic microorganisms naturally contain endogenous photosensitizers – particularly porphyrins – that can be activated by violet-blue light without the need for additional compounds [15, 16].

Among light-based modalities, violet-blue light at 405 nm has received considerable attention for its antimicrobial potential. Its mechanism is mainly attributed to the excitation of endogenous porphyrins in bacterial cells, which then produce cytotoxic ROS that inactivate bacteria. Compared to ultraviolet radiation (up to 380 nm), 405 nm light provides antimicrobial activity without significant cytotoxicity to mammalian cells, making it a promising candidate for clinical use [15–17]. However, the limited tissue penetration depth of this wavelength [18] restricts its use to superficial or shallow infections, such as SSIs and other wound-related infections of major clinical importance [19].

Several studies have demonstrated that both Gram-positive and Gram-negative bacteria can be inactivated by 405 nm light under in-vitro conditions. Gram-positive species often appear more susceptible, possibly due to higher intracellular porphyrin concentrations and structural differences in the cell wall compared to Gram-negative bacteria. These species-specific differences highlight the complexity of light–microbe interactions [19–22].

The present in-vitro study investigated the effect of repeated PBM with a 405 nm low-level laser on the growth dynamics of wound infection-related bacteria. Five ATCC^®^ reference strains were selected for their clinical relevance in SSIs: *Enterococcus faecalis*, *Escherichia coli*, methicillin-resistant *Staphylococcus aureus* (MRSA), *Pseudomonas aeruginosa*, and *Staphylococcus aureus*. The experimental design included six irradiation cycles administered at 12-hour intervals. Bacterial growth was continuously monitored via optical density at 600 nm (OD_600_). Quantitative analysis was based on the area under the growth curve (AUC) and on threshold times for defined OD_600_ values.

This approach was chosen to address three central research questions:

- Does PBM with a 405 nm low-level laser affect the growth of selected bacteria, as measured by the AUC, compared to the non-irradiated control group?
- Does repeated irradiation, compared to a single irradiation, have a stronger impact on bacterial growth?
- Does irradiation lead to changes in the growth curve progression, as measured by the time points at which defined OD_600_ thresholds are reached, compared to the non-irradiated control group?

By systematically analyzing strain-specific responses to repeated PBM, this study aims to expand the evidence base for light-based antimicrobial strategies and contribute to the development of novel adjunctive approaches in the fight against AMR.

## Materials and methods

### Bacterial strains and culture conditions

The following ATCC reference strains were used: *Enterococcus faecalis* (ATCC^®^ 29212™), *Escherichia coli* (ATCC^®^ 25922™), MRSA (ATCC^®^ 33591™), *Pseudomonas aeruginosa* (ATCC^®^ 27853™), and *Staphylococcus aureus* (ATCC^®^ 29213™). Strains were obtained as lyophilized reference preparations (KWIK-STIK™, Microbiologics Inc.) and reactivated according to the manufacturer’s instructions. Initial cultivation was performed on Columbia agar with 5% sheep blood (COS) at 35 °C for 24 ± 1 *h* under aerobic conditions.

For experiments, single colonies were suspended in sterile saline to a 0.5 McFarland standard and transferred into tryptic soy broth (TSB) to prepare bacterial suspensions. TSB was chosen based on pre-tests confirming reproducible growth for all strains.

### Experimental design

**Fig. 1:**
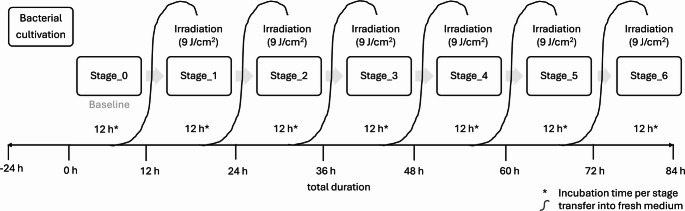
Schematic overview of the experimental setup and workflow. Bacterial cultures were grown on universal agar (Columbia blood agar, COS) for 24 h prior to the start of the experiment. At time zero (0 h), a bacterial suspension adjusted to a McFarland standard of 0.5 was prepared in tryptic soy broth (TSB). Cultures were incubated for 12 h, and bacterial growth was continuously monitored by OD_600_ measurements at 30-minute intervals using a microplate reader (Stage_0, baseline). After 12 h, cultures were transferred into fresh medium and, depending on the experimental group, exposed for the first time to an irradiation dose of 9 J/cm^2^, followed by further incubation and OD_600_ measurements (Stage_1). This procedure was repeated analogously up to Stage_6. In total, one experimental run comprised seven consecutive stages over a period of 84 h

The study was carried out over seven consecutive stages (Stage_0 – Stage_6), as schematically illustrated in Fig. 1. Each stage lasted 12 h, after which bacterial cultures were transferred into fresh medium. Where applicable, irradiation was applied at the start of the new stage. Stage_0 served as baseline to establish uniform starting conditions. From Stage_1 to Stage_6, repeated irradiation cycles were administered at 12-hour intervals.

Two experimental groups were defined:

- Control group (ctrl.): no irradiation.
- Irradiated group (irrad.): samples exposed to 405 nm low-level laser treatment.

At Stage_0, bacterial suspensions were inoculated into 12-well microtiter plates to establish uniform growth conditions. Each well contained *2700 µl* of TSB plus *300 µl* of bacterial suspension; blank wells were filled with *3000 µl* of TSB. Stage_0 was performed exclusively for cultivation under identical conditions for both groups, without irradiation. After 12 h of incubation at 35 °C, *30 µl* from each culture was transferred into fresh medium (*2970 µl* TSB + *30 µl* inoculum) to initiate Stage_1. From this point on, the procedure was repeated until Stage_6, with well positions kept constant throughout. The irradiated group was exposed to 405 nm low-level laser light at the beginning of each stage, whereas control wells were handled identically without irradiation.

### Irradiation setup and parameters

**Fig. 2:**
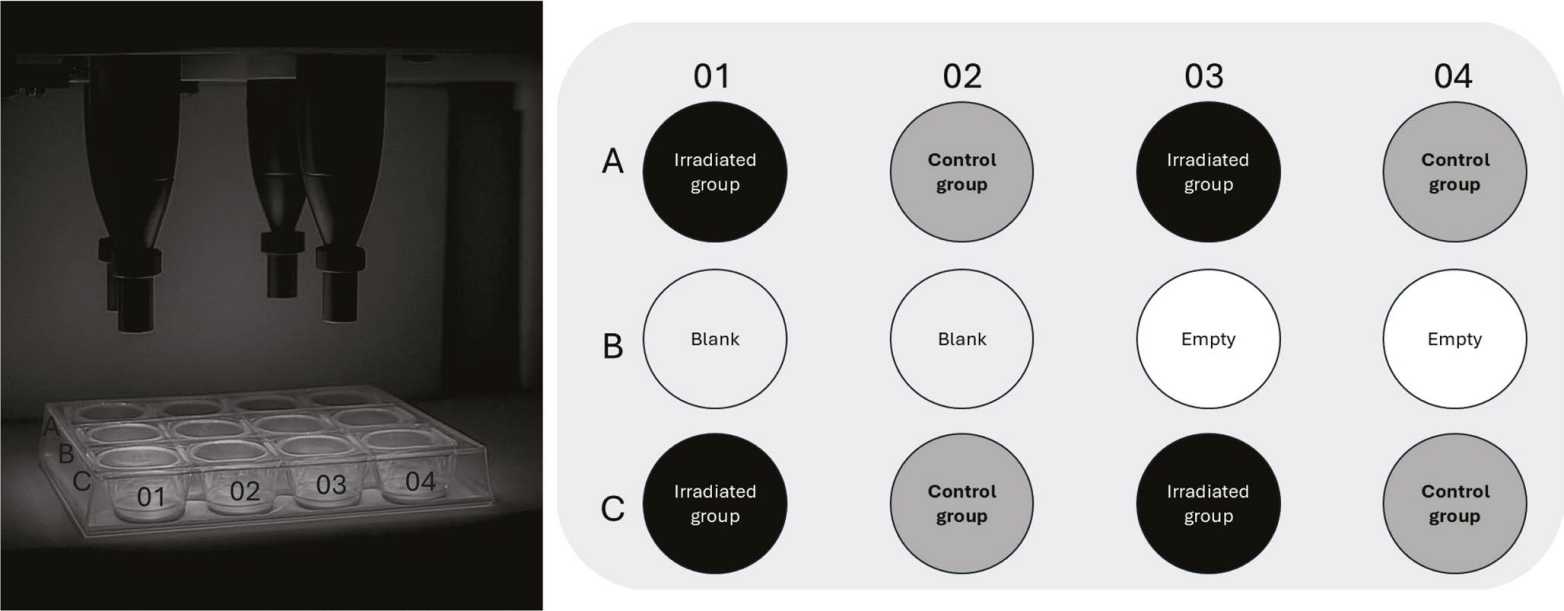
Irradiation setup and pipetting scheme of the 12-well microtiter plate. On the left is the irradiation unit with four vertically arranged laser emitters, which enable defined simultaneous irradiation of individual wells of the 12-well microtiter plate positioned below. On the right is the corresponding pipetting scheme. Black fields indicate the irradiated samples (Irradiated group), dark gray fields indicate the non-irradiated control samples (Control group). Light gray fields represent blank wells containing only tryptic soy broth (TSB), while white fields mark empty, unoccupied positions (Empty)

Irradiation was performed with a custom-built device (Heltschl GmbH), schematically illustrated in Fig. 2, equipped with four vertically aligned semiconductor laser diodes (λ = 404 ± 0.5 nm, continuous wave, 45 mW each). The system was designed to simultaneously irradiate four defined wells of the microtiter plate (A01, A03, C01, C03). Samples were irradiated directly in the microtiter plates without removal. Table 1 summarizes the applied irradiation parameters.

**Table 1:** Irradiation parameters

Irradiation parameters	
Wavelength	404 ± 0.5 nm
Average output power	45 mW
Spot size at target/ irradiated area	3.97 cm^2^
Irradiance at target	11.34 mW/cm^2^
Exposure time	795 s
Fluence	9 J/cm^2^
Radiant energy	35.73 J
Number of irradiation spots	four
Application technique	in vitro – liquid growth medium: TSB
Number and frequency of treatment sessions	six treatment sessions at 12-hour intervals
Total cumulative energy	214.4 J

### Growth measurement (OD_600_)

Growth was monitored spectrophotometrically using a SPECTROstar^®^ Nano (BMG Labtech). OD_600_ was measured every 30 min over a 12 h period, resulting in 25 measurements per stage. The incubation chamber was maintained at 35 °C. Prior to each measurement cycle, the microplate was shaken for 45 s at 100 rpm using a double-orbital movement. No continuous shaking or stirring was applied during incubation or irradiation periods.

OD_600_ values were used as relative measures of bacterial biomass. Conversion to colony-forming units (CFU) was not performed, as the analysis focused on relative growth kinetics.

### Data processing and statistical analyses

Raw OD_600_ data were exported and processed in MATLAB R2024b. Two main parameters were extracted:

- AUC: Calculated using the trapezoidal rule after normalization of OD_600_ values, representing cumulative bacterial growth over each 12-hour cycle.
- Threshold times: Defined as the time (h) required to reach OD_600_ values of 0.1, 0.2, 0.3, 0.4, and 0.5, determined by linear interpolation between adjacent measurements. These thresholds were selected to represent the exponential growth phase. In cases where a defined OD_600_ threshold was not reached within the measured range or within the exponential growth phase, no threshold time could be determined. Corresponding values are reported as “not reached” (n.r.) and are provided in Supplementary Data S2.

Statistical analyses were performed with IBM SPSS Statistics (version 30.0.0.0). Descriptive statistics included the median, 25th and 75th percentiles, and interquartile range (IQR). The Shapiro–Wilk test was applied to assess normality of the data distribution. Due to small sample sizes (*n* < 30) and deviations from normality in several datasets, non-parametric testing was applied. Group comparisons (control vs. irradiated) were performed using the Mann-Whitney U test. To correct multiple testing, the Bonferroni-Holm procedure was used. Corrected p-values (p_corr) < 0.05 were considered statistically significant.

This combined approach allowed evaluation of both overall growth (AUC) and temporal dynamics (threshold times), while ensuring statistical rigor in the detection of strain-specific effects.

All raw OD_600_ datasets underlying the analyses are provided in the Supplementary Material (Supplementary Data S1), while interpolated threshold time-point data are reported in Supplementary Table S2 to ensure transparency and reproducibility.

### Quality control

Each strain was tested in two independent experiments on separate days with eight replicates per group (control vs. irradiated). Only complete datasets free of contamination were included in the analysis. Purity was verified by streaking cultures on COS agar after each stage and inspecting growth after 24 ± 1 *h* incubation at 35 °C.

## Results

### Baseline conditions (Stage_0)

To ensure comparability, baseline conditions were analyzed at Stage_0 prior to the first irradiation. Median AUC values across all five bacterial strains showed comparable ranges between control and irradiated groups, with no significant differences detected (*p*_corr ≥ 0.05, Mann–Whitney U test). This confirmed standardized starting conditions.

### Methicillin-Resistant Staphylococcus Aureus (MRSA)

Distinct inhibitory effects were observed for MRSA. Already at Stage_1, irradiated samples showed lower median AUC values than controls (irrad.: 4.16 vs. ctrl.: 4.46 OD_600_ × h). This difference persisted up to and including Stage_5. Mann–Whitney U tests revealed consistently significant differences from Stage_1 through Stage_5 (*p*_corr < 0.01). At Stage_6, increased variability prevented the detection of a significant difference (*p*_corr = 0.390). While earlier stages showed narrow interquartile ranges, both groups exhibited greater dispersion in Stage_6, suggesting variability in late-cycle responses.

Threshold analyses confirmed these findings. Already in Stage_1, irradiated samples exhibited a significant delay (OD_600_ = 0.1: irrad.: 3.16 h vs. ctrl.: 2.80 h). This pattern continued across most stages and thresholds. Only in Stage_6 did the values between the two groups converge again.

**Fig. 3:**
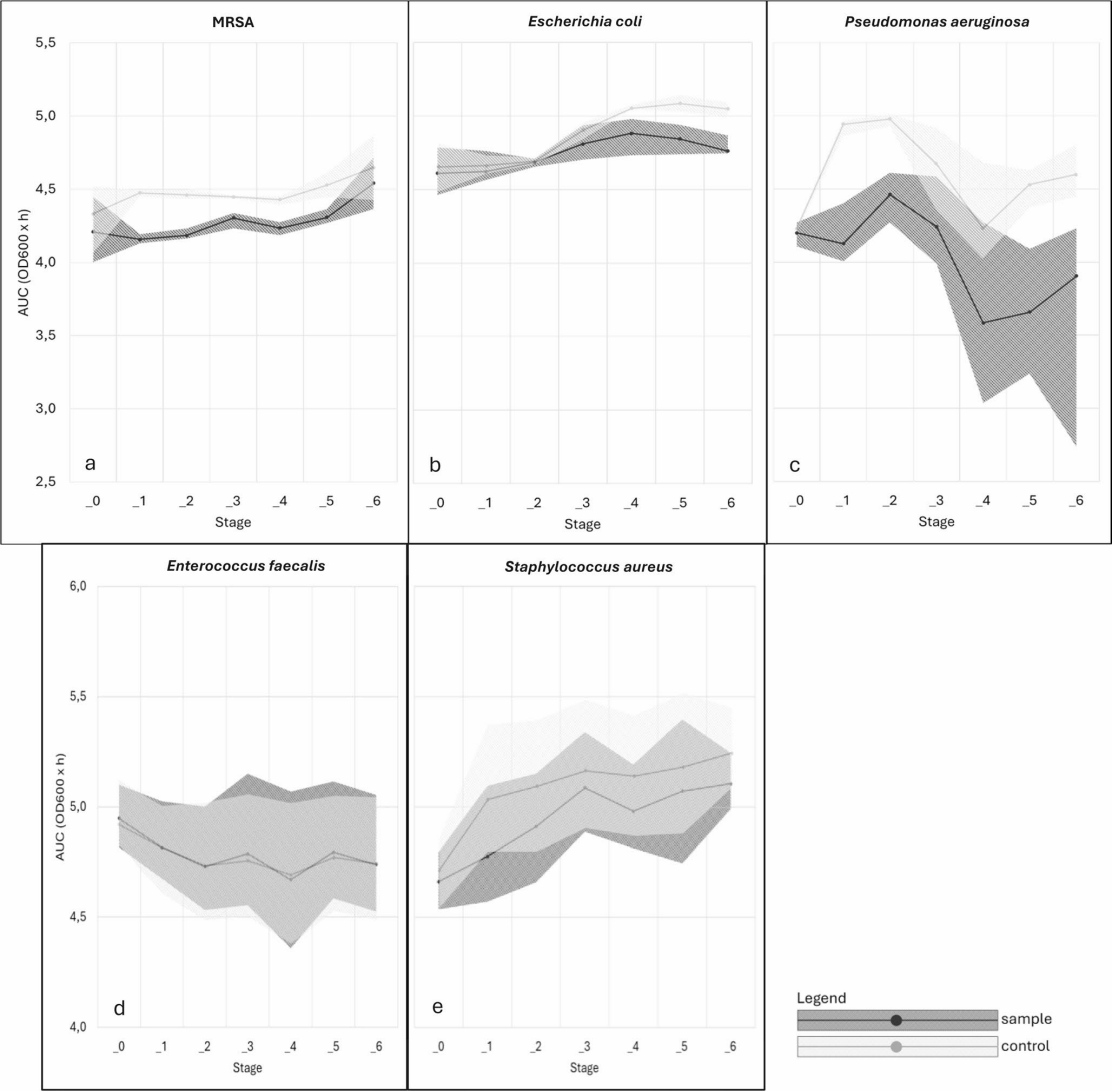
Median course of the AUC (OD600 x h) with 25th-75th percentile band. The figure shows the median area under the growth curve (AUC–OD_600_ × h) over six irradiation cycles (Stage_0 – Stage_6) for (**a**) methicillin-resistant Staphylococcus aureus (MRSA), (**b**) Escherichia coli, (**c**) Pseudomonas aeruginosa, (**d**) Enterococcus faecalis, and (**e**) Staphylococcus aureus. The figures show the median values of the irradiated samples (sample) and the untreated controls (control); the shaded areas correspond to the respective interquartile ranges (IQR). Stage_0 represents the initial conditions before the first irradiation. The AUC was calculated from OD_600_ time series and serves as a measure of cumulative growth performance within an irradiation cycle. Statistical evaluations were performed using the Mann–Whitney U test with Bonferroni–Holm correction (p_corr), but are not directly annotated in the figure

Thus, MRSA demonstrated consistent and significant growth inhibition over five irradiation cycles, which was no longer detectable in Stage_6, as shown in Fig. 3a.

### Escherichia coli

A strain-specific effect was observed in *Escherichia coli*. During the first three cycles (Stage_1 – Stage_3), AUC values between control and irradiated groups remained comparable, with no significant differences (*p*_corr ≥ 0.05). Starting with Stage_4, a divergence emerged: median AUC values of irradiated samples were consistently lower than those of controls. This reduction persisted through Stage_5 and Stage_6. Mann–Whitney U tests confirmed significant differences in AUC from Stage_4 onwards (Stage_4: *p*_corr = 0.015; Stage_5: *p*_corr = 0.012; Stage_6: *p*_corr = 0.006).

Threshold analyses supported this finding. From Stage_4 onwards, irradiated cultures required longer times to reach OD_600_ values of 0.1–0.5 compared with controls. For example, at Stage_4, the OD_600_ = 0.3 threshold was reached at a median of 3.27 h in irradiated samples versus 3.14 h in controls. By Stage_6, delays extended across all thresholds, with statistical significance for nearly all comparisons (*p*_corr < 0.05).

These results indicate that repeated irradiation progressively reduced growth dynamics of *Escherichia coli*, with significant effects becoming evident only after multiple treatment cycles, as illustrated in Fig. 3b.

### Pseudomonas aeruginosa

For *Pseudomonas aeruginosa*, irradiated cultures consistently exhibited lower AUC values than controls; however, these differences did not reach statistical significance (*p*_corr ≥ 0.05 across all stages). Median reductions in AUC were modest and accompanied by overlapping interquartile ranges.

Threshold analyses showed delayed attainment of OD_600_ values in irradiated samples, particularly at lower thresholds (0.1–0.2). Yet, these delays were minor in magnitude (0.05–0.15 h) and varied inconsistently across stages. After correction for multiple testing, no reproducible statistical significance was observed.

Overall, although a non-significant inhibitory trend was apparent, repeated irradiation did not yield robust growth-inhibitory effects on *Pseudomonas aeruginosa* under the tested conditions (Fig. 3c).

### Enterococcus faecalis

Across all irradiation cycles (Stage_1 – Stage_6), no consistent inhibitory effect was observed in the irradiated group compared to controls. Median AUC values remained similar between groups, with overlapping interquartile ranges. Statistical testing revealed no significant differences at any stage (*p*_corr ≥ 0.05).

Threshold analyses demonstrated that irradiated cultures occasionally reached OD_600_ thresholds slightly earlier than controls. However, these differences were inconsistent across stages and did not persist at higher OD_600_ levels (≥ 0.4). No reproducible trend was evident, and statistical testing yielded significance only in isolated cases that did not withstand Bonferroni–Holm correction.

Overall, no significant effect of 405 nm PBM on the growth of *Enterococcus faecalis* could be demonstrated as indicated by the largely overlapping AUC distributions in Fig. 3d.

### Staphylococcus aureus

The growth dynamics of *Staphylococcus aureus* were largely unaffected by irradiation. Median AUC values remained comparable between irradiated and control groups across all stages, with overlapping interquartile ranges. No statistically significant differences were observed (*p*_corr ≥ 0.05).

Threshold analyses indicated minimal variation between groups, with occasional earlier threshold attainment in irradiated samples. However, these differences were inconsistent and not statistically significant.

Overall, repeated 405 nm PBM did not exert a significant effect on the growth dynamics of *Staphylococcus aureus*, as shown in Fig. 3e.

### Summary of finding

The photobiological response to repeated 405 nm PBM varied across species. Significant growth inhibition was observed in MRSA (Stage_1 – Stage_5) and *Escherichia coli* (Stage_4 – Stage_6). *Pseudomonas aeruginosa* showed a non-significant inhibitory trend, while *Enterococcus faecalis* and *Staphylococcus aureus* were unaffected. Thus, repeated PBM exerted selective antimicrobial activity, most pronounced against MRSA and *Escherichia coli*.

## Discussion

The present study examined the effect of repeated PBM with a 405 nm low-level laser on five bacterial species frequently implicated in SSIs. The results revealed strain-specific responses: significant inhibition was observed in MRSA and *Escherichia coli*. *Pseudomonas aeruginosa* displayed a non-significant inhibitory trend, while *Enterococcus faecalis* and *Staphylococcus aureus* showed no significant changes. These outcomes confirm and expand current evidence on the antimicrobial activity of 405 nm PBM.

### Strain-specific interpretation

MRSA exhibited the most immediate and robust inhibition, with significant reductions in AUC already after the first irradiation cycle and persisting through Stage_5. These findings are consistent with studies reporting high light sensitivity of MRSA. Bumah et al. (2013) observed significant inhibition already at 3 J/cm² and nearly complete inactivation at 60 J/cm² [23]. In a later study, Bumah et al. (2015) reported full suppression at 55–60 J/cm² for lower inocula, while higher bacterial loads required two applications totaling 220 J/cm² [24]. Enwemeka et al. (2008) demonstrated > 90% inactivation at 55 J/cm² [25], and Maclean et al. (2009) reported a 5-log10 reduction at 45 J/cm² [26]. All studies attributed the effect to ROS generation mediated by photoactivated porphyrins. The attenuation observed in Stage_6 of the present study may reflect adaptive stress responses such as increased antioxidant activity or cell wall modifications [27, 28].

*Escherichia coli* exhibited significant inhibition beginning at Stage_4, after a cumulative dose of 36 J/cm². Median AUC values decreased, and threshold analyses showed delayed OD_600_ attainment. A slight initial increase in AUC before Stage_4 may explain the absence of earlier effects. The results suggest a cumulative inhibitory action of repeated irradiation. Lipovsky et al. (2010) reported strong inhibition at 30 J/cm² using 415 nm [29]. Maclean et al. (2009) observed a 3-log10 reduction at 180 J/cm² [26], while Murdoch et al. (2012) achieved 5-log10 reduction at 288 J/cm² [30]. Gupta et al. (2015) also reported strong effects above 200 J/cm² [31]. Porphyrin-mediated sensitization appears relevant: Imtiaz et al. (2015) demonstrated endogenous protoporphyrin IX production in *Escherichia coli* [32], contrasting with *Enterococcus faecalis*. Resistance status seems not to reduce light sensitivity: Barneck et al. (2016), dos Anjos et al. (2019), and Halstead et al. (2016) showed that resistant strains were as sensitive—or more sensitive—than susceptible strains [16, 33, 34]. Rhodes et al. (2016) confirmed complete inactivation of ampicillin-resistant *Escherichia coli* at 67.5 J/cm² [35]. The present findings support a threshold-dependent photoinactivation mechanism.

*Pseudomonas aeruginosa* showed inconsistent inhibition: no clear effect in early stages, but significant differences reappeared at Stage_5 and Stage_6. Threshold analyses indicated progressive delays in OD_600_ attainment, suggesting cumulative growth suppression. However, variability increased and a consistent dose-response was lacking. Guffey & Wilborn (2006) reported > 90% reduction at doses ≤ 10 J/cm² [36], indicating high light sensitivity under their conditions. Amin et al. (2016) achieved 3-log10 reduction at 48 J/cm² using 415 nm, but no cumulative effect over repeated cycles of 36 J/cm² [37]. Barneck et al. (2016) demonstrated full inactivation at 127.8 J/cm², while low doses (~ 5 J/cm²) caused paradoxical stimulation [33]. Fila et al. (2016) found no effect at 10 J/cm², but bactericidal action above 50 J/cm² [38]. Detection of coproporphyrin III and inhibition of virulence factors such as pyocyanin confirm ROS-mediated photoinactivation [31, 37, 38]. The heterogeneity observed in the present study may reflect subpopulation differences within the ATCC strain.

*Enterococcus faecalis* showed no significant inhibitory effect on AUC or growth kinetics. Threshold analyses indicated slightly earlier OD_600_ attainment in irradiated samples, suggesting preserved or even accelerated growth. Thus, *Enterococcus faecalis* appeared phototolerant under the tested conditions. Maclean et al. (2009) reported *Enterococcus faecalis* as the least sensitive species, showing significant reduction only at 216 J/cm² [26]. Other studies confirmed minimal effects even with clinical isolates [15, 31, 39]. Frankenberg et al. (2002) demonstrated the absence of the porphyrin biosynthetic pathway [40], explaining the lack of chromophores required for ROS-mediated inactivation. Compared to other Gram-positives, *Enterococcus faecalis* consistently shows the lowest degree of photodynamic sensitivity. The present findings therefore align well with published data, confirming its high light tolerance.

*Staphylococcus aureus* showed no significant inhibition under the applied conditions. A trend toward lower AUC values and delayed OD_600_ attainment was noted, but differences remained inconsistent and statistically non-significant. These findings suggest that the applied dose was insufficient to induce robust effects. Maclean et al. (2009), Ferrer-Espada et al. (2018), and Barneck et al. (2016) reported effective inactivation only at higher doses (> 100 J/cm²) [26, 33, 39]. Barneck et al. (2016) demonstrated complete inactivation at 130 J/cm², whereas shorter exposures resulted in only 23.5% reduction [33]. Guffey & Wilborn (2006) found 83% reduction with a single irradiation, but diminished efficacy upon repetition [36]. Overall, the data suggest that *S. aureus* requires higher doses or different conditions to achieve consistent photoinactivation.

### Pooled analysis of growth dynamics

**Fig. 4:**
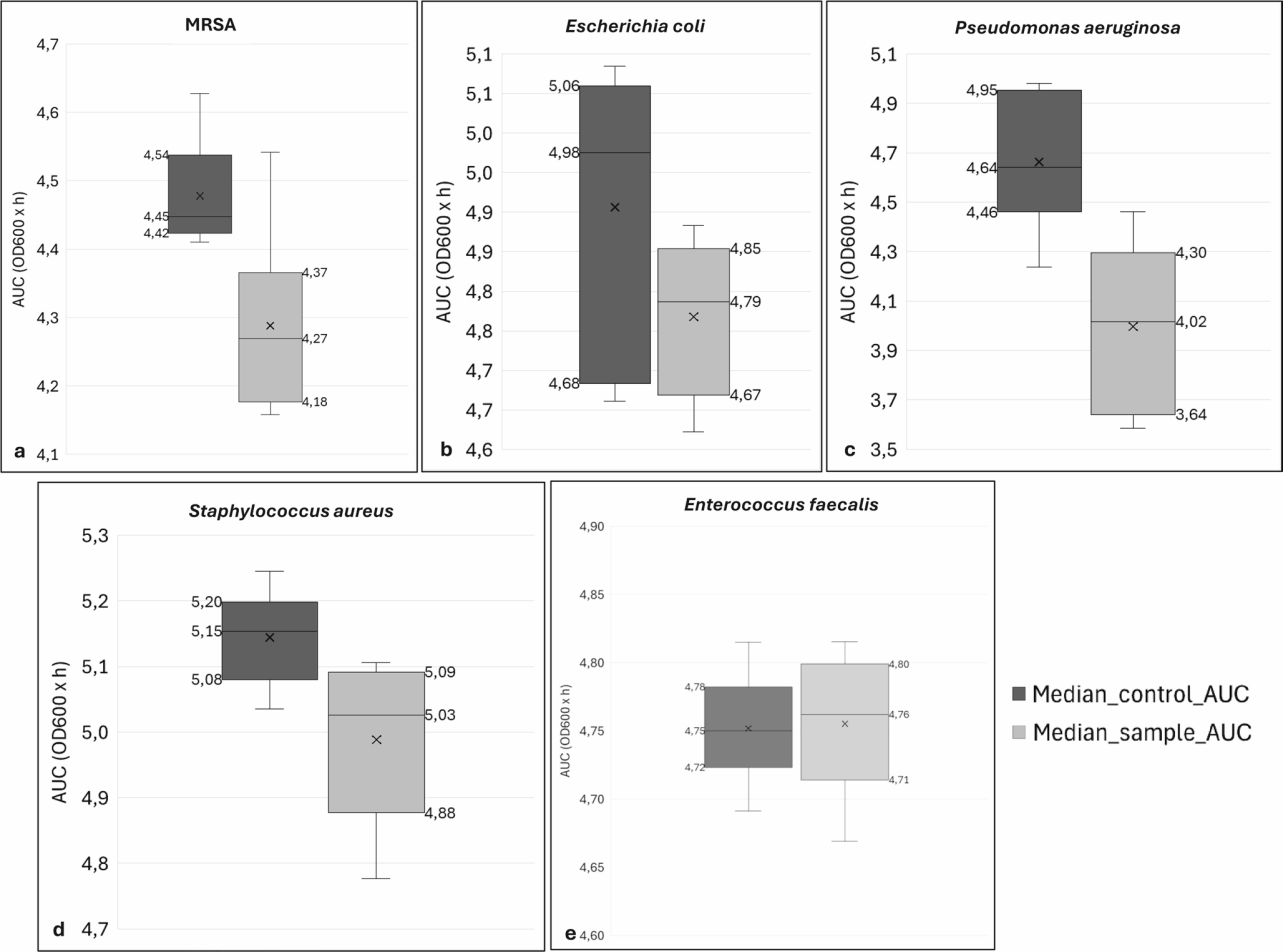
Pooled AUC distributions following repeated 405 nm photobiomodulation. Overview boxplots comparing median AUC values between control and irradiated samples, with AUCs pooled across all irradiation cycles (Stage_1 – Stage_6). Panels (a–e) correspond to methicillin-resistant Staphylococcus aureus (**a**), Escherichia coli (**b**), Pseudomonas aeruginosa (**c**), Staphylococcus aureus (**d**), and Enterococcus faecalis (**e**). The plots illustrate organism-specific differences in growth dynamics and the magnitude of photobiomodulatory effects following repeated 405 nm irradiation. Data are presented as medians with interquartile ranges, with boxes representing the 25th–75th percentiles and horizontal lines indicating median values

Figure 4 provides an integrative visualization of the growth-inhibiting effects of 405 nm PBM based on pooled AUC data. The clear separation of the interquartile ranges (IQR) observed for MRSA supports the conclusion of a consistent reduction in cumulative growth performance after irradiation (Fig. 4a). A comparable shift in the distribution toward lower AUC values is also evident for *Pseudomonas aeruginosa* (Fig. 4c), although this effect must be interpreted with caution given the marked variability observed across the different stages. No complete separation of the interquartile ranges is apparent for *Escherichia coli* (Fig. 4b), however, the clear difference in median AUC values between irradiated and control samples (irrad.: 4.79 vs. ctrl.: 4.98 OD₆₀₀ × h) is consistent with the delayed growth dynamics observed in the stage-specific analyses. The partial overlap of the distributions is more likely to reflect the higher variability within the control group than the absence of an irradiation effect. The weak but consistent trend previously observed for *Staphylococcus aureus* (Fig. 4d) is also reflected in the pooled presentation, while *Enterococcus faecalis* (Fig. 4e) remains unaffected, consistent with the stage-specific results.

### Mechanistic insights

The antimicrobial activity of 405 nm PBM is mainly driven by excitation of endogenous porphyrins, leading to ROS generation [26, 32, 39]. Gram-positive bacteria, such as MRSA, accumulate higher porphyrin levels and lack an outer membrane, which facilitates phototoxic damage [23, 26]. In contrast, the outer membrane of Gram-negative species like *Escherichia coli* and *Pseudomonas aeruginosa* limits photon penetration and ROS diffusion, with additional modulation by pigments such as pyocyanin [37, 38].

The loss of inhibition in MRSA at Stage_6 likely reflects phenotypic adaptation through stress response pathways [27, 28], although stable resistance to photodynamic inactivation has not been reported [24, 26].

### Clinical perspectives

The selective inhibition of MRSA and *Escherichia coli* underscores the clinical relevance of 405 nm PBM, particularly as both pathogens are major SSI contributors and often multidrug resistant [23, 26, 35]. Beyond its antimicrobial activity, PBM is non-invasive, exhibits minimal cytotoxicity, and supports wound healing [15, 41]. However, limited tissue penetration and strain-dependent variability restrict broad applicability, indicating that PBM is best suited as an adjunct to conventional therapies rather than a replacement [37, 39].

### Limitations and future directions

This study has several limitations that need to be considered when interpreting the results. The applied single dose of 9 J/cm² was at the lower end of reported effective ranges, and higher doses are frequently required to achieve bactericidal effects [26, 29]. The use of a repeated low-dose irradiation model, while clinically relevant for wound care, complicates direct comparison with single high-dose studies.

Growth assessment was primarily based on optical density and derived AUC values, which provide indirect estimates of biomass but not viability. Another limitation of the present study is that inoculum transfer between stages was based on fixed volumes rather than normalization to OD_600_ or cell number. Although initial inoculation was standardized using a 0.5 McFarland suspension, subsequent transfers may have introduced variability in bacterial cell concentrations between stages. However, growth curves were baseline-corrected by setting the initial OD_600_ value at each stage to zero, allowing relative growth dynamics to be compared across conditions. In addition, identical handling of control and irradiated groups ensured internal comparability within each stage.

Another limitation of the present study is the limited number of independent experimental runs. The results are based on two independent experiments with four replicates per group (*n* = 4), which may restrict the generalizability and robustness of the findings. Although consistent trends were observed across both runs, further studies with increased sample size and additional repetitions are required to confirm reproducibility.

In addition, shaking of the microtiter plates was performed only intermittently prior to OD_600_ measurements and not continuously during incubation or irradiation. This may have influenced oxygen availability, sedimentation behavior, and the spatial distribution of bacterial cells within the wells, potentially affecting the uniformity of light exposure and the local irradiation dose at the cellular level. However, this approach ensured standardized measurement conditions across all experimental groups while avoiding mechanical disturbance during irradiation.

Furthermore, the chosen medium (TSB) ensured reproducibility but did not reflect physiological wound conditions; exudate-like media should be considered in future work. Variability may also have been influenced by subpopulation heterogeneity within ATCC strains, as indicated by supplier certificates. Finally, the central mechanism of porphyrin-mediated ROS generation was not directly measured, and confirmation by porphyrin quantification or ROS detection would strengthen mechanistic interpretation.

Future studies should address these limitations by employing more physiologically representative fluids (e.g. wound exudate, plasma), systematically varying irradiation parameters to define strain-specific thresholds, and ensuring comprehensive reporting of all photobiological variables [42, 43]. In vivo studies and advanced wound models are essential to evaluate tissue penetration, immune interactions, and clinical applicability [44]. Investigating long-term effects, including potential bacterial adaptation, as well as combinatory strategies with antimicrobials or debridement, may further enhance translational potential. Moreover, the impact on polymicrobial infections – common in clinical wound settings – remains largely unexplored and should be prioritized.

## Conclusion

The results demonstrated distinct strain-specific outcomes. MRSA showed immediate and significant inhibition from the first irradiation cycle through Stage_5, while *Escherichia coli* displayed delayed but significant inhibition after multiple cycles (Stage_4 to Stage_6). *Pseudomonas aeruginosa* exhibited a non-significant inhibitory trend, whereas *Enterococcus faecalis* and *Staphylococcus aureus* were unaffected under the tested conditions. Taken together, the findings demonstrate that the antimicrobial efficacy of 405 nm photobiomodulation is highly strain-dependent.

These findings address the central research questions:

- Repeated PBM reduces bacterial growth (AUC) in a strain-dependent manner.
- Repeated irradiation exerts stronger effects only in *Escherichia coli* (cumulative inhibition), while in MRSA the inhibition is immediate and stable, and absent in *Enterococcus faecalis* and *Staphylococcus aureus*.
- The effects vary between species, confirming the selective nature of PBM.

Overall, 405 nm PBM shows promise as a pathogen-specific, non-antibiotic adjunct for managing SSIs, particularly against multidrug-resistant organisms. Further research is required to optimize irradiation parameters, evaluate clinical isolates and biofilm models, and validate these findings in in-vivo settings. With standardized protocols and translational studies, 405 nm PBM has the potential to become a clinically relevant strategy for infection prevention and wound management.

## Supplementary Information

Below is the link to the electronic supplementary material.

Supplementary Material 1 Supplementary Data S1: Raw OD_600_ measurements for all strains, experimental groups and stages.

Supplementary Material 2 Supplementary Data S2: Threshold data for all strains.

## Data Availability

All data generated during this study are available within the article and its supplementary information (Supplementary material).

## References

[1] WHO. WHO looks back at 2024 World Health Organization. https://www.who.int/news-room/spotlight/who-looks-back-at-2024. Accessed 01 May 2025

[2] Murray CJL et al (2022) Global burden of bacterial antimicrobial resistance in 2019: a systematic analysis. Lancet 399(10325): 629–655. 10.1016/S0140-6736(21)02724-0 35065702 10.1016/S0140-6736(21)02724-0 PMC8841637

[3] WHO (2022) Global antimicrobial resistance and use surveillance system (GLASS) report 2022. World Health Organization Geneva. 978-92-4-006270-2 [online]. Available: https://iris.who.int/bitstream/handle/10665/364996/9789240062702-eng.pdf?sequence=1

[4] ECDC (2023) Healthcare-associated infections: surgical site infections 2018–2020. Stockholm. [online]. Available: https://www.ecdc.europa.eu/sites/default/files/documents/Healthcare-associated%20infections%20-%20surgical%20site%20infections%202018-2020.pdf

[5] ECDC (2024) Point prevalence survey of healthcare-associated infections and antimicrobial use in European acute care hospitals. European Centre for Disease Prevention and Control. Stockholm. 978-92-9498-715-0 [online]. Available: https://www.ecdc.europa.eu/sites/default/files/documents/healthcare-associated-point-prevalence-survey-acute-care-hospitals-2022-2023.pdf

[6] BMSGPK (2024) Gesundheitssystem-assoziierte Infektionen in Österreich 2022. Eine Zusammenstellung nationaler Daten. Bundesministerium für Soziales. Gesundheit. Pflege und Konsumentenschutz. 978-3-85010-693-1. [online]. Available: https://broschuerenservice.sozialministerium.at/Home/Download?publicationId=749&attachmentName=Gesundheitssystem_assoziierte_Infektionen_in_%C3%96sterreich_2022_pdfUA.pdf

[7] BMSGPK (2024) Resistenzbericht Österreich - AURES 2022 Antibiotikaresistenz und Verbrauch antimikrobieller Substanzen in Österreich. [online]. Available: https://www.sozialministerium.gv.at/dam/jcr:7872f55d-a5af-4827-b5bb-3dd865aaa903/AURES_2022.pdf

[8] WHO (2024) WHO Bacterial Priority Pathogens List. 2024: bacterial pathogens of public health importance to guide research development and strategies to prevent and control antimicrobial resistance. 978-92-4-009346-1. Accessed 14 Apr 2025. [online]. Available: https://iris.who.int/bitstream/handle/10665/376776/9789240093461-eng.pdf?sequence=1

[9] Balasubramanian R, Van Boeckel TP, Carmeli Y, Cosgrove S, Laxminarayan R (2023) Global incidence in hospital-associated infections resistant to antibiotics: an analysis of point prevalence surveys from 99 countries. PLoS Med 20(6):e1004178. 10.1371/journal.pmed.1004178 37310933 10.1371/journal.pmed.1004178 PMC10263350

[10] Mulani. MS, Kamble. EE, Kumkar. SN, Tawre. MS (2019) Pardesi. Emerging Strategies to Combat ESKAPE Pathogens in the Era of Antimicrobial Resistance: A Review. Front Microbiol vol 10:539. 10.3389/fmicb.2019.00539 10.3389/fmicb.2019.00539 PMC6452778 30988669

[11] Hamblin MR, Hasan T (2004) Photodynamic therapy: a new antimicrobial approach to infectious disease? Photochem Photobiol Sci 3(5):436–450. 10.1039/b311900a 15122361 10.1039/b311900a PMC3071049

[12] Hamblin MR, Viveiros J, Yang C, Ahmadi A, Ganz RA, Tolkoff MJ (2005) Helicobacter pylori accumulates photoactive porphyrins and is killed by visible light. Antimicrob Agents Chemother 49(7):2822–2827. 10.1128/AAC.49.7.2822-2827.2005 15980355 10.1128/AAC.49.7.2822-2827.2005 PMC1168670

[13] Jori G et al (2006) Photodynamic therapy in the treatment of microbial infections: basic principles and perspective applications. Lasers Surg Med 38(5): 468 – 81 10.1002/lsm.20361 16788934 10.1002/lsm.20361

[14] Amos-Tautua BM, Songca SP, Oluwafemi OS (2019) Application of porphyrins in antibacterial photodynamic therapy. Molecules 24(13). 10.3390/molecules24132456 10.3390/molecules24132456 PMC6650910 31277423

[15] Dai T, Gupta A, Murray CK, Vrahas MS, Tegos GP, Hamblin MR (2012) Blue light for infectious diseases: Propionibacterium acnes. Helicobacter pylori. and beyond? Drug Resist Updat 15(4): 223–36. 10.1016/j.drup.2012.07.001 10.1016/j.drup.2012.07.001 PMC3438385 22846406

[16] Halstead FD et al (2016) Antibacterial activity of blue light against nosocomial wound pathogens growing planktonically and as mature biofilms. Appl Environ Microbiol 82(13):4006–4016. 10.1128/AEM.00756-16 27129967 10.1128/AEM.00756-16 PMC4907187

[17] Kleinpenning MM, Smits T, Frunt MH, van Erp PE, van de Kerkhof PC, Gerritsen RM (2010) Clinical and histological effects of blue light on normal skin. Photodermatol Photoimmunol Photomed 26:16–21. 10.1111/j.1600-0781.2009.00474.x 20070834 10.1111/j.1600-0781.2009.00474.x

[18] Ash C, Dubec M, Donne K, Bashford T (2017) Effect of wavelength and beam width on penetration in light-tissue interaction using computational methods. Lasers Med Sci 32(8):1909–1918. 10.1007/s10103-017-2317-4 28900751 10.1007/s10103-017-2317-4 PMC5653719

[19] Maclean M, McKenzie K, Anderson JG, Gettinby G, MacGregor SJ (2014) 405 nm light technology for the inactivation of pathogens and its potential role for environmental disinfection and infection control. J Hosp Infect 88:1–11. 10.1016/j.jhin.2014.06.004 25066049 10.1016/j.jhin.2014.06.004

[20] Gwynne PJ, Gallagher MP (2018) Light as a broad-spectrum antimicrobial. Front Microbiol 9:119. 10.3389/fmicb.2018.00119 10.3389/fmicb.2018.00119 PMC5801316 29456527

[21] Perez C, Zuniga T, Palavecino CE (2021) Photodynamic therapy for treatment of Staphylococcus aureus infections. Photodiagnosis Photodyn Ther 34:102285. 10.1016/j.pdpdt.2021.102285 33836278 10.1016/j.pdpdt.2021.102285

[22] de Paoli F (2021) Low-power lasers on bacteria: stimulation, inhibition, or effectless? Lasers Med Sci 36(9):1791–1805. 10.1007/s10103-021-03258-5 33486614 10.1007/s10103-021-03258-5

[23] Bumah. VV, Masson-Meyers DS, Cashin. SE, Enwemeka CS (2013) Wavelength and bacterial density influence the bactericidal effect of blue light on methicillin-resistant Staphylococcus aureus (MRSA). Photomed Laser Surg. 31. 11. pp 547 – 53 Nov. 10.1089/pho.2012.3461 10.1089/pho.2012.3461 23621894

[24] Bumah VV, Masson-Meyers DS, Cashin S, Enwemeka CS (2015) Optimization of the antimicrobial effect of blue light on methicillin-resistant Staphylococcus aureus (MRSA) in vitro. Lasers Surg Med 47:266–272. 10.1002/lsm.22327 25639752 10.1002/lsm.22327 PMC4834034

[25] Enwemeka CS, Williams D, Hollosi S, Yens. D, Enwemeka SK (2008) Visible 405 nm SLD light photo-destroys methicillin-resistant Staphylococcus aureus (MRSA) in vitro. Lasers Surg Med 40:734–737 10.1002/lsm.20724 19065556 10.1002/lsm.20724

[26] Maclean M, MacGregor SJ, Anderson JG, Woolsey G (2009) Inactivation of bacterial pathogens following exposure to light from a 405-nanometer light-emitting diode array. Appl Environ Microbiol 75:1932–1937. 19201962 10.1128/AEM.01892-08 PMC2663198

[27] Luo S et al (2022) Understanding a defensive response of methicillin-resistant Staphylococcus aureus after exposure to multiple cycles of sub-lethal blue light. FEMS Microbiol Lett 369(1) 10.1093/femsle/fnac050 10.1093/femsle/fnac050 35675215

[28] Singh VK, Singh K, Baum K (2018) The role of methionine sulfoxide reductases in oxidative stress tolerance and virulence of staphylococcus aureus and other bacteria. Antioxidants (Basel) 7(10) 10.3390/antiox7100128 10.3390/antiox7100128 PMC6210949 30274148

[29] Lipovsky A, Nitzan Y, Gedanken A, Lubart R (2010) Visible light-induced killing of bacteria as a function of wavelength: implication for wound healing. Lasers Surg Med 42(6):467–472. 10.1002/lsm.20948 20662022 10.1002/lsm.20948

[30] Murdoch LE, Maclean M, Endarko E, MacGregor SJ, Anderson JG (2012) Bactericidal effects of 405 nm light exposure demonstrated by inactivation of Escherichia. Salmonella. Shigella. Listeria. and Mycobacterium species in liquid suspensions and on exposed surfaces. ScientificWorldJournal 2012:137805. 10.1100/2012/137805 22566760 10.1100/2012/137805 PMC3330698

[31] Gupta S, Maclean M, Anderson JG, MacGregor SJ, Meek RM, Grant MH (2015) Inactivation of micro-organisms isolated from infected lower limb arthroplasties using high-intensity narrow-spectrum (HINS) light. Bone Joint J 97–B:283–288. 10.1302/0301-620X.97B2.35154 25628296 10.1302/0301-620X.97B2.35154

[32] Imtiaz S, Bilal. M, Saleem M (2024) Antimicrobial photodynamic therapy against Escherichia coli by exploiting endogenously produced Protoporphyrin IX- In vitro study. Lasers Med Sci 39(1):204. 10.1007/s10103-024-04150-8 39088059 10.1007/s10103-024-04150-8

[33] Barneck MD et al (2016) Violet 405-nm light: a novel therapeutic agent against common pathogenic bacteria. J Surg Res 206(2):316–324. 10.1016/j.jss.2016.08.006 27884325 10.1016/j.jss.2016.08.006

[34] Anjos CD et al (2020) Antimicrobial blue light and photodynamic therapy inhibit clinically relevant beta-lactamases with extended-spectrum (ESBL) and carbapenemase activity. Photodiagnosis Photodyn Ther 32:102086. 10.1016/j.pdpdt.2020.102086 33157328 10.1016/j.pdpdt.2020.102086

[35] Rhodes NL, de la Presa M, Barneck MD, Poursaid A, Firpo MA, Langell JT (2016) Violet 405 nm light: a novel therapeutic agent against beta-lactam-resistant Escherichia coli. Lasers Surg Med 48(3):311–317. 10.1002/lsm.22457 26711625 10.1002/lsm.22457

[36] Guffey JS, Wilborn J (2006) In vitro bactericidal effects of 405-nm and 470-nm blue light. Photomed Laser Surg 24(610):684–688. 10.1089/pho.2006.24.684 17199466 10.1089/pho.2006.24.684

[37] Amin RM, Bhayana. B, Hamblin. MR, Dai T (2016) Antimicrobial blue light inactivation of Pseudomonas aeruginosa by photo-excitation of endogenous porphyrins: In vitro and in vivo studies. Lasers Surg Med 48(5):562–568. 10.1002/lsm.22474 26891084 10.1002/lsm.22474 PMC4914480

[38] Fila G, Kawiak A, Grinholc MS (2017) Blue light treatment of Pseudomonas aeruginosa: strong bactericidal activity. synergism with antibiotics and inactivation of virulence factors. Virulence 8(6):938–958. 10.1080/21505594.2016.1250995 27763824 10.1080/21505594.2016.1250995 PMC5626244

[39] Ferrer-Espada R, Wang. Y, Goh. XS, Dai T (2020) Antimicrobial blue light inactivation of microbial isolates in biofilms. Lasers Surg Med 52(5):472–478. 10.1002/lsm.23159 31536154 10.1002/lsm.23159 PMC7080594

[40] Frankenberg L, Brugna M, Hederstedt L (2002) Enterococcus faecalis heme-dependent catalase. J Bacteriol 184(22):6351–6356. 10.1128/JB.184.22.6351-6356.2002 12399505 10.1128/JB.184.22.6351-6356.2002 PMC151946

[41] Enwemeka CS, Williams D, Enwemeka SK, Hollosi S, Yens D (2009) Blue 470-nm light kills methicillin-resistant Staphylococcus aureus (MRSA) in vitro. Photomed Laser Surg 27(2):221–226 10.1089/pho.2008.2413 19196103 10.1089/pho.2008.2413

[42] Jenkins PA, Carroll JD (2011) How to report low-level laser therapy (LLLT)/photomedicine dose and beam parameters in clinical and laboratory studies. Photomed Laser Surg 29:785–787. 10.1089/pho.2011.9895 22107486 10.1089/pho.2011.9895

[43] da Fonseca AS (2019) Is there a measure for low power laser dose? Lasers Med Sci 34(1):223–234. 10.1007/s10103-018-2676-5 30402798 10.1007/s10103-018-2676-5

[44] Dhilip Kumar SS, Nadene NH, Abrahamse H (2025) Influence of biopolymer based gold nanoparticles and photobiomodulation in in vitro wound healing. Sci Rep 15(1):15793. 10.1038/s41598-025-99400-2 40328891 10.1038/s41598-025-99400-2 PMC12056154

