# Supplementary material for: Antimicrobial effects of repeated 405 nm low-level-laser photobiomodulation: an In-vitro study on surgical wound pathogens
Source: Lasers Med Sci. 2026 May 27;41(1):103. doi: 10.1007/s10103-026-04897-2 (PMC13212771; doi:10.1007/s10103-026-04897-2)
Supplement: Supplementary file 1 — Supplementary Material 1 Supplementary Data S1: Raw OD600 measurements for all strains, experimental groups and stages. [file 10103_2026_4897_MOESM1_ESM.pdf]

				Time of measurement	0 h	0 h 30 min	1 h	1 h 30 min	2 h	2 h 30 min	3 h	3 h 30 min	4 h	4 h 30 min	5 h	5 h 30 min	6 h	6 h 30 min	7 h	7 h 30 min	8 h	8 h 30 min	9 h	9 h 30 min	10 h	10 h 30 min	11 h	11 h 30 min	12 h
Pathogen	position in 12-well microtiter plate	Stage	Number of samples	experimental groups	Blank corrected based on Raw Data (600)	Blank corrected based on Raw Data (600)	Blank corrected based on Raw Data (600)	Blank corrected based on Raw Data (600)	Blank corrected based on Raw Data (600)	Blank corrected based on Raw Data (600)	Blank corrected based on Raw Data (600)	Blank corrected based on Raw Data (600)	Blank corrected based on Raw Data (600)	Blank corrected based on Raw Data (600)	Blank corrected based on Raw Data (600)	Blank corrected based on Raw Data (600)	Blank corrected based on Raw Data (600)	Blank corrected based on Raw Data (600)	Blank corrected based on Raw Data (600)	Blank corrected based on Raw Data (600)	Blank corrected based on Raw Data (600)	Blank corrected based on Raw Data (600)	Blank corrected based on Raw Data (600)	Blank corrected based on Raw Data (600)	Blank corrected based on Raw Data (600)	Blank corrected based on Raw Data (600)	Blank corrected based on Raw Data (600)	Blank corrected based on Raw Data (600)	Blank corrected based on Raw Data (600)
Enterococcus faecalis	A01	0	1	irradiated group	0,0125	0,0190	0,0295	0,0570	0,0935	0,1620	0,2690	0,3765	0,4615	0,5135	0,5240	0,5260	0,5290	0,5285	0,5290	0,5275	0,5255	0,5240	0,5225	0,5215	0,5200	0,5185	0,5190	0,5195	0,5185
Enterococcus faecalis	A02	0	1	control group	0,0115	0,0160	0,0305	0,0590	0,0975	0,1670	0,2750	0,3865	0,4735	0,5245	0,5360	0,5380	0,5410	0,5405	0,5400	0,5405	0,5385	0,5360	0,5345	0,5345	0,5340	0,5315	0,5330	0,5335	0,5335
Enterococcus faecalis	A03	0	2	irradiated group	0,0085	0,0150	0,0295	0,0600	0,0995	0,1720	0,2820	0,3925	0,4775	0,5275	0,5390	0,5410	0,5410	0,5425	0,5420	0,5415	0,5395	0,5380	0,5365	0,5355	0,5350	0,5335	0,5340	0,5345	0,5345
Enterococcus faecalis	A04	0	2	control group	0,0085	0,0150	0,0315	0,0630	0,1045	0,1810	0,2920	0,4005	0,4815	0,5285	0,5370	0,5390	0,5410	0,5405	0,5410	0,5405	0,5375	0,5350	0,5335	0,5335	0,5320	0,5305	0,5320	0,5315	0,5305
Enterococcus faecalis	C01	0	3	irradiated group	0,0155	0,0170	0,0315	0,0600	0,1025	0,1750	0,2870	0,3985	0,4825	0,5335	0,5430	0,5440	0,5460	0,5455	0,5450	0,5445	0,5415	0,5390	0,5375	0,5375	0,5370	0,5345	0,5350	0,5355	0,5345
Enterococcus faecalis	C02	0	3	control group	0,0095	0,0160	0,0305	0,0590	0,1005	0,1760	0,2870	0,4005	0,4865	0,5375	0,5450	0,5460	0,5470	0,5465	0,5460	0,5445	0,5435	0,5410	0,5385	0,5385	0,5390	0,5365	0,5380	0,5385	0,5375
Enterococcus faecalis	C03	0	4	irradiated group	0,0065	0,0160	0,0275	0,0580	0,1025	0,1780	0,2900	0,4015	0,4835	0,5325	0,5390	0,5400	0,5400	0,5405	0,5400	0,5395	0,5365	0,5350	0,5335	0,5335	0,5330	0,5315	0,5320	0,5325	0,5315
Enterococcus faecalis	C04	0	4	control group	0,0105	0,0170	0,0315	0,0660	0,1065	0,1840	0,2970	0,4065	0,4855	0,5315	0,5360	0,5380	0,5390	0,5385	0,5380	0,5375	0,5345	0,5320	0,5305	0,5305	0,5310	0,5285	0,5290	0,5295	0,5285
Enterococcus faecalis	A01	1	1	irradiated group	0,0115	0,0170	0,0330	0,0595	0,1035	0,1815	0,2800	0,3795	0,4515	0,4860	0,4915	0,4960	0,4995	0,5005	0,5005	0,5015	0,5020	0,5035	0,5045	0,5050	0,5055	0,5065	0,5070	0,5065	0,5065
Enterococcus faecalis	A02	1	1	control group	0,0085	0,0110	0,0250	0,0485	0,0865	0,1535	0,2510	0,3545	0,4365	0,4850	0,4915	0,4970	0,5025	0,5045	0,5045	0,5055	0,5070	0,5075	0,5085	0,5100	0,5105	0,5125	0,5130	0,5135	0,5135
Enterococcus faecalis	A03	1	2	irradiated group	0,0045	0,0120	0,0290	0,0575	0,1035	0,1845	0,2860	0,3885	0,4615	0,4950	0,5005	0,5060	0,5095	0,5115	0,5115	0,5125	0,5130	0,5145	0,5145	0,5160	0,5165	0,5165	0,5200	0,5185	0,5185
Enterococcus faecalis	A04	1	2	control group	0,0055	0,0100	0,0260	0,0535	0,0945	0,1675	0,2660	0,3705	0,4495	0,4900	0,4965	0,5010	0,5055	0,5075	0,5085	0,5095	0,5110	0,5115	0,5125	0,5140	0,5145	0,5155	0,5160	0,5165	0,5155
Enterococcus faecalis	C01	1	3	irradiated group	0,0065	0,0140	0,0320	0,0575	0,1025	0,1815	0,2840	0,3875	0,4625	0,4990	0,5045	0,5090	0,5125	0,5145	0,5145	0,5155	0,5160	0,5165	0,5175	0,5170	0,5175	0,5195	0,5200	0,5195	0,5195
Enterococcus faecalis	C02	1	3	control group	0,0035	0,0080	0,0230	0,0485	0,0865	0,1555	0,2550	0,3625	0,4445	0,4930	0,5005	0,5060	0,5095	0,5105	0,5115	0,5125	0,5130	0,5135	0,5155	0,5150	0,5155	0,5165	0,5190	0,5185	0,5175
Enterococcus faecalis	C03	1	4	irradiated group	0,0005	0,0110	0,0260	0,0555	0,1005	0,1815	0,2840	0,3895	0,4645	0,5000	0,5045	0,5090	0,5125	0,5135	0,5145	0,5145	0,5150	0,5155	0,5165	0,5170	0,5175	0,5185	0,5200	0,5195	0,5195
Enterococcus faecalis	C04	1	4	control group	0,0025	0,0110	0,0270	0,0565	0,0965	0,1715	0,2720	0,3785	0,4565	0,4980	0,5025	0,5070	0,5105	0,5125	0,5125	0,5135	0,5150	0,5145	0,5155	0,5170	0,5165	0,5185	0,5190	0,5185	0,5185
Enterococcus faecalis	A01	2	1	irradiated group	0,0080	0,0170	0,0320	0,0590	0,1000	0,1725	0,2680	0,3675	0,4365	0,4690	0,4715	0,4765	0,4790	0,4815	0,4815	0,4860	0,4875	0,4895	0,4870	0,4880	0,4875	0,4870	0,4885	0,4885	0,4865
Enterococcus faecalis	A02	2	1	control group	0,0070	0,0120	0,0240	0,0490	0,0860	0,1475	0,2420	0,3445	0,4245	0,4670	0,4715	0,4775	0,4810	0,4835	0,4845	0,4880	0,4905	0,4925	0,4920	0,4920	0,4925	0,4920	0,4945	0,4945	0,4915
Enterococcus faecalis	A03	2	2	irradiated group	0,0040	0,0120	0,0270	0,0550	0,0970	0,1685	0,2690	0,3715	0,4445	0,4800	0,4855	0,4905	0,4930	0,4965	0,4965	0,5010	0,5025	0,5045	0,5020	0,5020	0,5035	0,5030	0,5035	0,5035	0,5005
Enterococcus faecalis	A04	2	2	control group	0,0020	0,0100	0,0240	0,0520	0,0910	0,1585	0,2550	0,3585	0,4345	0,4730	0,4765	0,4815	0,4850	0,4885	0,4885	0,4930	0,4955	0,4975	0,4950	0,4960	0,4955	0,4960	0,4975	0,4975	0,4945
Enterococcus faecalis	C01	2	3	irradiated group	0,0060	0,0140	0,0280	0,0550	0,0950	0,1675	0,2680	0,3705	0,4455	0,4860	0,4885	0,4935	0,4960	0,4975	0,4985	0,5030	0,5045	0,5065	0,5040	0,5040	0,5035	0,5030	0,5055	0,5055	0,5025
Enterococcus faecalis	C02	2	3	control group	0,0040	0,0090	0,0220	0,0470	0,0860	0,1535	0,2510	0,3575	0,4375	0,4800	0,4855	0,4895	0,4920	0,4955	0,4965	0,5000	0,5025	0,5045	0,5030	0,5020	0,5025	0,5030	0,5035	0,5045	0,5015
Enterococcus faecalis	C03	2	4	irradiated group	0,0010	0,0110	0,0250	0,0520	0,0950	0,1675	0,2690	0,3735	0,4485	0,4840	0,4875	0,4925	0,4950	0,4965	0,4975	0,5020	0,5035	0,5055	0,5030	0,5030	0,5025	0,5030	0,5045	0,5045	0,5005
Enterococcus faecalis	C04	2	4	control group	0,0040	0,0110	0,0270	0,0550	0,0950	0,1645	0,2640	0,3695	0,4435	0,4820	0,4845	0,4895	0,4920	0,4945	0,4955	0,4990	0,5015	0,5035	0,5010	0,5010	0,5005	0,5010	0,5015	0,5025	0,4995
Enterococcus faecalis	A01	3	1	irradiated group	0,0080	0,0120	0,0290	0,0550	0,1005	0,1760	0,2735	0,3710	0,4405	0,4710	0,4745	0,4810	0,4830	0,4835	0,4850	0,4875	0,4865	0,4885	0,4890	0,4895	0,4905	0,4910	0,4930	0,4915	0,4895
Enterococcus faecalis	A02	3	1	control group	0,0070	0,0100	0,0220	0,0480	0,0855	0,1510	0,2475	0,3490	0,4275	0,4750	0,4795	0,4870	0,4910	0,4925	0,4930	0,4965	0,4975	0,4985	0,4990	0,5005	0,5015	0,5020	0,5040	0,5025	0,4995
Enterococcus faecalis	A03	3	2	irradiated group	0,0030	0,0110	0,0280	0,0560	0,1005	0,1770	0,2765	0,3750	0,4475	0,4810	0,4855	0,4920	0,4950	0,4955	0,4970	0,4995	0,5005	0,5015	0,5010	0,5025	0,5035	0,5040	0,5060	0,5045	0,5015
Enterococcus faecalis	A04	3	2	control group	0,0050	0,0110	0,0260	0,0540	0,0945	0,1640	0,2625	0,3630	0,4395	0,4790	0,4845	0,4910	0,4940	0,4965	0,4970	0,4995	0,4995	0,5005	0,5010	0,5025	0,5035	0,5050	0,5070	0,5055	0,5015
Enterococcus faecalis	C01	3	3	irradiated group	0,0070	0,0140	0,0300	0,0560	0,0995	0,1750	0,2765	0,3750	0,4475	0,4840	0,4885	0,4950	0,4970	0,4985	0,5000	0,5015	0,5025	0,5025	0,5030	0,5045	0,5055	0,5060	0,5080	0,5055	0,5025
Enterococcus faecalis	C02	3	3	control group	0,0050	0,0110	0,0240	0,0480	0,0875	0,1560	0,2555	0,3580	0,4375	0,4840	0,4895	0,4950	0,4980	0,5005	0,5020	0,5035	0,5035	0,5055	0,5060	0,5065	0,5085	0,5090	0,5100	0,5085	0,5055
Enterococcus faecalis	C03	3	4	irradiated group	0,0010	0,0110	0,0260	0,0550	0,1015	0,1790	0,2785	0,3780	0,4495	0,4820	0,4865	0,4920	0,4950	0,4965	0,4970	0,4985	0,4995	0,4995	0,5010	0,5015	0,5025	0,5030	0,5040	0,5035	0,5005
Enterococcus faecalis	C04	3	4	control group	0,0080	0,0110	0,0270	0,0560	0,0975	0,1690	0,2675	0,3680	0,4445	0,4830	0,4875	0,4940	0,4960	0,4975	0,4990	0,5015	0,5015	0,5025	0,5030	0,5045	0,5055	0,5060	0,5070	0,5055	0,5025
Enterococcus faecalis	A01	4	1	irradiated group	0,0090	0,0140	0,0280	0,0520	0,0910	0,1650	0,2590	0,3545	0,4225	0,4550	0,4605	0,4655	0,4685	0,4700	0,4705	0,4700	0,4690	0,4705	0,4720	0,4745	0,4755	0,4770	0,4780	0,4775	0,4775
Enterococcus faecalis	A02	4	1	control group	0,0070	0,0090	0,0220	0,0460	0,0820	0,1470	0,2410	0,3405	0,4185	0,4610	0,4695	0,4755	0,4775	0,4810	0,4825	0,4810	0,4800	0,4815	0,4840	0,4865	0,4885	0,4910	0,4910	0,4915	0,4905
Enterococcus faecalis	A03	4	2	irradiated group	0,0030	0,0100	0,0250	0,0510	0,0910	0,1660	0,2600	0,3565	0,4265	0,4580	0,4655	0,4715	0,4735	0,4760	0,4765	0,4750	0,4740	0,4765	0,4790	0,4805	0,4825	0,4840	0,4850	0,4855	0,4845
Enterococcus faecalis	A04	4	2	control group	0,0060	0,0100	0,0260	0,0520	0,0910	0,1610	0,2550	0,3535	0,4255	0,4610	0,4685	0,4745	0,4765	0,4790	0,4795	0,4780	0,4790	0,4795	0,4820	0,4845	0,4855	0,4880	0,4880	0,4885	0,4865
Enterococcus faecalis	C01	4	3	irradiated group	0,0060	0,0120	0,0290	0,0550	0,0940	0,1700	0,2660	0,3625	0,4305	0,4640	0,4705	0,4765	0,4775	0,4800	0,4805	0,4790	0,4790	0,4805	0,4820	0,4835	0,4845	0,4870	0,4870	0,4885	0,4875
Enterococcus faecalis	C02	4	3	control group	0,0040	0,0080	0,0220	0,0470	0,0850	0,1540	0,2490	0,3515	0,4275	0,4690	0,4755	0,4815	0,4825	0,4860	0,4855	0,4840	0,4850	0,4865	0,4890	0,4905	0,4915	0,4930	0,4950	0,4945	0,4935
Enterococcus faecalis	C03	4	4	irradiated group	0,0030	0,0090	0,0270	0,0500	0,0940	0,1710	0,2670	0,3645	0,4335	0,4650	0,4715	0,4765	0,4775	0,4800	0,4795	0,4780	0,4790	0,4805	0,4820	0,4845	0,4855	0,4870	0,4880	0,4885	0,4875
Enterococcus faecalis	C04	4	4	control group	0,0040	0,0110	0,0260	0,0540	0,0920	0,1640	0,2590	0,3585	0,4305	0,4670	0,4735	0,4795	0,4805	0,4830	0,4835	0,4820	0,4820	0,4835	0,4860	0,4875	0,4895	0,4900	0,4920	0,4915	0,4905
Enterococcus faecalis	A01	5	1	irradiated group	0,0065	0,0155	0,0310	0,0590	0,1035	0,1820	0,2815	0,3770	0,4455	0,4720	0,4760	0,4825	0,4855	0,4885	0,4890	0,4900	0,4920	0,4940	0,4950	0,4950	0,4965	0,4965	0,4970	0,4960	0,4960
Enterococcus faecalis	A02	5	1	control group	0,0065	0,0095	0,0210	0,0460	0,0845	0,1510	0,2475	0,3480	0,4285	0,4720	0,4780	0,4855	0,4895	0,4935	0,4950	0,4960	0,4980	0,5000	0,5010	0,5020	0,5025	0,5035	0,5040	0,5030	0,5020
Enterococcus faecalis	A03	5	2	irradiated group	0,0005	0,0085	0,0260	0,0530	0,0985	0,1740	0,2735	0,3720	0,4465	0,4770	0,4840	0,4905	0,4945	0,4975	0,4980	0,5000	0,5020	0,5030	0,5050	0,5060	0,5055	0,5065	0,5080	0,5060	0,5050
Enterococcus faecalis	A04	5	2	control group	0,0035	0,0105	0,0260	0,0530	0,0935	0,1640	0,2615	0,3620	0,4395	0,4740	0,4820	0,4885	0,4925	0,4955	0,4970	0,4980	0,5000	0,5020	0,5020	0,5030	0,5045	0,5045	0,5060	0,5040	0,5040
Enterococcus faecalis	C01	5	3	irradiated group	0,0045	0,0125	0,0290	0,0570	0,1035	0,1790	0,2795	0,3760	0,4495	0,4810	0,4870	0,4925	0,4975	0,4985	0,5000	0,5010	0,5030	0,5040	0,5050	0,5060	0,5065	0,5065	0,5080	0,5070	0,5070
Enterococcus faecalis	C02	5	3	control group	0,0035	0,0105	0,0220	0,0500	0,0895	0,1570	0,2565	0,3600	0,4385	0,4820	0,4860	0,4915	0,4955	0,4995	0,5000	0,5010	0,5030	0,5050	0,5060	0,5070	0,5085	0,5085	0,5090	0,5080	0,5080
Enterococcus faecalis	C03	5	4	irradiated group	0,0005	0,0125	0,0270	0,0560	0,1045	0,1840	0,2845	0,3830	0,4545	0,4830	0,4880	0,4925	0,4965	0,4985	0,5000	0,5010	0,5030	0,5040	0,5050	0,5060	0,5065	0,5075	0,5080	0,5070	0,5060
Enterococcus faecalis	C04	5	4	control group	0,0045	0,0125	0,0270	0,0570	0,0975	0,1690	0,2645	0,3670	0,4445	0,4800	0,4860	0,4905	0,4955	0,4985	0,4990	0,5010	0,5030	0,5040	0,5040	0,5060	0,5065	0,5075	0,5080	0,5070	0,5060
Enterococcus faecalis	A01	6	1	irradiated group	0,0070	0,0155	0,0295	0,0550	0,0995	0,1745	0,2715	0,3685	0,4425	0,4735	0,4790	0,4815	0,4835	0,4855	0,4850	0,4845	0,4845	0,4850	0,4845	0,4860	0,4850	0,4855	0,4865	0,4870	0,4865
Enterococcus faecalis	A02	6	1	control group	0,0030	0,0085	0,0195	0,0440	0,0825	0,1465	0,2385	0,3385	0,4175	0,4605	0,4680	0,4705	0,4735	0,4755	0,4760	0,4755	0,4765	0,4770	0,4785	0,4780	0,4780	0,4785	0,4795	0,4810	0,4785
Enterococcus faecalis	A03	6	2	irradiated group	0,0020	0,0125	0,0275	0,0560	0,0995	0,1755	0,2755	0,3735	0,4485	0,4795	0,4880	0,4905	0,4915	0,4945	0,4940	0,4935	0,4935	0,4950	0,4945	0,4950	0,4940	0,4945	0,4955	0,4960	0,4955
Enterococcus faecalis	A04	6	2	control group	0,0040	0,0115	0,0255	0,0530	0,0925	0,1615	0,2595	0,3615	0,4395	0,4785	0,4860	0,4905	0,4925	0,4945	0,4950	0,4935	0,4935	0,4950	0,4945	0,4950	0,4950	0,4965	0,4965	0,4970	0,4965
Enterococcus faecalis	C01	6	3	irradiated group	0,0060	0,0145	0,0285	0,0570	0,0995	0,1745	0,2735	0,3725	0,4485	0,4845	0,4920	0,4945	0,4965	0,4975	0,4980	0,4965	0,4965	0,4970	0,4975	0,4980	0,4980	0,4985	0,4985	0,4990	0,4985
Enterococcus faecalis	C02	6	3	control group	0,0030	0,0085	0,0215	0,0480	0,0875	0,1565	0,2555	0,3595	0,4405	0,4835	0,4900	0,4925	0,4955	0,4965	0,4970	0,4965	0,4965	0,4970	0,4975	0,4980	0,4970	0,4975	0,4985	0,4990	0,4995
Enterococcus faecalis	C03	6	4	irradiated group	0,0000	0,0105	0,0275	0,0530	0,0965	0,1735	0,2735	0,3745	0,4505	0,4825	0,4890	0,4915	0,4935	0,4945	0,4950	0,4935	0,4935	0,4950	0,4945	0,4940	0,4950	0,4955	0,4955	0,4970	0,4955
Enterococcus faecalis	C04	6	4	control group	0,0030	0,0125	0,0265	0,0560	0,0955	0,1645	0,2635	0,3655	0,4435	0,4805	0,4880	0,4905	0,4925	0,4945	0,4940	0,4935	0,4935	0,4950	0,4945	0,4950	0,4960	0,4965	0,4965	0,4970	0,4965
Enterococcus faecalis	A01	0	5	irradiated group	0,0100	0,0140	0,0245	0,0480	0,0815	0,1425	0,2425	0,3590	0,4670	0,5365	0,5570	0,5660	0,5735	0,5765	0,5795	0,5815	0,5835	0,5860	0,5885	0,5860	0,5840	0,5835	0,5830	0,5830	0,5840
Enterococcus faecalis	A02	0	5	control group	0,0040	0,0110	0,0205	0,0470	0,0815	0,1425	0,2425	0,3600	0,4680	0,5365	0,5560	0,5640	0,5715	0,5755	0,5785	0,5815	0,5835	0,5850	0,5885	0,5870	0,5860	0,5875	0,5850	0,5870	0,5870
Enterococcus faecalis	A03	0	6	irradiated group	0,0020	0,0100	0,0215	0,0490	0,0835	0,1465	0,2475	0,3620	0,4690	0,5355	0,5500	0,5590	0,5665	0,5715	0,5735	0,5765	0,5795	0,5820	0,5835	0,5820	0,5820	0,5825	0,5820	0,5820	0,5830
Enterococcus faecalis	A04	0	6	control group	0,0010	0,0110	0,0225	0,0490	0,0835	0,1455	0,2415	0,3510	0,4530	0,5185	0,5350	0,5450	0,5535	0,5565	0,5595	0,5625	0,5635	0,5650	0,5655	0,5650	0,5640	0,5645	0,5630	0,5630	0,5640
Enterococcus faecalis	C01	0	7	irradiated group	0,0090	0,0130	0,0255	0,0500	0,0845	0,1495	0,2515	0,3670	0,4740	0,5425	0,5620	0,5690	0,5745	0,5805	0,5825	0,5845	0,5865	0,5880	0,5905	0,5880	0,5880	0,5875	0,5860	0,5860	0,5870
Enterococcus faecalis	C02	0	7	control group	0,0040	0,0100	0,0225	0,0490	0,0845	0,1515	0,2545	0,3720	0,4760	0,5425	0,5610	0,5680	0,5745	0,5785	0,5815	0,5845	0,5855	0,5870	0,5895	0,5870	0,5870	0,5875	0,5860	0,5860	0,5870
Enterococcus faecalis	C03	0	8	irradiated group	0,0020	0,0110	0,0235	0,0520	0,0895	0,1565	0,2625	0,3800	0,4840	0,5475	0,5620	0,5690	0,5765	0,5795	0,5835	0,5865	0,5875	0,5890	0,5915	0,5900	0,5890	0,5885	0,5880	0,5880	0,5890
Enterococcus faecalis	C04	0	8	control group	0,0060	0,0150	0,0285	0,0580	0,0935	0,1645	0,2695	0,3830	0,4840	0,5455	0,5590	0,5660	0,5725	0,5765	0,5795	0,5815	0,5845	0,5840	0,5865	0,5850	0,5840	0,5835	0,5840	0,5830	0,5840
Enterococcus faecalis	A01	1	5	irradiated group	0,0100	0,0150	0,0305	0,0565	0,0990	0,1750	0,2805	0,3915	0,4820	0,5270	0,5330	0,5420	0,5465	0,5485	0,5495	0,5500	0,5505	0,5515	0,5545	0,5545	0,5540	0,5535	0,5535	0,5530	0,5530
Enterococcus faecalis	A02	1	5	control group	0,0080	0,0120	0,0265	0,0535	0,0930	0,1640	0,2685	0,3825	0,4780	0,5310	0,5360	0,5470	0,5505	0,5525	0,5555	0,5570	0,5575	0,5605	0,5635	0,5645	0,5630	0,5625	0,5635	0,5630	0,5620
Enterococcus faecalis	A03	1	6	irradiated group	0,0030	0,0120	0,0275	0,0555	0,0990	0,1770	0,2815	0,3945	0,4830	0,5270	0,5330	0,5430	0,5475	0,5485	0,5525	0,5510	0,5525	0,5555	0,5585	0,5595	0,5600	0,5585	0,5595	0,5590	0,5580
Enterococcus faecalis	A04	1	6	control group	0,0050	0,0130	0,0275	0,0555	0,0980	0,1710	0,2735	0,3875	0,4790	0,5280	0,5360	0,5460	0,5505	0,5515	0,5545	0,5560	0,5565	0,5595	0,5615	0,5615	0,5600	0,5585	0,5605	0,5600	0,5590
Enterococcus faecalis	C01	1	7	irradiated group	0,0070	0,0170	0,0345	0,0635	0,1110	0,1930	0,3025	0,4165	0,5040	0,5420	0,5490	0,5560	0,5605	0,5615	0,5625	0,5650	0,5645	0,5645	0,5665	0,5675	0,5680	0,5685	0,5695	0,5670	0,5670
Enterococcus faecalis	C02	1	7	control group	0,0040	0,0120	0,0265	0,0555	0,0960	0,1720	0,2765	0,3935	0,4890	0,5410	0,5480	0,5560	0,5615	0,5635	0,5645	0,5670	0,5685	0,5685	0,5705	0,5715	0,5710	0,5695	0,5705	0,5700	0,5680
Enterococcus faecalis	C03	1	8	irradiated group	0,0030	0,0120	0,0305	0,0585	0,1070	0,1900	0,2975	0,4125	0,5010	0,5400	0,5470	0,5540	0,5585	0,5615	0,5605	0,5640	0,5635	0,5655	0,5675	0,5675	0,5680	0,5675	0,5685	0,5680	0,5670
Enterococcus faecalis	C04	1	8	control group	0,0040	0,0120	0,0305	0,0615	0,1030	0,1780	0,2815	0,3975	0,4890	0,5380	0,5450	0,5520	0,5565	0,5595	0,5605	0,5630	0,5635	0,5645	0,5665	0,5665	0,5670	0,5645	0,5655	0,5650	0,5640
Enterococcus faecalis	A01	2	5	irradiated group	0,0150	0,0210	0,0380	0,0630	0,1045	0,1770	0,2840	0,3955	0,4830	0,5310	0,5390	0,5445	0,5495	0,5510	0,5520	0,5535	0,5545	0,5545	0,5555	0,5570	0,5580	0,5575	0,5570	0,5575	0,5550
Enterococcus faecalis	A02	2	5	control group	0,0070	0,0110	0,0250	0,0530	0,0905	0,1600	0,2630	0,3795	0,4750	0,5330	0,5400	0,5455	0,5515	0,5520	0,5540	0,5565	0,5575	0,5595	0,5605	0,5620	0,5640	0,5635	0,5620	0,5645	0,5630
Enterococcus faecalis	A03	2	6	irradiated group	0,0060	0,0160	0,0310	0,0600	0,1035	0,1800	0,2860	0,3995	0,4850	0,5280	0,5370	0,5435	0,5495	0,5520	0,5540	0,5565	0,5565	0,5585	0,5595	0,5600	0,5610	0,5585	0,5580	0,5585	0,5570
Enterococcus faecalis	A04	2	6	control group	0,0020	0,0080	0,0250	0,0550	0,0955	0,1660	0,2700	0,3865	0,4780	0,5310	0,5370	0,5425	0,5475	0,5490	0,5510	0,5535	0,5545	0,5565	0,5585	0,5590	0,5610	0,5605	0,5590	0,5605	0,5590
Enterococcus faecalis	C01	2	7	irradiated group	0,0110	0,0200	0,0350	0,0630	0,1075	0,1840	0,2920	0,4065	0,4950	0,5410	0,5470	0,5545	0,5585	0,5590	0,5610	0,5625	0,5635	0,5635	0,5655	0,5670	0,5680	0,5675	0,5660	0,5675	0,5660
Enterococcus faecalis	C02	2	7	control group	0,0050	0,0120	0,0290	0,0590	0,1035	0,1820	0,2900	0,4085	0,4970	0,5420	0,5500	0,5535	0,5585	0,5580	0,5610	0,5635	0,5645	0,5655	0,5665	0,5690	0,5700	0,5695	0,5700	0,5715	0,5700
Enterococcus faecalis	C03	2	8	irradiated group	0,0040	0,0140	0,0320	0,0600	0,1055	0,1830	0,2910	0,4065	0,4920	0,5340	0,5400	0,5465	0,5525	0,5520	0,5540	0,5555	0,5565	0,5585	0,5585	0,5590	0,5610	0,5605	0,5590	0,5605	0,5590
Enterococcus faecalis	C04	2	8	control group	0,0020	0,0100	0,0270	0,0590	0,1025	0,1750	0,2800	0,3975	0,4880	0,5410	0,5470	0,5515	0,5555	0,5570	0,5590	0,5605	0,5615	0,5625	0,5635	0,5650	0,5670	0,5665	0,5670	0,5675	0,5660
Enterococcus faecalis	A01	3	5	irradiated group	0,0095	0,0185	0,0340	0,0605	0,1015	0,1800	0,2870	0,3990	0,4870	0,5330	0,5410	0,5485	0,5535	0,5555	0,5595	0,5595	0,5590	0,5600	0,5605	0,5615	0,5635	0,5650	0,5665	0,5675	0,5655
Enterococcus faecalis	A02	3	5	control group	0,0055	0,0115	0,0260	0,0535	0,0915	0,1620	0,2630	0,3790	0,4740	0,5310	0,5380	0,5455	0,5525	0,5555	0,5585	0,5595	0,5610	0,5630	0,5635	0,5645	0,5665	0,5690	0,5695	0,5695	0,5675
Enterococcus faecalis	A03	3	6	irradiated group	-0,0015	0,0105	0,0280	0,0555	0,0995	0,1810	0,2860	0,4020	0,4910	0,5350	0,5420	0,5495	0,5555	0,5565	0,5615	0,5625	0,5640	0,5670	0,5675	0,5675	0,5695	0,5730	0,5745	0,5735	0,5715
Enterococcus faecalis	A04	3	6	control group	0,0005	0,0095	0,0250	0,0545	0,0945	0,1670	0,2700	0,3870	0,4800	0,5330	0,5410	0,5485	0,5535	0,5575	0,5615	0,5615	0,5640	0,5650	0,5665	0,5675	0,5685	0,5700	0,5705	0,5695	0,5685
Enterococcus faecalis	C01	3	7	irradiated group	0,0045	0,0155	0,0320	0,0605	0,1045	0,1850	0,2940	0,4100	0,5000	0,5460	0,5540	0,5605	0,5655	0,5675	0,5715	0,5715	0,5720	0,5730	0,5735	0,5735	0,5755	0,5760	0,5785	0,5805	0,5785
Enterococcus faecalis	C02	3	7	control group	0,0005	0,0085	0,0240	0,0495	0,0895	0,1620	0,2640	0,3810	0,4760	0,5330	0,5410	0,5485	0,5535	0,5575	0,5605	0,5605	0,5620	0,5640	0,5635	0,5645	0,5665	0,5690	0,5685	0,5685	0,5665
Enterococcus faecalis	C03	3	8	irradiated group	-0,0025	0,0115	0,0280	0,0585	0,1045	0,1870	0,2970	0,4140	0,5030	0,5450	0,5530	0,5585	0,5645	0,5665	0,5705	0,5705	0,5720	0,5740	0,5735	0,5735	0,5745	0,5780	0,5795	0,5805	0,5795
Enterococcus faecalis	C04	3	8	control group	0,0005	0,0105	0,0260	0,0555	0,0985	0,1750	0,2790	0,3960	0,4850	0,5320	0,5410	0,5465	0,5525	0,5555	0,5595	0,5585	0,5610	0,5620	0,5615	0,5625	0,5635	0,5670	0,5665	0,5655	0,5635
Enterococcus faecalis	A01	4	5	irradiated group	0,0110	0,0165	0,0305	0,0575	0,1000	0,1770	0,2820	0,3950	0,4845	0,5320	0,5390	0,5470	0,5510	0,5540	0,5545	0,5560	0,5580	0,5590	0,5605	0,5610	0,5610	0,5620	0,5620	0,5620	0,5610
Enterococcus faecalis	A02	4	5	control group	0,0070	0,0115	0,0265	0,0535	0,0930	0,1630	0,2670	0,3840	0,4795	0,5340	0,5400	0,5490	0,5530	0,5550	0,5575	0,5580	0,5620	0,5630	0,5645	0,5650	0,5670	0,5690	0,5670	0,5670	0,5670
Enterococcus faecalis	A03	4	6	irradiated group	0,0040	0,0155	0,0305	0,0585	0,1030	0,1790	0,2850	0,4000	0,4875	0,5330	0,5400	0,5480	0,5520	0,5550	0,5565	0,5580	0,5620	0,5620	0,5635	0,5650	0,5660	0,5680	0,5680	0,5670	0,5670
Enterococcus faecalis	A04	4	6	control group	0,0060	0,0155	0,0315	0,0615	0,1020	0,1740	0,2760	0,3930	0,4815	0,5310	0,5390	0,5460	0,5510	0,5550	0,5575	0,5570	0,5600	0,5620	0,5625	0,5640	0,5640	0,5640	0,5630	0,5630	0,5620
Enterococcus faecalis	C01	4	7	irradiated group	0,0080	0,0175	0,0345	0,0615	0,1070	0,1850	0,2940	0,4090	0,4995	0,5470	0,5540	0,5600	0,5640	0,5670	0,5685	0,5680	0,5700	0,5710	0,5715	0,5740	0,5740	0,5750	0,5760	0,5750	0,5740
Enterococcus faecalis	C02	4	7	control group	0,0060	0,0125	0,0275	0,0535	0,0950	0,1680	0,2720	0,3910	0,4845	0,5420	0,5490	0,5560	0,5610	0,5640	0,5665	0,5660	0,5690	0,5690	0,5705	0,5730	0,5730	0,5740	0,5730	0,5720	0,5720
Enterococcus faecalis	C03	4	8	irradiated group	0,0040	0,0145	0,0325	0,0605	0,1080	0,1880	0,2970	0,4140	0,5015	0,5440	0,5510	0,5570	0,5620	0,5640	0,5665	0,5660	0,5690	0,5700	0,5705	0,5730	0,5720	0,5730	0,5740	0,5750	0,5730
Enterococcus faecalis	C04	4	8	control group	0,0090	0,0185	0,0355	0,0655	0,1100	0,1870	0,2900	0,4080	0,4945	0,5400	0,5480	0,5530	0,5590	0,5610	0,5635	0,5630	0,5660	0,5660	0,5665	0,5690	0,5690	0,5700	0,5690	0,5680	0,5670
Enterococcus faecalis	A01	5	5	irradiated group	0,0105	0,0170	0,0325	0,0600	0,1040	0,1820	0,2860	0,4025	0,4880	0,5310	0,5375	0,5460	0,5495	0,5540	0,5550	0,5560	0,5570	0,5590	0,5580	0,5620	0,5620	0,5635	0,5625	0,5620	0,5595
Enterococcus faecalis	A02	5	5	control group	0,0065	0,0120	0,0245	0,0520	0,0920	0,1610	0,2650	0,3835	0,4780	0,5340	0,5395	0,5480	0,5525	0,5570	0,5600	0,5620	0,5640	0,5670	0,5660	0,5690	0,5710	0,5725	0,5705	0,5700	0,5675
Enterococcus faecalis	A03	5	6	irradiated group	-0,0005	0,0110	0,0265	0,0560	0,1020	0,1810	0,2880	0,4055	0,4930	0,5340	0,5395	0,5490	0,5535	0,5560	0,5580	0,5600	0,5630	0,5650	0,5640	0,5670	0,5680	0,5705	0,5705	0,5700	0,5675
Enterococcus faecalis	A04	5	6	control group	0,0005	0,0100	0,0245	0,0540	0,0970	0,1700	0,2730	0,3885	0,4790	0,5270	0,5345	0,5430	0,5475	0,5530	0,5540	0,5580	0,5590	0,5610	0,5590	0,5620	0,5640	0,5655	0,5625	0,5630	0,5595
Enterococcus faecalis	C01	5	7	irradiated group	0,0065	0,0140	0,0315	0,0610	0,1070	0,1870	0,2950	0,4115	0,5000	0,5430	0,5495	0,5570	0,5605	0,5650	0,5660	0,5680	0,5680	0,5700	0,5700	0,5720	0,5720	0,5745	0,5735	0,5750	0,5715
Enterococcus faecalis	C02	5	7	control group	0,0035	0,0110	0,0255	0,0520	0,0930	0,1620	0,2680	0,3865	0,4820	0,5410	0,5485	0,5560	0,5605	0,5650	0,5670	0,5700	0,5700	0,5730	0,5720	0,5740	0,5760	0,5775	0,5755	0,5750	0,5735
Enterococcus faecalis	C03	5	8	irradiated group	0,0015	0,0110	0,0285	0,0580	0,1050	0,1860	0,2950	0,4125	0,4980	0,5380	0,5455	0,5520	0,5555	0,5600	0,5610	0,5640	0,5650	0,5650	0,5650	0,5670	0,5690	0,5715	0,5695	0,5710	0,5675
Enterococcus faecalis	C04	5	8	control group	0,0025	0,0120	0,0295	0,0580	0,1020	0,1760	0,2810	0,3985	0,4900	0,5390	0,5465	0,5540	0,5585	0,5630	0,5640	0,5670	0,5680	0,5690	0,5680	0,5700	0,5710	0,5735	0,5705	0,5700	0,5665
Enterococcus faecalis	A01	6	5	irradiated group	0,0080	0,0150	0,0290	0,0560	0,0990	0,1735	0,2785	0,3920	0,4785	0,5255	0,5315	0,5410	0,5445	0,5480	0,5485	0,5520	0,5525	0,5545	0,5530	0,5540	0,5555	0,5565	0,5575	0,5555	0,5545
Enterococcus faecalis	A02	6	5	control group	0,0050	0,0120	0,0250	0,0520	0,0920	0,1605	0,2635	0,3810	0,4735	0,5315	0,5355	0,5460	0,5505	0,5540	0,5565	0,5580	0,5605	0,5645	0,5630	0,5650	0,5665	0,5665	0,5655	0,5645	0,5635
Enterococcus faecalis	A03	6	6	irradiated group	-0,0010	0,0120	0,0270	0,0560	0,1000	0,1765	0,2805	0,3940	0,4815	0,5265	0,5325	0,5410	0,5455	0,5480	0,5505	0,5530	0,5555	0,5575	0,5580	0,5590	0,5605	0,5615	0,5615	0,5605	0,5595
Enterococcus faecalis	A04	6	6	control group	0,0010	0,0120	0,0270	0,0570	0,0980	0,1695	0,2705	0,3860	0,4765	0,5285	0,5345	0,5440	0,5485	0,5520	0,5555	0,5580	0,5595	0,5615	0,5610	0,5620	0,5635	0,5635	0,5615	0,5605	0,5595
Enterococcus faecalis	C01	6	7	irradiated group	0,0050	0,0140	0,0310	0,0590	0,1020	0,1795	0,2875	0,4020	0,4895	0,5375	0,5435	0,5520	0,5565	0,5590	0,5625	0,5630	0,5635	0,5655	0,5640	0,5660	0,5665	0,5685	0,5685	0,5675	0,5655
Enterococcus faecalis	C02	6	7	control group	0,0040	0,0120	0,0260	0,0550	0,0950	0,1665	0,2715	0,3900	0,4845	0,5405	0,5465	0,5540	0,5585	0,5630	0,5665	0,5690	0,5695	0,5705	0,5700	0,5710	0,5715	0,5725	0,5715	0,5705	0,5695
Enterococcus faecalis	C03	6	8	irradiated group	-0,0010	0,0120	0,0290	0,0590	0,1080	0,1875	0,2965	0,4120	0,4965	0,5375	0,5435	0,5500	0,5535	0,5570	0,5615	0,5630	0,5635	0,5655	0,5640	0,5650	0,5655	0,5675	0,5685	0,5675	0,5665
Enterococcus faecalis	C04	6	8	control group	0,0040	0,0150	0,0330	0,0630	0,1060	0,1785	0,2825	0,4000	0,4885	0,5375	0,5445	0,5520	0,5555	0,5600	0,5625	0,5650	0,5655	0,5665	0,5660	0,5670	0,5685	0,5685	0,5675	0,5665	0,5665

				Messzeitpunkt	0 h	0 h 30 min	1 h	1 h 30 min	2 h	2 h 30 min	3 h	3 h 30 min	4 h	4 h 30 min	5 h	5 h 30 min	6 h	6 h 30 min	7 h	7 h 30 min	8 h	8 h 30 min	9 h	9 h 30 min	10 h	10 h 30 min	11 h	11 h 30 min	12 h
Erreger	Position - 12-Well-Mikro-titerplatte	Stage	Stichproben-anzahl	Versuchsgruppe	Blank corrected based on Raw Data (600)	Blank corrected based on Raw Data (600)	Blank corrected based on Raw Data (600)	Blank corrected based on Raw Data (600)	Blank corrected based on Raw Data (600)	Blank corrected based on Raw Data (600)	Blank corrected based on Raw Data (600)	Blank corrected based on Raw Data (600)	Blank corrected based on Raw Data (600)	Blank corrected based on Raw Data (600)	Blank corrected based on Raw Data (600)	Blank corrected based on Raw Data (600)	Blank corrected based on Raw Data (600)	Blank corrected based on Raw Data (600)	Blank corrected based on Raw Data (600)	Blank corrected based on Raw Data (600)	Blank corrected based on Raw Data (600)	Blank corrected based on Raw Data (600)	Blank corrected based on Raw Data (600)	Blank corrected based on Raw Data (600)	Blank corrected based on Raw Data (600)	Blank corrected based on Raw Data (600)	Blank corrected based on Raw Data (600)	Blank corrected based on Raw Data (600)	Blank corrected based on Raw Data (600)
Escherichia coli	A01	0	1	irradiated group	0,0095	0,0130	0,0160	0,0295	0,0585	0,1040	0,1730	0,2570	0,3355	0,4155	0,4765	0,5015	0,5090	0,5115	0,5130	0,5150	0,5165	0,5170	0,5255	0,5245	0,5285	0,5330	0,5360	0,5375	0,5395
Escherichia coli	A02	0	1	control group	0,0075	0,0110	0,0160	0,0285	0,0585	0,1060	0,1780	0,2570	0,3385	0,4225	0,4855	0,5135	0,5200	0,5215	0,5210	0,5220	0,5235	0,5260	0,5305	0,5325	0,5355	0,5390	0,5420	0,5445	0,5465
Escherichia coli	A03	0	2	irradiated group	0,0035	0,0080	0,0130	0,0265	0,0575	0,1080	0,1780	0,2570	0,3465	0,4235	0,4885	0,5135	0,5210	0,5215	0,5210	0,5210	0,5225	0,5250	0,5275	0,5305	0,5345	0,5370	0,5410	0,5425	0,5465
Escherichia coli	A04	0	2	control group	0,0035	0,0090	0,0130	0,0285	0,0615	0,1130	0,1840	0,2630	0,3495	0,4265	0,4875	0,5125	0,5200	0,5185	0,5170	0,5180	0,5185	0,5210	0,5265	0,5285	0,5325	0,5350	0,5370	0,5415	0,5445
Escherichia coli	C01	0	3	irradiated group	0,0095	0,0090	0,0150	0,0285	0,0595	0,1070	0,1790	0,2610	0,3445	0,4305	0,4845	0,5105	0,5200	0,5205	0,5210	0,5220	0,5225	0,5250	0,5295	0,5325	0,5355	0,5390	0,5430	0,5455	0,5475
Escherichia coli	C02	0	3	control group	0,0025	0,0080	0,0110	0,0275	0,0575	0,1080	0,1810	0,2640	0,3405	0,4295	0,4865	0,5115	0,5190	0,5185	0,5210	0,5210	0,5205	0,5250	0,5315	0,5335	0,5355	0,5400	0,5410	0,5445	0,5465
Escherichia coli	C03	0	4	irradiated group	0,0005	0,0060	0,0100	0,0255	0,0595	0,1090	0,1830	0,2670	0,3535	0,4315	0,4905	0,5135	0,5210	0,5195	0,5190	0,5200	0,5205	0,5260	0,5285	0,5315	0,5345	0,5380	0,5400	0,5445	0,5465
Escherichia coli	C04	0	4	control group	0,0005	0,0090	0,0130	0,0315	0,0635	0,1160	0,1900	0,2660	0,3555	0,4355	0,4895	0,5135	0,5180	0,5185	0,5210	0,5180	0,5205	0,5260	0,5265	0,5295	0,5325	0,5360	0,5400	0,5435	0,5445
Escherichia coli	A01	1	1	irradiated group	0,0090	0,0190	0,0305	0,0440	0,0915	0,1560	0,2290	0,3160	0,4050	0,4740	0,4990	0,5105	0,5085	0,5095	0,5090	0,5080	0,5090	0,5115	0,5145	0,5170	0,5185	0,5210	0,5230	0,5250	0,5280
Escherichia coli	A02	1	1	control group	0,0070	0,0120	0,0255	0,0450	0,0705	0,1380	0,2150	0,3010	0,3960	0,4780	0,5100	0,5225	0,5205	0,5195	0,5190	0,5200	0,5220	0,5235	0,5265	0,5290	0,5305	0,5330	0,5360	0,5360	0,5390
Escherichia coli	A03	1	2	irradiated group	0,0040	0,0140	0,0275	0,0430	0,0845	0,1460	0,2200	0,3160	0,4070	0,4800	0,5060	0,5185	0,5145	0,5145	0,5130	0,5120	0,5130	0,5145	0,5155	0,5180	0,5205	0,5230	0,5250	0,5260	0,5270
Escherichia coli	A04	1	2	control group	0,0070	0,0150	0,0315	0,0490	0,0855	0,1420	0,2330	0,3270	0,4140	0,4850	0,5140	0,5245	0,5205	0,5205	0,5200	0,5180	0,5210	0,5225	0,5245	0,5260	0,5285	0,5300	0,5330	0,5330	0,5350
Escherichia coli	C01	1	3	irradiated group	0,0090	0,0170	0,0305	0,0470	0,0785	0,1450	0,2320	0,3160	0,4150	0,4810	0,5080	0,5185	0,5165	0,5165	0,5150	0,5140	0,5150	0,5175	0,5205	0,5220	0,5235	0,5260	0,5290	0,5290	0,5310
Escherichia coli	C02	1	3	control group	0,0070	0,0130	0,0275	0,0420	0,0815	0,1480	0,2190	0,3120	0,4030	0,4820	0,5140	0,5255	0,5225	0,5225	0,5220	0,5220	0,5250	0,5265	0,5285	0,5310	0,5335	0,5350	0,5380	0,5380	0,5400
Escherichia coli	C03	1	4	irradiated group	0,0060	0,0150	0,0295	0,0390	0,0765	0,1410	0,2350	0,3190	0,4160	0,4840	0,5120	0,5225	0,5185	0,5175	0,5170	0,5170	0,5180	0,5195	0,5215	0,5250	0,5265	0,5290	0,5310	0,5310	0,5340
Escherichia coli	C04	1	4	control group	0,0090	0,0160	0,0325	0,0480	0,0805	0,1640	0,2440	0,3200	0,4200	0,4870	0,5170	0,5265	0,5215	0,5205	0,5200	0,5210	0,5230	0,5245	0,5275	0,5290	0,5315	0,5340	0,5360	0,5360	0,5380
Escherichia coli	A01	2	1	irradiated group	0,0075	0,0160	0,0315	0,0535	0,0895	0,1620	0,2435	0,3345	0,4255	0,4830	0,5005	0,5090	0,5130	0,5135	0,5125	0,5140	0,5175	0,5205	0,5250	0,5285	0,5305	0,5330	0,5365	0,5395	0,5475
Escherichia coli	A02	2	1	control group	0,0055	0,0130	0,0265	0,0525	0,0735	0,1420	0,2215	0,3115	0,4085	0,4800	0,5055	0,5170	0,5200	0,5195	0,5185	0,5200	0,5235	0,5275	0,5310	0,5335	0,5365	0,5390	0,5415	0,5455	0,5535
Escherichia coli	A03	2	2	irradiated group	0,0015	0,0130	0,0295	0,0495	0,0905	0,1510	0,2395	0,3405	0,4275	0,4880	0,5085	0,5180	0,5190	0,5195	0,5175	0,5190	0,5205	0,5235	0,5270	0,5315	0,5335	0,5360	0,5375	0,5425	0,5495
Escherichia coli	A04	2	2	control group	0,0055	0,0170	0,0335	0,0565	0,0895	0,1500	0,2385	0,3335	0,4245	0,4900	0,5105	0,5210	0,5220	0,5205	0,5195	0,5200	0,5255	0,5275	0,5310	0,5335	0,5365	0,5390	0,5405	0,5445	0,5515
Escherichia coli	C01	2	3	irradiated group	0,0055	0,0160	0,0315	0,0555	0,0895	0,1620	0,2415	0,3355	0,4285	0,4900	0,5075	0,5180	0,5210	0,5205	0,5195	0,5210	0,5225	0,5265	0,5300	0,5335	0,5365	0,5380	0,5405	0,5445	0,5515
Escherichia coli	C02	2	3	control group	0,0025	0,0140	0,0265	0,0485	0,0835	0,1450	0,2295	0,3135	0,4115	0,4820	0,5065	0,5190	0,5200	0,5205	0,5195	0,5210	0,5245	0,5275	0,5320	0,5345	0,5375	0,5400	0,5425	0,5465	0,5545
Escherichia coli	C03	2	4	irradiated group	0,0025	0,0130	0,0305	0,0515	0,0825	0,1600	0,2425	0,3345	0,4305	0,4900	0,5085	0,5180	0,5190	0,5185	0,5175	0,5180	0,5225	0,5245	0,5280	0,5325	0,5345	0,5380	0,5405	0,5445	0,5515
Escherichia coli	C04	2	4	control group	0,0065	0,0180	0,0345	0,0575	0,0855	0,1530	0,2365	0,3325	0,4225	0,4880	0,5085	0,5190	0,5200	0,5195	0,5185	0,5200	0,5245	0,5275	0,5300	0,5335	0,5355	0,5390	0,5405	0,5455	0,5525
Escherichia coli	A01	3	1	irradiated group	0,0100	0,0170	0,0320	0,0565	0,1005	0,1595	0,2450	0,3415	0,4405	0,5030	0,5305	0,5415	0,5440	0,5460	0,5455	0,5450	0,5470	0,5500	0,5540	0,5595	0,5640	0,5660	0,5695	0,5765	0,5795
Escherichia coli	A02	3	1	control group	0,0080	0,0140	0,0270	0,0485	0,0775	0,1545	0,2420	0,3425	0,4325	0,5080	0,5395	0,5525	0,5530	0,5530	0,5525	0,5530	0,5570	0,5590	0,5640	0,5685	0,5730	0,5780	0,5815	0,5875	0,5915
Escherichia coli	A03	3	2	irradiated group	0,0050	0,0140	0,0300	0,0555	0,0895	0,1605	0,2490	0,3475	0,4415	0,5110	0,5385	0,5495	0,5510	0,5510	0,5505	0,5490	0,5510	0,5520	0,5570	0,5625	0,5650	0,5690	0,5725	0,5775	0,5815
Escherichia coli	A04	3	2	control group	0,0060	0,0150	0,0290	0,0535	0,0805	0,1555	0,2450	0,3475	0,4445	0,5140	0,5435	0,5555	0,5560	0,5550	0,5535	0,5540	0,5570	0,5580	0,5630	0,5675	0,5710	0,5760	0,5785	0,5835	0,5865
Escherichia coli	C01	3	3	irradiated group	0,0070	0,0170	0,0330	0,0565	0,0905	0,1555	0,2510	0,3495	0,4435	0,5120	0,5395	0,5525	0,5550	0,5550	0,5545	0,5540	0,5560	0,5580	0,5620	0,5665	0,5710	0,5750	0,5775	0,5835	0,5865
Escherichia coli	C02	3	3	control group	0,0080	0,0140	0,0280	0,0535	0,0795	0,1575	0,2430	0,3445	0,4415	0,5120	0,5435	0,5565	0,5580	0,5570	0,5575	0,5570	0,5620	0,5630	0,5670	0,5715	0,5760	0,5800	0,5835	0,5895	0,5925
Escherichia coli	C03	3	4	irradiated group	0,0020	0,0130	0,0310	0,0585	0,0855	0,1675	0,2540	0,3545	0,4455	0,5130	0,5405	0,5515	0,5520	0,5520	0,5515	0,5520	0,5540	0,5560	0,5600	0,5655	0,5690	0,5740	0,5765	0,5825	0,5845
Escherichia coli	C04	3	4	control group	0,0060	0,0160	0,0320	0,0555	0,0905	0,1635	0,2540	0,3485	0,4505	0,5150	0,5445	0,5545	0,5550	0,5540	0,5545	0,5540	0,5570	0,5590	0,5630	0,5675	0,5720	0,5760	0,5795	0,5845	0,5875
Escherichia coli	A01	4	1	irradiated group	0,0080	0,0155	0,0300	0,0550	0,0980	0,1565	0,2520	0,3445	0,4420	0,5110	0,5390	0,5495	0,5525	0,5520	0,5525	0,5515	0,5530	0,5585	0,5610	0,5640	0,5660	0,5690	0,5715	0,5700	0,5720
Escherichia coli	A02	4	1	control group	0,0070	0,0135	0,0270	0,0530	0,0880	0,1605	0,2480	0,3605	0,4570	0,5270	0,5540	0,5615	0,5635	0,5620	0,5635	0,5665	0,5710	0,5775	0,5820	0,5860	0,5920	0,5970	0,6015	0,5950	0,5960
Escherichia coli	A03	4	2	irradiated group	0,0030	0,0125	0,0270	0,0510	0,0820	0,1615	0,2520	0,3455	0,4380	0,5160	0,5480	0,5585	0,5605	0,5590	0,5575	0,5565	0,5560	0,5605	0,5630	0,5660	0,5680	0,5700	0,5725	0,5760	0,5760
Escherichia coli	A04	4	2	control group	0,0050	0,0145	0,0310	0,0600	0,0960	0,1685	0,2650	0,3605	0,4630	0,5300	0,5590	0,5675	0,5675	0,5660	0,5655	0,5655	0,5700	0,5745	0,5770	0,5800	0,5830	0,5860	0,5885	0,5790	0,5810
Escherichia coli	C01	4	3	irradiated group	0,0050	0,0125	0,0300	0,0500	0,0860	0,1625	0,2380	0,3495	0,4420	0,5150	0,5470	0,5585	0,5615	0,5600	0,5595	0,5585	0,5590	0,5635	0,5660	0,5690	0,5710	0,5730	0,5745	0,5800	0,5840
Escherichia coli	C02	4	3	control group	0,0050	0,0135	0,0260	0,0490	0,0820	0,1595	0,2430	0,3475	0,4470	0,5220	0,5540	0,5665	0,5675	0,5660	0,5655	0,5665	0,5700	0,5745	0,5780	0,5800	0,5830	0,5860	0,5885	0,5880	0,5890
Escherichia coli	C03	4	4	irradiated group	0,0020	0,0115	0,0280	0,0490	0,0920	0,1585	0,2580	0,3615	0,4500	0,5210	0,5510	0,5615	0,5615	0,5610	0,5605	0,5615	0,5630	0,5685	0,5710	0,5740	0,5770	0,5790	0,5825	0,5790	0,5820
Escherichia coli	C04	4	4	control group	0,0070	0,0165	0,0320	0,0570	0,0990	0,1735	0,2610	0,3685	0,4600	0,5260	0,5540	0,5635	0,5625	0,5620	0,5615	0,5625	0,5660	0,5705	0,5740	0,5760	0,5790	0,5810	0,5835	0,5870	0,5880
Escherichia coli	A01	5	1	irradiated group	0,0115	0,0155	0,0295	0,0555	0,0905	0,1680	0,2595	0,3615	0,4535	0,5155	0,5395	0,5505	0,5520	0,5520	0,5515	0,5510	0,5535	0,5570	0,5625	0,5655	0,5675	0,5675	0,5670	0,5685	0,5690
Escherichia coli	A02	5	1	control group	0,0115	0,0155	0,0305	0,0585	0,1115	0,2070	0,3025	0,4005	0,4925	0,5515	0,5725	0,5775	0,5780	0,5800	0,5835	0,5890	0,5945	0,6010	0,6095	0,6165	0,6235	0,6285	0,6290	0,6295	0,6300
Escherichia coli	A03	5	2	irradiated group	0,0055	0,0125	0,0275	0,0505	0,0835	0,1650	0,2495	0,3535	0,4455	0,5205	0,5485	0,5585	0,5590	0,5580	0,5565	0,5550	0,5555	0,5590	0,5645	0,5665	0,5675	0,5685	0,5670	0,5695	0,5700
Escherichia coli	A04	5	2	control group	0,0075	0,0135	0,0305	0,0595	0,1075	0,1880	0,2715	0,3815	0,4725	0,5375	0,5625	0,5705	0,5700	0,5690	0,5705	0,5720	0,5765	0,5810	0,5885	0,5925	0,5965	0,5985	0,5990	0,5985	0,5990
Escherichia coli	C01	5	3	irradiated group	0,0085	0,0125	0,0285	0,0535	0,0875	0,1650	0,2575	0,3555	0,4485	0,5185	0,5465	0,5575	0,5590	0,5580	0,5575	0,5550	0,5565	0,5600	0,5655	0,5685	0,5695	0,5705	0,5680	0,5675	0,5680
Escherichia coli	C02	5	3	control group	0,0045	0,0105	0,0275	0,0525	0,0945	0,1760	0,2625	0,3745	0,4615	0,5325	0,5595	0,5695	0,5690	0,5680	0,5685	0,5700	0,5745	0,5780	0,5845	0,5865	0,5895	0,5905	0,5890	0,5905	0,5910
Escherichia coli	C03	5	4	irradiated group	0,0015	0,0095	0,0285	0,0575	0,0965	0,1760	0,2615	0,3655	0,4645	0,5265	0,5535	0,5625	0,5620	0,5610	0,5595	0,5600	0,5635	0,5670	0,5725	0,5755	0,5785	0,5795	0,5780	0,5795	0,5800
Escherichia coli	C04	5	4	control group	0,0075	0,0145	0,0335	0,0655	0,1055	0,1860	0,2785	0,3815	0,4745	0,5345	0,5595	0,5675	0,5660	0,5650	0,5645	0,5660	0,5695	0,5740	0,5795	0,5815	0,5835	0,5835	0,5830	0,5845	0,5850
Escherichia coli	A01	6	1	irradiated group	0,0105	0,0160	0,0305	0,0555	0,0935	0,1690	0,2530	0,3475	0,4350	0,4960	0,5205	0,5285	0,5310	0,5315	0,5280	0,5270	0,5295	0,5360	0,5400	0,5425	0,5445	0,5510	0,5530	0,5540	0,5560
Escherichia coli	A02	6	1	control group	0,0125	0,0180	0,0325	0,0625	0,1195	0,2120	0,3150	0,4145	0,4970	0,5400	0,5555	0,5585	0,5590	0,5605	0,5600	0,5660	0,5745	0,5830	0,5890	0,5955	0,6035	0,6140	0,6210	0,6230	0,6250
Escherichia coli	A03	6	2	irradiated group	0,0055	0,0130	0,0265	0,0515	0,0815	0,1590	0,2440	0,3415	0,4320	0,5000	0,5245	0,5335	0,5350	0,5345	0,5290	0,5280	0,5305	0,5360	0,5380	0,5405	0,5425	0,5480	0,5490	0,5500	0,5490
Escherichia coli	A04	6	2	control group	0,0075	0,0140	0,0305	0,0605	0,1145	0,2030	0,2900	0,3875	0,4770	0,5280	0,5475	0,5515	0,5500	0,5515	0,5490	0,5540	0,5605	0,5690	0,5740	0,5795	0,5865	0,5970	0,6040	0,6080	0,6100
Escherichia coli	C01	6	3	irradiated group	0,0085	0,0150	0,0285	0,0555	0,0895	0,1680	0,2520	0,3365	0,4400	0,5010	0,5265	0,5355	0,5370	0,5365	0,5330	0,5310	0,5335	0,5380	0,5410	0,5445	0,5455	0,5510	0,5520	0,5520	0,5520
Escherichia coli	C02	6	3	control group	0,0035	0,0110	0,0255	0,0525	0,0945	0,1690	0,2590	0,3605	0,4560	0,5130	0,5385	0,5475	0,5460	0,5475	0,5450	0,5480	0,5545	0,5610	0,5650	0,5685	0,5705	0,5790	0,5840	0,5860	0,5870
Escherichia coli	C03	6	4	irradiated group	0,0055	0,0130	0,0275	0,0535	0,0955	0,1820	0,2640	0,3595	0,4570	0,5130	0,5365	0,5425	0,5420	0,5425	0,5380	0,5390	0,5435	0,5510	0,5540	0,5585	0,5585	0,5650	0,5690	0,5710	0,5720
Escherichia coli	C04	6	4	control group	0,0055	0,0150	0,0285	0,0575	0,0985	0,1850	0,2650	0,3615	0,4510	0,5150	0,5385	0,5445	0,5440	0,5435	0,5400	0,5410	0,5465	0,5530	0,5560	0,5595	0,5585	0,5650	0,5670	0,5700	0,5710
Escherichia coli	A01	0	5	irradiated group	0,0175	0,0220	0,0340	0,0565	0,0870	0,1620	0,2660	0,3565	0,4355	0,4945	0,5240	0,5315	0,5335	0,5320	0,5320	0,5340	0,5385	0,5425	0,5470	0,5495	0,5520	0,5570	0,5615	0,5645	0,5685
Escherichia coli	A02	0	5	control group	0,0095	0,0160	0,0280	0,0515	0,0860	0,1600	0,2620	0,3525	0,4365	0,4945	0,5250	0,5335	0,5325	0,5310	0,5300	0,5330	0,5365	0,5405	0,5450	0,5485	0,5530	0,5570	0,5615	0,5645	0,5685
Escherichia coli	A03	0	6	irradiated group	0,0025	0,0120	0,0250	0,0485	0,0810	0,1560	0,2560	0,3535	0,4335	0,4895	0,5180	0,5265	0,5235	0,5220	0,5220	0,5250	0,5295	0,5335	0,5380	0,5405	0,5460	0,5500	0,5545	0,5575	0,5615
Escherichia coli	A04	0	6	control group	0,0025	0,0130	0,0290	0,0555	0,0890	0,1720	0,2720	0,3625	0,4425	0,4945	0,5210	0,5275	0,5255	0,5240	0,5240	0,5270	0,5305	0,5345	0,5390	0,5425	0,5460	0,5510	0,5555	0,5585	0,5615
Escherichia coli	C01	0	7	irradiated group	0,0075	0,0180	0,0320	0,0555	0,0870	0,1630	0,2670	0,3555	0,4385	0,4955	0,5220	0,5305	0,5305	0,5290	0,5300	0,5320	0,5365	0,5405	0,5440	0,5475	0,5510	0,5550	0,5615	0,5645	0,5685
Escherichia coli	C02	0	7	control group	0,0035	0,0130	0,0280	0,0525	0,0860	0,1620	0,2640	0,3545	0,4345	0,4925	0,5210	0,5275	0,5265	0,5250	0,5250	0,5280	0,5325	0,5365	0,5410	0,5445	0,5480	0,5530	0,5575	0,5615	0,5655
Escherichia coli	C03	0	8	irradiated group	0,0055	0,0180	0,0370	0,0685	0,1050	0,2040	0,2990	0,3905	0,4625	0,5085	0,5280	0,5305	0,5285	0,5270	0,5280	0,5330	0,5365	0,5395	0,5450	0,5475	0,5510	0,5560	0,5605	0,5645	0,5685
Escherichia coli	C04	0	8	control group	0,0025	0,0150	0,0340	0,0645	0,0980	0,1910	0,2870	0,3825	0,4535	0,5035	0,5250	0,5285	0,5265	0,5250	0,5250	0,5290	0,5335	0,5375	0,5420	0,5445	0,5480	0,5520	0,5575	0,5605	0,5635
Escherichia coli	A01	1	5	irradiated group	0,0135	0,0215	0,0335	0,0490	0,0750	0,1485	0,2550	0,3375	0,4275	0,4895	0,5185	0,5270	0,5295	0,5300	0,5295	0,5295	0,5325	0,5350	0,5395	0,5420	0,5435	0,5465	0,5500	0,5515	0,5545
Escherichia coli	A02	1	5	control group	0,0115	0,0175	0,0295	0,0470	0,0670	0,1505	0,2410	0,3465	0,4375	0,4955	0,5255	0,5350	0,5355	0,5340	0,5345	0,5345	0,5385	0,5410	0,5445	0,5470	0,5505	0,5525	0,5560	0,5575	0,5605
Escherichia coli	A03	1	6	irradiated group	0,0035	0,0135	0,0285	0,0480	0,0740	0,1515	0,2460	0,3475	0,4365	0,4955	0,5225	0,5310	0,5315	0,5310	0,5295	0,5285	0,5315	0,5340	0,5375	0,5400	0,5425	0,5445	0,5480	0,5505	0,5525
Escherichia coli	A04	1	6	control group	0,0045	0,0155	0,0315	0,0490	0,0710	0,1525	0,2460	0,3485	0,4295	0,4875	0,5145	0,5230	0,5215	0,5200	0,5195	0,5215	0,5255	0,5280	0,5325	0,5350	0,5375	0,5395	0,5430	0,5465	0,5485
Escherichia coli	C01	1	7	irradiated group	0,0095	0,0185	0,0335	0,0470	0,0790	0,1515	0,2530	0,3505	0,4385	0,4995	0,5265	0,5360	0,5375	0,5370	0,5365	0,5365	0,5385	0,5410	0,5455	0,5480	0,5495	0,5515	0,5560	0,5575	0,5605
Escherichia coli	C02	1	7	control group	0,0045	0,0135	0,0285	0,0480	0,0650	0,1395	0,2490	0,3415	0,4285	0,4915	0,5215	0,5310	0,5305	0,5300	0,5305	0,5305	0,5355	0,5380	0,5415	0,5450	0,5475	0,5495	0,5530	0,5555	0,5585
Escherichia coli	C03	1	8	irradiated group	0,0015	0,0135	0,0285	0,0420	0,0690	0,1485	0,2520	0,3525	0,4365	0,4965	0,5235	0,5320	0,5305	0,5300	0,5305	0,5305	0,5345	0,5360	0,5405	0,5430	0,5455	0,5475	0,5510	0,5535	0,5555
Escherichia coli	C04	1	8	control group	0,0035	0,0145	0,0335	0,0530	0,0700	0,1565	0,2550	0,3565	0,4365	0,4955	0,5215	0,5300	0,5275	0,5270	0,5275	0,5285	0,5335	0,5350	0,5395	0,5420	0,5445	0,5465	0,5500	0,5525	0,5545
Escherichia coli	A01	2	5	irradiated group	0,0160	0,0220	0,0350	0,0520	0,0735	0,1600	0,2505	0,3480	0,4300	0,4885	0,5140	0,5215	0,5230	0,5225	0,5220	0,5245	0,5250	0,5315	0,5365	0,5395	0,5425	0,5465	0,5495	0,5535	0,5585
Escherichia coli	A02	2	5	control group	0,0080	0,0150	0,0290	0,0460	0,0635	0,1520	0,2435	0,3450	0,4310	0,4885	0,5140	0,5245	0,5230	0,5215	0,5220	0,5245	0,5280	0,5345	0,5395	0,5425	0,5465	0,5495	0,5545	0,5595	0,5635
Escherichia coli	A03	2	6	irradiated group	0,0050	0,0170	0,0310	0,0510	0,0745	0,1560	0,2535	0,3460	0,4300	0,4865	0,5100	0,5175	0,5180	0,5165	0,5150	0,5155	0,5180	0,5235	0,5285	0,5325	0,5355	0,5385	0,5425	0,5465	0,5505

Escherichia coli	A04	2	6	control group	0,0050	0,0160	0,0330	0,0510	0,0775	0,1510	0,2485	0,3520	0,4320	0,4895	0,5130	0,5215	0,5180	0,5175	0,5170	0,5195	0,5230	0,5295	0,5345	0,5375	0,5405	0,5435	0,5485	0,5525	0,5555
Escherichia coli	C01	2	7	irradiated group	0,0130	0,0230	0,0340	0,0550	0,0905	0,1620	0,2545	0,3480	0,4360	0,4915	0,5150	0,5245	0,5240	0,5235	0,5230	0,5245	0,5260	0,5315	0,5365	0,5405	0,5435	0,5465	0,5505	0,5555	0,5585
Escherichia coli	C02	2	7	control group	0,0070	0,0160	0,0290	0,0520	0,0755	0,1480	0,2535	0,3480	0,4310	0,4885	0,5140	0,5235	0,5210	0,5205	0,5200	0,5235	0,5270	0,5335	0,5385	0,5415	0,5455	0,5505	0,5545	0,5595	0,5645
Escherichia coli	C03	2	8	irradiated group	0,0050	0,0180	0,0340	0,0530	0,0785	0,1670	0,2625	0,3590	0,4400	0,4955	0,5190	0,5285	0,5270	0,5265	0,5260	0,5275	0,5310	0,5375	0,5415	0,5445	0,5475	0,5515	0,5545	0,5585	0,5615
Escherichia coli	C04	2	8	control group	0,0050	0,0170	0,0340	0,0520	0,0835	0,1670	0,2615	0,3540	0,4350	0,4915	0,5150	0,5235	0,5210	0,5195	0,5200	0,5225	0,5260	0,5325	0,5375	0,5405	0,5435	0,5465	0,5505	0,5555	0,5595
Escherichia coli	A01	3	5	irradiated group	0,0055	0,0105	0,0255	0,0430	0,0780	0,1495	0,2450	0,3440	0,4245	0,4840	0,5095	0,5180	0,5205	0,5220	0,5210	0,5200	0,5225	0,5265	0,5310	0,5330	0,5360	0,5390	0,5425	0,5425	0,5435
Escherichia coli	A02	3	5	control group	0,0035	0,0095	0,0255	0,0450	0,0820	0,1565	0,2630	0,3600	0,4455	0,5010	0,5255	0,5320	0,5325	0,5320	0,5330	0,5340	0,5395	0,5435	0,5480	0,5510	0,5550	0,5610	0,5655	0,5675	0,5705
Escherichia coli	A03	3	6	irradiated group	-0,0035	0,0065	0,0215	0,0390	0,0700	0,1465	0,2390	0,3410	0,4215	0,4760	0,5015	0,5100	0,5105	0,5090	0,5090	0,5080	0,5115	0,5145	0,5180	0,5210	0,5240	0,5280	0,5305	0,5315	0,5325
Escherichia coli	A04	3	6	control group	-0,0015	0,0075	0,0255	0,0480	0,0750	0,1585	0,2510	0,3560	0,4315	0,4850	0,5105	0,5170	0,5155	0,5150	0,5160	0,5170	0,5225	0,5265	0,5310	0,5340	0,5390	0,5450	0,5485	0,5515	0,5545
Escherichia coli	C01	3	7	irradiated group	0,0025	0,0105	0,0255	0,0480	0,0820	0,1515	0,2480	0,3490	0,4285	0,4870	0,5105	0,5200	0,5215	0,5210	0,5210	0,5210	0,5225	0,5265	0,5300	0,5330	0,5350	0,5400	0,5415	0,5425	0,5435
Escherichia coli	C02	3	7	control group	-0,0005	0,0095	0,0245	0,0470	0,0820	0,1755	0,2730	0,3740	0,4505	0,5010	0,5205	0,5270	0,5255	0,5260	0,5270	0,5290	0,5345	0,5395	0,5430	0,5470	0,5520	0,5570	0,5615	0,5635	0,5675
Escherichia coli	C03	3	8	irradiated group	-0,0015	0,0095	0,0265	0,0400	0,0910	0,1685	0,2520	0,3530	0,4345	0,4900	0,5125	0,5210	0,5205	0,5190	0,5190	0,5190	0,5215	0,5255	0,5290	0,5320	0,5350	0,5400	0,5425	0,5435	0,5445
Escherichia coli	C04	3	8	control group	0,0015	0,0125	0,0315	0,0530	0,0850	0,1725	0,2660	0,3650	0,4445	0,4970	0,5185	0,5260	0,5245	0,5240	0,5240	0,5250	0,5295	0,5335	0,5380	0,5400	0,5450	0,5500	0,5535	0,5555	0,5575
Escherichia coli	A01	4	5	irradiated group	0,0005	0,0105	0,0275	0,0450	0,0810	0,1605	0,2470	0,3440	0,4175	0,4720	0,4935	0,5010	0,5045	0,5050	0,5050	0,5070	0,5115	0,5165	0,5220	0,5240	0,5300	0,5330	0,5375	0,5425	0,5465
Escherichia coli	A02	4	5	control group	-0,0015	0,0085	0,0235	0,0510	0,1010	0,1945	0,2940	0,3930	0,4605	0,5030	0,5175	0,5220	0,5225	0,5240	0,5280	0,5320	0,5405	0,5455	0,5510	0,5560	0,5620	0,5690	0,5755	0,5825	0,5885
Escherichia coli	A03	4	6	irradiated group	-0,0095	0,0015	0,0175	0,0390	0,0700	0,1515	0,2400	0,3360	0,4175	0,4720	0,4965	0,5030	0,5045	0,5040	0,5050	0,5040	0,5085	0,5135	0,5170	0,5210	0,5260	0,5290	0,5345	0,5375	0,5425
Escherichia coli	A04	4	6	control group	-0,0055	0,0055	0,0245	0,0530	0,0900	0,1815	0,2810	0,3850	0,4575	0,5020	0,5205	0,5240	0,5245	0,5260	0,5300	0,5350	0,5435	0,5485	0,5550	0,5600	0,5670	0,5720	0,5785	0,5845	0,5915
Escherichia coli	C01	4	7	irradiated group	-0,0045	0,0075	0,0235	0,0440	0,0770	0,1605	0,2470	0,3420	0,4215	0,4760	0,4995	0,5080	0,5095	0,5100	0,5110	0,5110	0,5155	0,5205	0,5260	0,5280	0,5330	0,5370	0,5415	0,5465	0,5505
Escherichia coli	C02	4	7	control group	-0,0065	0,0055	0,0205	0,0490	0,0990	0,1975	0,2990	0,3990	0,4675	0,5090	0,5255	0,5300	0,5305	0,5320	0,5370	0,5420	0,5505	0,5575	0,5630	0,5690	0,5760	0,5830	0,5895	0,5965	0,6025
Escherichia coli	C03	4	8	irradiated group	-0,0105	0,0025	0,0205	0,0430	0,0730	0,1675	0,2580	0,3570	0,4305	0,4820	0,5025	0,5090	0,5085	0,5090	0,5110	0,5130	0,5195	0,5255	0,5300	0,5340	0,5400	0,5450	0,5515	0,5575	0,5635
Escherichia coli	C04	4	8	control group	-0,0055	0,0075	0,0245	0,0520	0,1020	0,1925	0,2920	0,3970	0,4645	0,5060	0,5225	0,5270	0,5265	0,5270	0,5300	0,5340	0,5405	0,5465	0,5510	0,5560	0,5610	0,5670	0,5735	0,5785	0,5845
Escherichia coli	A01	5	5	irradiated group	0,0090	0,0185	0,0330	0,0600	0,0910	0,1705	0,2650	0,3650	0,4410	0,4925	0,5165	0,5220	0,5240	0,5240	0,5240	0,5255	0,5280	0,5315	0,5360	0,5385	0,5425	0,5465	0,5510	0,5535	0,5585
Escherichia coli	A02	5	5	control group	0,0100	0,0175	0,0330	0,0620	0,1120	0,2035	0,3030	0,4030	0,4740	0,5145	0,5305	0,5340	0,5350	0,5360	0,5390	0,5465	0,5510	0,5575	0,5630	0,5675	0,5755	0,5805	0,5870	0,5925	0,5985
Escherichia coli	A03	5	6	irradiated group	0,0010	0,0145	0,0310	0,0510	0,0870	0,1745	0,2610	0,3670	0,4390	0,4895	0,5075	0,5120	0,5130	0,5110	0,5110	0,5125	0,5150	0,5205	0,5240	0,5265	0,5315	0,5355	0,5400	0,5425	0,5475
Escherichia coli	A04	5	6	control group	0,0060	0,0185	0,0370	0,0680	0,1200	0,2095	0,3160	0,4110	0,4780	0,5145	0,5275	0,5290	0,5300	0,5300	0,5340	0,5405	0,5460	0,5525	0,5570	0,5615	0,5675	0,5735	0,5800	0,5845	0,5915
Escherichia coli	C01	5	7	irradiated group	0,0110	0,0205	0,0360	0,0600	0,0870	0,1835	0,2690	0,3730	0,4490	0,4995	0,5195	0,5240	0,5260	0,5250	0,5250	0,5265	0,5280	0,5335	0,5370	0,5395	0,5435	0,5475	0,5520	0,5545	0,5595
Escherichia coli	C02	5	7	control group	0,0090	0,0185	0,0350	0,0650	0,1180	0,2105	0,3180	0,4090	0,4770	0,5165	0,5315	0,5350	0,5360	0,5360	0,5410	0,5475	0,5520	0,5595	0,5640	0,5685	0,5755	0,5815	0,5880	0,5925	0,5995
Escherichia coli	C03	5	8	irradiated group	0,0050	0,0165	0,0330	0,0520	0,0940	0,1895	0,2690	0,3760	0,4520	0,4985	0,5185	0,5230	0,5230	0,5220	0,5210	0,5235	0,5270	0,5315	0,5360	0,5385	0,5435	0,5475	0,5520	0,5565	0,5615
Escherichia coli	C04	5	8	control group	0,0090	0,0225	0,0420	0,0740	0,1270	0,2185	0,3300	0,4230	0,4850	0,5205	0,5335	0,5350	0,5350	0,5360	0,5400	0,5465	0,5510	0,5585	0,5620	0,5665	0,5735	0,5785	0,5850	0,5895	0,5955
Escherichia coli	A01	6	5	irradiated group	0,0135	0,0220	0,0370	0,0630	0,1025	0,1835	0,2765	0,3775	0,4525	0,5005	0,5210	0,5270	0,5285	0,5285	0,5295	0,5315	0,5350	0,5390	0,5435	0,5460	0,5510	0,5545	0,5575	0,5595	0,5645
Escherichia coli	A02	6	5	control group	0,0115	0,0160	0,0320	0,0610	0,1115	0,2055	0,3045	0,4035	0,4715	0,5115	0,5260	0,5300	0,5315	0,5315	0,5365	0,5435	0,5510	0,5560	0,5625	0,5670	0,5740	0,5795	0,5845	0,5885	0,5945
Escherichia coli	A03	6	6	irradiated group	0,0015	0,0110	0,0260	0,0520	0,0905	0,1685	0,2595	0,3585	0,4345	0,4865	0,5060	0,5130	0,5125	0,5115	0,5115	0,5125	0,5160	0,5200	0,5255	0,5280	0,5320	0,5365	0,5395	0,5415	0,5465
Escherichia coli	A04	6	6	control group	0,0045	0,0140	0,0320	0,0630	0,1155	0,2065	0,3095	0,4115	0,4745	0,5125	0,5260	0,5290	0,5295	0,5295	0,5335	0,5405	0,5470	0,5530	0,5585	0,5640	0,5700	0,5755	0,5795	0,5835	0,5895
Escherichia coli	C01	6	7	irradiated group	0,0065	0,0160	0,0330	0,0580	0,1075	0,1885	0,2775	0,3825	0,4545	0,5035	0,5220	0,5280	0,5295	0,5285	0,5295	0,5315	0,5350	0,5390	0,5435	0,5460	0,5510	0,5545	0,5575	0,5605	0,5645
Escherichia coli	C02	6	7	control group	0,0055	0,0150	0,0320	0,0630	0,1175	0,2085	0,3125	0,4135	0,4805	0,5175	0,5320	0,5350	0,5355	0,5375	0,5415	0,5485	0,5550	0,5620	0,5675	0,5730	0,5790	0,5855	0,5895	0,5935	0,5995
Escherichia coli	C03	6	8	irradiated group	0,0015	0,0130	0,0320	0,0590	0,1045	0,1995	0,2935	0,3985	0,4655	0,5075	0,5240	0,5280	0,5275	0,5265	0,5275	0,5305	0,5350	0,5390	0,5445	0,5490	0,5540	0,5575	0,5615	0,5645	0,5705
Escherichia coli	C04	6	8	control group	0,0045	0,0150	0,0360	0,0670	0,1225	0,2145	0,3215	0,4175	0,4805	0,5155	0,5290	0,5320	0,5315	0,5325	0,5375	0,5435	0,5510	0,5560	0,5625	0,5670	0,5730	0,5785	0,5835	0,5875	0,5925
				**Messzeitpunkt**	**0 h**	**0 h 30 min**	**1 h**	**1 h 30 min**	**2 h**	**2 h 30 min**	**3 h**	**3 h 30 min**	**4 h**	**4 h 30 min**	**5 h**	**5 h 30 min**	**6 h**	**6 h 30 min**	**7 h**	**7 h 30 min**	**8 h**	**8 h 30 min**	**9 h**	**9 h 30 min**	**10 h**	**10 h 30 min**	**11 h**	**11 h 30 min**	**12 h**
Erreger	Position - 12-Well-Mikro-titerplatte	Stage	Stichproben-anzahl	Versuchsgruppe	Blank corrected based on Raw Data (600)	Blank corrected based on Raw Data (600)	Blank corrected based on Raw Data (600)	Blank corrected based on Raw Data (600)	Blank corrected based on Raw Data (600)	Blank corrected based on Raw Data (600)	Blank corrected based on Raw Data (600)	Blank corrected based on Raw Data (600)	Blank corrected based on Raw Data (600)	Blank corrected based on Raw Data (600)	Blank corrected based on Raw Data (600)	Blank corrected based on Raw Data (600)	Blank corrected based on Raw Data (600)	Blank corrected based on Raw Data (600)	Blank corrected based on Raw Data (600)	Blank corrected based on Raw Data (600)	Blank corrected based on Raw Data (600)	Blank corrected based on Raw Data (600)	Blank corrected based on Raw Data (600)	Blank corrected based on Raw Data (600)	Blank corrected based on Raw Data (600)	Blank corrected based on Raw Data (600)	Blank corrected based on Raw Data (600)	Blank corrected based on Raw Data (600)	Blank corrected based on Raw Data (600)
MRSA	A01	0	1	irradiated group	0,0150	0,0150	0,0200	0,0320	0,0550	0,0925	0,1330	0,1775	0,2290	0,2930	0,3545	0,4015	0,4405	0,4660	0,4860	0,4975	0,5080	0,5150	0,5210	0,5250	0,5275	0,5290	0,5290	0,5255	0,5220
MRSA	A02	0	1	control group	0,0100	0,0120	0,0190	0,0300	0,0540	0,0915	0,1350	0,1795	0,2310	0,2930	0,3545	0,4015	0,4405	0,4690	0,4900	0,5015	0,5120	0,5190	0,5250	0,5290	0,5305	0,5330	0,5320	0,5295	0,5270
MRSA	A03	0	2	irradiated group	0,0060	0,0110	0,0170	0,0300	0,0550	0,0925	0,1350	0,1805	0,2330	0,2950	0,3545	0,4035	0,4405	0,4680	0,4880	0,5005	0,5100	0,5180	0,5230	0,5270	0,5295	0,5320	0,5300	0,5275	0,5250
MRSA	A04	0	2	control group	0,0060	0,0130	0,0210	0,0360	0,0610	0,1005	0,1450	0,1905	0,2420	0,3050	0,3625	0,4085	0,4445	0,4700	0,4880	0,5015	0,5100	0,5170	0,5230	0,5280	0,5295	0,5310	0,5300	0,5275	0,5240
MRSA	C01	0	3	irradiated group	0,0110	0,0140	0,0210	0,0330	0,0570	0,0955	0,1390	0,1825	0,2360	0,3010	0,3585	0,4045	0,4425	0,4670	0,4870	0,4985	0,5080	0,5160	0,5220	0,5260	0,5295	0,5320	0,5320	0,5295	0,5270
MRSA	C02	0	3	control group	0,0060	0,0090	0,0160	0,0300	0,0550	0,0935	0,1380	0,1845	0,2390	0,3030	0,3595	0,4065	0,4445	0,4690	0,4880	0,5005	0,5100	0,5170	0,5230	0,5280	0,5305	0,5330	0,5320	0,5295	0,5280
MRSA	C03	0	4	irradiated group	0,0020	0,0070	0,0170	0,0310	0,0570	0,0955	0,1400	0,1865	0,2410	0,3040	0,3605	0,4075	0,4445	0,4690	0,4880	0,5005	0,5100	0,5170	0,5220	0,5270	0,5295	0,5310	0,5300	0,5275	0,5250
MRSA	C04	0	4	control group	0,0020	0,0110	0,0210	0,0390	0,0650	0,1065	0,1500	0,1975	0,2540	0,3200	0,3725	0,4155	0,4495	0,4730	0,4890	0,5015	0,5100	0,5170	0,5220	0,5260	0,5285	0,5300	0,5290	0,5265	0,5230
MRSA	A01	1	1	irradiated group	0,0105	0,0125	0,0175	0,0270	0,0410	0,0640	0,0965	0,1390	0,1875	0,2540	0,3205	0,3810	0,4310	0,4760	0,5120	0,5385	0,5570	0,5725	0,5870	0,5955	0,6025	0,6095	0,6130	0,6140	0,6150
MRSA	A02	1	1	control group	0,0075	0,0095	0,0135	0,0240	0,0400	0,0700	0,1145	0,1660	0,2195	0,2860	0,3575	0,4160	0,4670	0,5100	0,5400	0,5595	0,5770	0,5925	0,6060	0,6145	0,6185	0,6255	0,6290	0,6280	0,6270
MRSA	A03	1	2	irradiated group	0,0065	0,0105	0,0155	0,0240	0,0350	0,0520	0,0785	0,1180	0,1645	0,2160	0,2795	0,3480	0,4060	0,4570	0,5000	0,5305	0,5540	0,5715	0,5870	0,5985	0,6055	0,6155	0,6200	0,6240	0,6270
MRSA	A04	1	2	control group	0,0095	0,0135	0,0185	0,0310	0,0490	0,0820	0,1285	0,1790	0,2325	0,3010	0,3675	0,4250	0,4750	0,5140	0,5430	0,5615	0,5780	0,5925	0,6060	0,6155	0,6195	0,6275	0,6300	0,6300	0,6290
MRSA	C01	1	3	irradiated group	0,0095	0,0125	0,0165	0,0270	0,0370	0,0560	0,0845	0,1250	0,1715	0,2260	0,2945	0,3600	0,4130	0,4600	0,5000	0,5305	0,5530	0,5705	0,5850	0,5975	0,6045	0,6135	0,6200	0,6230	0,6250
MRSA	C02	1	3	control group	0,0065	0,0075	0,0125	0,0230	0,0390	0,0690	0,1135	0,1630	0,2165	0,2860	0,3555	0,4130	0,4640	0,5040	0,5350	0,5565	0,5730	0,5885	0,6020	0,6105	0,6165	0,6245	0,6260	0,6270	0,6260
MRSA	C03	1	4	irradiated group	0,0055	0,0115	0,0145	0,0260	0,0380	0,0570	0,0875	0,1310	0,1765	0,2340	0,3015	0,3680	0,4200	0,4660	0,5060	0,5345	0,5550	0,5715	0,5870	0,5985	0,6045	0,6135	0,6180	0,6200	0,6210
MRSA	C04	1	4	control group	0,0095	0,0145	0,0195	0,0310	0,0490	0,0820	0,1305	0,1790	0,2335	0,3020	0,3695	0,4260	0,4750	0,5130	0,5420	0,5615	0,5780	0,5925	0,6060	0,6135	0,6195	0,6265	0,6280	0,6280	0,6270
MRSA	A01	2	1	irradiated group	0,0115	0,0170	0,0205	0,0290	0,0415	0,0650	0,0950	0,1375	0,1865	0,2460	0,3200	0,3815	0,4305	0,4755	0,5115	0,5360	0,5555	0,5705	0,5815	0,5900	0,5980	0,6030	0,6035	0,6035	0,6015
MRSA	A02	2	1	control group	0,0085	0,0110	0,0155	0,0260	0,0415	0,0710	0,1140	0,1635	0,2165	0,2850	0,3570	0,4145	0,4655	0,5055	0,5375	0,5570	0,5735	0,5875	0,5995	0,6090	0,6150	0,6200	0,6175	0,6165	0,6135
MRSA	A03	2	2	irradiated group	0,0085	0,0170	0,0205	0,0300	0,0415	0,0620	0,0920	0,1365	0,1845	0,2380	0,3080	0,3745	0,4305	0,4795	0,5175	0,5450	0,5645	0,5795	0,5905	0,6010	0,6080	0,6140	0,6155	0,6165	0,6175
MRSA	A04	2	2	control group	0,0065	0,0130	0,0195	0,0300	0,0465	0,0790	0,1250	0,1735	0,2295	0,2970	0,3650	0,4215	0,4695	0,5085	0,5385	0,5570	0,5735	0,5875	0,5995	0,6080	0,6150	0,6190	0,6185	0,6165	0,6145
MRSA	C01	2	3	irradiated group	0,0125	0,0180	0,0235	0,0330	0,0445	0,0680	0,0990	0,1425	0,1925	0,2520	0,3220	0,3835	0,4345	0,4795	0,5175	0,5440	0,5635	0,5775	0,5895	0,6000	0,6070	0,6130	0,6135	0,6145	0,6155
MRSA	C02	2	3	control group	0,0065	0,0120	0,0155	0,0260	0,0425	0,0750	0,1190	0,1685	0,2235	0,2930	0,3640	0,4195	0,4685	0,5095	0,5385	0,5570	0,5755	0,5885	0,5995	0,6080	0,6150	0,6190	0,6185	0,6165	0,6145
MRSA	C03	2	4	irradiated group	0,0095	0,0160	0,0225	0,0290	0,0445	0,0670	0,1030	0,1485	0,1975	0,2560	0,3290	0,3895	0,4405	0,4865	0,5215	0,5460	0,5645	0,5795	0,5905	0,6000	0,6070	0,6120	0,6115	0,6115	0,6105
MRSA	C04	2	4	control group	0,0065	0,0140	0,0185	0,0290	0,0485	0,0820	0,1310	0,1785	0,2345	0,3030	0,3690	0,4255	0,4725	0,5105	0,5385	0,5570	0,5735	0,5885	0,5985	0,6070	0,6140	0,6170	0,6165	0,6145	0,6125
MRSA	A01	3	1	irradiated group	0,0140	0,0175	0,0210	0,0295	0,0440	0,0665	0,0985	0,1420	0,1895	0,2510	0,3230	0,3865	0,4440	0,4860	0,5200	0,5475	0,5650	0,5770	0,5895	0,5985	0,6080	0,6135	0,6165	0,6210	0,6215
MRSA	A02	3	1	control group	0,0090	0,0115	0,0160	0,0255	0,0430	0,0705	0,1135	0,1620	0,2155	0,2830	0,3530	0,4125	0,4650	0,5060	0,5360	0,5575	0,5730	0,5860	0,5985	0,6095	0,6180	0,6205	0,6225	0,6240	0,6215
MRSA	A03	3	2	irradiated group	0,0090	0,0145	0,0200	0,0285	0,0410	0,0615	0,0925	0,1380	0,1845	0,2400	0,3080	0,3755	0,4360	0,4870	0,5240	0,5515	0,5710	0,5840	0,5955	0,6065	0,6160	0,6215	0,6255	0,6310	0,6315
MRSA	A04	3	2	control group	0,0080	0,0135	0,0200	0,0305	0,0500	0,0815	0,1285	0,1780	0,2325	0,3010	0,3670	0,4235	0,4720	0,5090	0,5370	0,5565	0,5720	0,5850	0,5975	0,6085	0,6160	0,6175	0,6205	0,6210	0,6205
MRSA	C01	3	3	irradiated group	0,0130	0,0175	0,0230	0,0315	0,0450	0,0675	0,1005	0,1460	0,1945	0,2540	0,3250	0,3885	0,4440	0,4910	0,5270	0,5535	0,5710	0,5840	0,5965	0,6065	0,6170	0,6215	0,6255	0,6310	0,6325
MRSA	C02	3	3	control group	0,0080	0,0115	0,0170	0,0275	0,0440	0,0755	0,1195	0,1680	0,2225	0,2930	0,3630	0,4195	0,4700	0,5090	0,5380	0,5595	0,5750	0,5880	0,6005	0,6105	0,6180	0,6215	0,6235	0,6260	0,6245
MRSA	C03	3	4	irradiated group	0,0070	0,0135	0,0190	0,0275	0,0430	0,0665	0,1025	0,1490	0,1975	0,2590	0,3290	0,3915	0,4480	0,4960	0,5300	0,5555	0,5730	0,5850	0,5985	0,6085	0,6170	0,6225	0,6265	0,6300	0,6305
MRSA	C04	3	4	control group	0,0090	0,0125	0,0210	0,0325	0,0520	0,0865	0,1335	0,1820	0,2375	0,3090	0,3730	0,4275	0,4760	0,5140	0,5400	0,5605	0,5750	0,5880	0,6005	0,6105	0,6160	0,6195	0,6225	0,6230	0,6225
MRSA	A01	4	1	irradiated group	0,0115	0,0145	0,0180	0,0280	0,0420	0,0645	0,0945	0,1385	0,1840	0,2435	0,3220	0,3835	0,4365	0,4830	0,5195	0,5460	0,5620	0,5765	0,5865	0,5960	0,6035	0,6080	0,6130	0,6160	0,6155
MRSA	A02	4	1	control group	0,0055	0,0085	0,0120	0,0220	0,0370	0,0655	0,1065	0,1565	0,2080	0,2745	0,3520	0,4075	0,4575	0,5000	0,5325	0,5550	0,5690	0,5835	0,5955	0,6050	0,6105	0,6140	0,6180	0,6200	0,6185
MRSA	A03	4	2	irradiated group	0,0075	0,0125	0,0170	0,0270	0,0400	0,0615	0,0915	0,1375	0,1840	0,2405	0,3150	0,3795	0,4395	0,4890	0,5255	0,5520	0,5670	0,5815	0,5915	0,6010	0,6075	0,6140	0,6210	0,6250	0,6265
MRSA	A04	4	2	control group	0,0075	0,0115	0,0170	0,0270	0,0430	0,0725	0,1155	0,1675	0,2190	0,2845	0,3590	0,4145	0,4625	0,5040	0,5335	0,5550	0,5690	0,5855	0,5965	0,6070	0,6125	0,6160	0,6200	0,6230	0,6225
MRSA	C01	4	3	irradiated group	0,0135	0,0175	0,0210	0,0300	0,0450	0,0655	0,0945	0,1385	0,1860	0,2435	0,3190	0,3795	0,4355	0,4850	0,5225	0,5500	0,5670	0,5815	0,5925	0,6020	0,6095	0,6160	0,6220	0,6280	0,6305
MRSA	C02	4	3	control group	0,0075	0,0125	0,0160	0,0290	0,0450	0,0775	0,1215	0,1725	0,2280	0,2985	0,3710	0,4245	0,4725	0,5100	0,5385	0,5590	0,5730	0,5885	0,5985	0,6080	0,6135	0,6170	0,6210	0,6230	0,6225
MRSA	C03	4	4	irradiated group	0,0075	0,0125	0,0180	0,0270	0,0390	0,0615	0,0935	0,1395	0,1870	0,2445	0,3180	0,3815	0,4375	0,4870	0,5235	0,5510	0,5670	0,5825	0,5925	0,6030	0,6105	0,6160	0,6230	0,6270	0,6285
MRSA	C04	4	4	control group	0,0105	0,0165	0,0210	0,0330	0,0510	0,0835	0,1295	0,1815	0,2360	0,3045	0,3750	0,4295	0,4765	0,5150	0,5425	0,5620	0,5760	0,5925	0,6025	0,6110	0,6165	0,6200	0,6240	0,6260	0,6255
MRSA	A01	5	1	irradiated group	0,0100	0,0130	0,0195	0,0260	0,0400	0,0600	0,0885	0,1320	0,1770	0,2350	0,3040	0,3710	0,4285	0,4795	0,5195	0,5465	0,5665	0,5795	0,5915	0,6010	0,6095	0,6135	0,6155	0,6160	0,6175
MRSA	A02	5	1	control group	0,0090	0,0110	0,0165	0,0250	0,0420	0,0720	0,1165	0,1680	0,2200	0,2880	0,3590	0,4160	0,4695	0,5125	0,5425	0,5635	0,5825	0,5945	0,6075	0,6170	0,6235	0,6245	0,6235	0,6230	0,6225
MRSA	A03	5	2	irradiated group	0,0070	0,0140	0,0205	0,0260	0,0400	0,0580	0,0885	0,1330	0,1780	0,2330	0,3000	0,3710	0,4375	0,4925	0,5325	0,5615	0,5825	0,5955	0,6075	0,6170	0,6255	0,6285	0,6315	0,6330	0,6345
MRSA	A04	5	2	control group	0,0070	0,0120	0,0175	0,0260	0,0440	0,0740	0,1195	0,1710	0,2220	0,2890	0,3580	0,4160	0,4645	0,5085	0,5395	0,5605	0,5795	0,5935	0,6065	0,6160	0,6245	0,6255	0,6245	0,6240	0,6235
MRSA	C01	5	3	irradiated group	0,0100	0,0180	0,0225	0,0280	0,0420	0,0600	0,0905	0,1360	0,1830	0,2390	0,3080	0,3740	0,4345	0,4885	0,5295	0,5575	0,5785	0,5915	0,6025	0,6130	0,6205	0,6255	0,6275	0,6300	0,6315
MRSA	C02	5	3	control group	0,0080	0,0110	0,0155	0,0240	0,0420	0,0720	0,1165	0,1670	0,2190	0,2890	0,3610	0,4170	0,4705	0,5095	0,5405	0,5625	0,5815	0,5945	0,6065	0,6170	0,6235	0,6245	0,6245	0,6240	0,6235
MRSA	C03	5	4	irradiated group	0,0060	0,0140	0,0195	0,0290	0,0450	0,0700	0,1095	0,1570	0,2040	0,2680	0,3400	0,4020	0,4595	0,5055	0,5405	0,5635	0,5805	0,5925	0,6035	0,6130	0,6205	0,6235	0,6255	0,6260	0,6275
MRSA	C04	5	4	control group	0,0080	0,0130	0,0185	0,0270	0,0450	0,0800	0,1245	0,1750	0,2260	0,2950	0,3660	0,4220	0,4725	0,5115	0,5415	0,5625	0,5805	0,5945	0,6065	0,6160	0,6225	0,6245	0,6225	0,6220	0,6215
MRSA	A01	6	1	irradiated group	0,0110	0,0155	0,0210	0,0305	0,0465	0,0720	0,1055	0,1505	0,1985	0,2605	0,3320	0,3935	0,4510	0,4960	0,5325	0,5560	0,5700	0,5825	0,5925	0,6005	0,6075	0,6105	0,6150	0,6180	0,6175
MRSA	A02	6	1	control group	0,0070	0,0105	0,0160	0,0255	0,0435	0,0750	0,1195	0,1685	0,2225	0,2905	0,3620	0,4155	0,4660	0,5070	0,5375	0,5570	0,5740	0,5885	0,5995	0,6075	0,6125	0,6135	0,6160	0,6170	0,6155
MRSA	A03	6	2	irradiated group	0,0050	0,0135	0,0200	0,0285	0,0425	0,0680	0,1005	0,1475	0,1935	0,2525	0,3230	0,3895	0,4540	0,5020	0,5405	0,5650	0,5810	0,5945	0,6035	0,6125	0,6195	0,6225	0,6280	0,6310	0,6315
MRSA	A04	6	2	control group	0,0050	0,0105	0,0170	0,0265	0,0435	0,0730	0,1175	0,1675	0,2185	0,2815	0,3550	0,4095	0,4590	0,5010	0,5325	0,5550	0,5710	0,5875	0,5995	0,6075	0,6135	0,6155	0,6180	0,6210	0,6185
MRSA	C01	6	3	irradiated group	0,0090	0,0185	0,0220	0,0315	0,0475	0,0750	0,1095	0,1555	0,2045	0,2675	0,3380	0,4005	0,4580	0,5060	0,5415	0,5640	0,5780	0,5915	0,6015	0,6095	0,6165	0,6205	0,6260	0,6290	0,6275
MRSA	C02	6	3	control group	0,0060	0,0095	0,0150	0,0255	0,0425	0,0760	0,1195	0,1675	0,2225	0,2915	0,3620	0,4185	0,4680	0,5060	0,5365	0,5580	0,5740	0,5895	0,5995	0,6075	0,6135	0,6145	0,6180	0,6200	0,6175
MRSA	C03	6	4	irradiated group	0,0040	0,0105	0,0180	0,0295	0,0475	0,0760	0,1135	0,1605	0,2085	0,2715	0,3460	0,4065	0,4620	0,5070	0,5405	0,5630	0,5780	0,5905	0,5995	0,6075	0,6145	0,6175	0,6220	0,6260	0,6255
MRSA	C04	6	4	control group	0,0030	0,0095	0,0180	0,0265	0,0465	0,0760	0,1235	0,1725	0,2275	0,2915	0,3630	0,4175	0,4650	0,5040	0,5335	0,5550	0,5720	0,5865	0,5975	0,6065	0,6115	0,6135	0,6160	0,6180	0,6155
MRSA	A01	0	5	irradiated group	0,0090	0,0125	0,0195	0,0310	0,0560	0,0900	0,1330	0,1805	0,2345	0,3030	0,3645	0,4155	0,4590	0,4945	0,5230	0,5405	0,5565	0,5730	0,5835	0,5930	0,5995	0,6055	0,6095	0,6080	0,6075
MRSA	A02	0	5	control group	0,0060	0,0125	0,0195	0,0320	0,0580	0,0950	0,1430	0,1925	0,2465	0,3140	0,3765	0,4255	0,4740	0,5095	0,5360	0,5545	0,5695	0,5850	0,5975	0,6050	0,6115	0,6165	0,6215	0,6200	0,6185
MRSA	A03	0	6	irradiated group	0,0020	0,0085	0,0175	0,0310	0,0560	0,0950	0,1400	0,1875	0,2415	0,3090	0,3715	0,4215	0,4680	0,5035	0,5300	0,5475	0,5635	0,5780	0,5915	0,6000	0,6075	0,6125	0,6155	0,6140	0,6135
MRSA	A04	0	6	control group	0,0020	0,0095	0,0185	0,0330	0,0600	0,1000	0,1450	0,1935	0,2455	0,3140	0,3745	0,4235	0,4690	0,5025	0,5300	0,5465	0,5615	0,5770	0,5895	0,5990	0,6055	0,6115	0,6145	0,6140	0,6125
MRSA	C01	0	7	irradiated group	0,0070	0,0125	0,0185	0,0320	0,0570	0,0970	0,1410	0,1895	0,2425	0,3110	0,3715	0,4225	0,4670	0,5015	0,5300	0,5485	0,5635	0,5780	0,5905	0,6010	0,6075	0,6135	0,6175	0,6180	0,6175
MRSA	C02	0	7	control group	0,0040	0,0095	0,0175	0,0310	0,0570	0,0970	0,1430	0,1915	0,2475	0,3150	0,3765	0,4255	0,4710	0,5055	0,5330	0,5515	0,5665	0,5820	0,5955	0,6040	0,6115	0,6165	0,6195	0,6200	0,6185
MRSA	C03	0	8	irradiated group	-0,0010	0,0085	0,0175	0,0310	0,0580	0,0990	0,1450	0,1925	0,2475	0,3180	0,3765	0,4265	0,4720	0,5055	0,5340	0,5515	0,5665	0,5820	0,5935	0,6030	0,6085	0,6135	0,6175	0,6160	0,6145
MRSA	C04	0	8	control group	0,0020	0,0115	0,0195	0,0370	0,0640	0,1040	0,1500	0,1975	0,2555	0,3220	0,3815	0,4295	0,4740	0,5055	0,5320	0,5495	0,5645	0,5800	0,5915	0,6010	0,6075	0,6125	0,6155	0,6150	0,6135
MRSA	A01	1	5	irradiated group	0,0085	0,0130	0,0155	0,0235	0,0375	0,0555	0,0855	0,1290	0,1725	0,2270	0,2950	0,3615	0,4200	0,4720	0,5130	0,5445	0,5640	0,5795	0,5915	0,6010	0,6100	0,6160	0,6230	0,6265	0,6305
MRSA	A02	1	5	control group	0,0075	0,0100	0,0125	0,0215	0,0395	0,0665	0,1095	0,1610	0,2105	0,2740	0,3460	0,4105	0,4710	0,5150	0,5490	0,5705	0,5860	0,6005	0,6115	0,6190	0,6280	0,6340	0,6390	0,6415	0,6425
MRSA	A03	1	6	irradiated group	0,0025	0,0100	0,0135	0,0215	0,0325	0,0505	0,0775	0,1190	0,1665	0,2170	0,2810	0,3505	0,4130	0,4700	0,5130	0,5445	0,5670	0,5825	0,5955	0,6050	0,6140	0,6220	0,6280	0,6325	0,6365
MRSA	A04	1	6	control group	0,0045	0,0090	0,0135	0,0245	0,0415	0,0695	0,1145	0,1650	0,2155	0,2780	0,3480	0,4125	0,4720	0,5130	0,5460	0,5655	0,5820	0,5955	0,6075	0,6160	0,6240	0,6310	0,6360	0,6385	0,6405
MRSA	C01	1	7	irradiated group	0,0085	0,0160	0,0185	0,0255	0,0405	0,0585	0,0865	0,1310	0,1775	0,2320	0,2980	0,3625	0,4240	0,4750	0,5180	0,5485	0,5690	0,5855	0,5985	0,6070	0,6170	0,6240	0,6300	0,6345	0,6395
MRSA	C02	1	7	control group	0,0085	0,0150	0,0185	0,0285	0,0475	0,0775	0,1255	0,1720	0,2275	0,2950	0,3650	0,4265	0,4820	0,5220	0,5550	0,5745	0,5890	0,6035	0,6135	0,6220	0,6300	0,6360	0,6410	0,6435	0,6455
MRSA	C03	1	8	irradiated group	0,0025	0,0120	0,0155	0,0255	0,0425	0,0655	0,1025	0,1530	0,2005	0,2610	0,3310	0,3935	0,4510	0,4970	0,5350	0,5595	0,5770	0,5915	0,6035	0,6120	0,6200	0,6260	0,6330	0,6355	0,6395
MRSA	C04	1	8	control group	0,0075	0,0120	0,0195	0,0295	0,0475	0,0755	0,1235	0,1730	0,2235	0,2890	0,3600	0,4215	0,4780	0,5190	0,5510	0,5705	0,5870	0,6005	0,6115	0,6190	0,6280	0,6340	0,6390	0,6405	0,6425
MRSA	A01	2	5	irradiated group	0,0110	0,0155	0,0195	0,0280	0,0410	0,0640	0,0920	0,1345	0,1830	0,2390	0,3060	0,3720	0,4320	0,4830	0,5220	0,5515	0,5700	0,5825	0,5940	0,6030	0,6095	0,6160	0,6205	0,6250	0,6255
MRSA	A02	2	5	control group	0,0110	0,0135	0,0175	0,0260	0,0420	0,0700	0,1110	0,1595	0,2110	0,2760	0,3450	0,4140	0,4720	0,5160	0,5520	0,5745	0,5890	0,6015	0,6140	0,6210	0,6275	0,6330	0,6385	0,6410	0,6405
MRSA	A03	2	6	irradiated group	0,0040	0,0075	0,0135	0,0200	0,0320	0,0480	0,0750	0,1145	0,1610	0,2120	0,2730	0,3440	0,4100	0,4660	0,5120	0,5445	0,5670	0,5815	0,5930	0,6020	0,6105	0,6170	0,6225	0,6270	0,6285
MRSA	A04	2	6	control group	0,0060	0,0095	0,0165	0,0260	0,0420	0,0710	0,1150	0,1645	0,2170	0,2800	0,3490	0,4140	0,4740	0,5160	0,5490	0,5715	0,5870	0,5985	0,6090	0,6170	0,6245	0,6310	0,6345	0,6380	0,6375
MRSA	C01	2	7	irradiated group	0,0060	0,0125	0,0165	0,0240	0,0370	0,0610	0,0890	0,1335	0,1840	0,2380	0,3050	0,3700	0,4300	0,4830	0,5250	0,5545	0,5740	0,5865	0,5990	0,6060	0,6145	0,6220	0,6275	0,6320	0,6335
MRSA	C02	2	7	control group	0,0070	0,0115	0,0175	0,0290	0,0470	0,0770	0,1250	0,1745	0,2300	0,2990	0,3680	0,4290	0,4840	0,5250	0,5570	0,5775	0,5910	0,6035	0,6150	0,6220	0,6275	0,6340	0,6385	0,6410	0,6395
MRSA	C03	2	8	irradiated group	0,0010	0,0075	0,0135	0,0230	0,0380	0,0640	0,1020	0,1515	0,2030	0,2650	0,3330	0,3960	0,4560	0,5030	0,5400	0,5635	0,5800	0,5925	0,6030	0,6110	0,6175	0,6230	0,6285	0,6320	0,6315
MRSA	C04	2	8	control group	0,0070	0,0115	0,0175	0,0280	0,0450	0,0760	0,1220	0,1715	0,2260	0,2900	0,3590	0,4230	0,4800	0,5210	0,5540	0,5755	0,5900	0,6025	0,6130	0,6210	0,6275	0,6330	0,6375	0,6400	0,6395
MRSA	A01	3	5	irradiated group	0,0115	0,0155	0,0200	0,0295	0,0460	0,0695	0,1040	0,1500	0,1995	0,2615	0,3285	0,3920	0,4505	0,5005	0,5375	0,5615	0,5765	0,5895	0,5980	0,6075	0,6175	0,6215	0,6260	0,6305	0,6305
MRSA	A02	3	5	control group	0,0095	0,0125	0,0170	0,0265	0,0430	0,0715	0,1140	0,1630	0,2135	0,2825	0,3525	0,4140	0,4705	0,5145	0,5465	0,5675	0,5825	0,5965	0,6070	0,6165	0,6245	0,6305	0,6350	0,6385	0,6365
MRSA	A03	3	6	irradiated group	0,0075	0,0125	0,0170	0,0255	0,0400	0,0635	0,0980	0,1460	0,1935	0,2565	0,3225	0,3880	0,4495	0,5015	0,5395	0,5645	0,5805	0,5935	0,6040	0,6145	0,6225	0,6275	0,6330	0,6375	0,6375
MRSA	A04	3	6	control group	0,0085	0,0125	0,0180	0,0275	0,0450	0,0735	0,1190	0,1700	0,2205	0,2845	0,3535	0,4160	0,4705	0,5115	0,5445	0,5655	0,5795	0,5925	0,6030	0,6135	0,6225	0,6275	0,6330	0,6365	0,6345
MRSA	C01	3	7	irradiated group	0,0105	0,0155	0,0200	0,0285	0,0450	0,0685	0,1040	0,1530	0,2015	0,2645	0,3325	0,3960	0,4525	0,5045	0,5435	0,5675	0,5825	0,5955	0,6050	0,6145	0,6235	0,6285	0,6350	0,6395	0,6385
MRSA	C02	3	7	control group	0,0085	0,0135	0,0170	0,0245	0,0430	0,0735	0,1180	0,1660	0,2175	0,2835	0,3535	0,4130	0,4685	0,5115	0,5445	0,5665	0,5805	0,5945	0,6050	0,6165	0,6245	0,6295	0,6340	0,6375	0,6355
MRSA	C03	3	8	irradiated group	0,0065	0,0135	0,0180	0,0255	0,0420	0,0705	0,1120	0,1610	0,2095	0,2775	0,3445	0,4040	0,4615	0,5065	0,5425	0,5645	0,5785	0,5915	0,6010	0,6115	0,6195	0,6245	0,6300	0,6345	0,6335
MRSA	C04	3	8	control group	0,0065	0,0135	0,0190	0,0285	0,0460	0,0785	0,1250	0,1740	0,2245	0,2895	0,3595	0,4190	0,4705	0,5115	0,5445	0,5655	0,5785	0,5925	0,6030	0,6135	0,6225	0,6275	0,6320	0,6355	0,6325
MRSA	A01	4	5	irradiated group	0,0140	0,0170	0,0165	0,0285	0,0470	0,0685	0,1005	0,1440	0,1935	0,2525	0,3205	0,3850	0,4445	0,4950	0,5310	0,5555	0,5705	0,5830	0,5920	0,6005	0,6070	0,6125	0,6170	0,6210	0,6225
MRSA	A02	4	5	control group	0,0090	0,0150	0,0135	0,0265	0,0430	0,0705	0,1125	0,1620	0,2135	0,2745	0,3455	0,4080	0,4645	0,5100	0,5410	0,5625	0,5775	0,5910	0,6020	0,6095	0,6160	0,6225	0,6250	0,6280	0,6275
MRSA	A03	4	6	irradiated group	0,0070	0,0130	0,0145	0,0265	0,0430	0,0655	0,0975	0,1430	0,1905	0,2465	0,3135	0,3810	0,4455	0,4970	0,5350	0,5595	0,5765	0,5890	0,6000	0,6075	0,6140	0,6195	0,6240	0,6280	0,6295
MRSA	A04	4	6	control group	0,0070	0,0110	0,0145	0,0285	0,0460	0,0755	0,1195	0,1690	0,2205	0,2815	0,3505	0,4120	0,4685	0,5100	0,5400	0,5615	0,5755	0,5880	0,5990	0,6075	0,6140	0,6205	0,6240	0,6270	0,6265
MRSA	C01	4	7	irradiated group	0,0100	0,0130	0,0155	0,0305	0,0440	0,0675	0,0995	0,1460	0,1945	0,2525	0,3195	0,3830	0,4415	0,4970	0,5350	0,5615	0,5765	0,5890	0,5990	0,6075	0,6140	0,6205	0,6240	0,6290	0,6305
MRSA	C02	4	7	control group	0,0080	0,0120	0,0135	0,0265	0,0440	0,0755	0,1175	0,1650	0,2195	0,2845	0,3535	0,4160	0,4685	0,5110	0,5430	0,5655	0,5795	0,5930	0,6030	0,6125	0,6190	0,6235	0,6260	0,6290	0,6285
MRSA	C03	4	8	irradiated group	0,0080	0,0090	0,0145	0,0285	0,0430	0,0695	0,1075	0,1550	0,2055	0,2685	0,3365	0,3990	0,4575	0,5040	0,5390	0,5615	0,5765	0,5880	0,5990	0,6075	0,6140	0,6185	0,6220	0,6260	0,6275
MRSA	C04	4	8	control group	0,0070	0,0110	0,0145	0,0305	0,0480	0,0795	0,1275	0,1760	0,2305	0,2935	0,3615	0,4230	0,4755	0,5160	0,5460	0,5655	0,5785	0,5910	0,6030	0,6115	0,6180	0,6225	0,6250	0,6280	0,6265
MRSA	A01	5	5	irradiated group	0,0180	0,0235	0,0265	0,0350	0,0515	0,0775	0,1120	0,1580	0,2080	0,2705	0,3405	0,4020	0,4635	0,5105	0,5445	0,5670	0,5815	0,5935	0,6015	0,6105	0,6180	0,6235	0,6275	0,6290	0,6310
MRSA	A02	5	5	control group	0,0080	0,0145	0,0175	0,0270	0,0495	0,0845	0,1340	0,1870	0,2420	0,3085	0,3825	0,4420	0,4905	0,5305	0,5575	0,5740	0,5905	0,6025	0,6135	0,6235	0,6300	0,6355	0,6375	0,6360	0,6350
MRSA	A03	5	6	irradiated group	0,0110	0,0195	0,0235	0,0320	0,0465	0,0695	0,1050	0,1510	0,1990	0,2575	0,3285	0,3950	0,4605	0,5095	0,5465	0,5700	0,5865	0,5995	0,6085	0,6175	0,6240	0,6305	0,6345	0,6360	0,6380
MRSA	A04	5	6	control group	0,0080	0,0155	0,0215	0,0320	0,0545	0,0925	0,1430	0,1950	0,2510	0,3195	0,3885	0,4440	0,4925	0,5295	0,5555	0,5710	0,5855	0,5995	0,6105	0,6205	0,6270	0,6325	0,6335	0,6340	0,6330
MRSA	C01	5	7	irradiated group	0,0180	0,0215	0,0265	0,0360	0,0515	0,0765	0,1120	0,1610	0,2100	0,2695	0,3435	0,4050	0,4655	0,5135	0,5505	0,5720	0,5885	0,5995	0,6095	0,6185	0,6270	0,6325	0,6365	0,6380	0,6410
MRSA	C02	5	7	control group	0,0090	0,0145	0,0185	0,0300	0,0525	0,0895	0,1410	0,1920	0,2470	0,3175	0,3875	0,4470	0,4935	0,5305	0,5585	0,5750	0,5895	0,6035	0,6145	0,6235	0,6300	0,6345	0,6365	0,6360	0,6360
MRSA	C03	5	8	irradiated group	0,0110	0,0215	0,0255	0,0340	0,0515	0,0795	0,1220	0,1710	0,2240	0,2875	0,3615	0,4220	0,4795	0,5215	0,5535	0,5740	0,5885	0,6015	0,6115	0,6195	0,6270	0,6325	0,6365	0,6380	0,6390
MRSA	C04	5	8	control group	0,0100	0,0175	0,0235	0,0360	0,0595	0,1005	0,1500	0,2000	0,2560	0,3265	0,3945	0,4510	0,4985	0,5325	0,5585	0,5750	0,5895	0,6035	0,6135	0,6225	0,6300	0,6335	0,6355	0,6340	0,6340
MRSA	A01	6	5	irradiated group	0,0120	0,0165	0,0215	0,0320	0,0485	0,0755	0,1125	0,1610	0,2125	0,2765	0,3525	0,4265	0,4935	0,5485	0,5905	0,6170	0,6365	0,6515	0,6590	0,6625	0,6675	0,6700	0,6740	0,6725	0,6720
MRSA	A02	6	5	control group	0,0080	0,0115	0,0175	0,0270	0,0455	0,0795	0,1295	0,1810	0,2355	0,3055	0,3795	0,4465	0,5075	0,5555	0,5915	0,6180	0,6385	0,6565	0,6660	0,6715	0,6755	0,6790	0,6780	0,6765	0,6740
MRSA	A03	6	6	irradiated group	0,0060	0,0145	0,0195	0,0290	0,0445	0,0685	0,1065	0,1550	0,2035	0,2665	0,3415	0,4175	0,4905	0,5455	0,5905	0,6180	0,6395	0,6555	0,6630	0,6675	0,6715	0,6740	0,6770	0,6785	0,6790
MRSA	A04	6	6	control group	0,0060	0,0145	0,0205	0,0320	0,0525	0,0865	0,1375	0,1890	0,2445	0,3125	0,3845	0,4505	0,5085	0,5535	0,5905	0,6150	0,6345	0,6505	0,6610	0,6665	0,6695	0,6730	0,6750	0,6735	0,6700
MRSA	C01	6	7	irradiated group	0,0110	0,0185	0,0225	0,0320	0,0495	0,0755	0,1145	0,1630	0,2145	0,2805	0,3555	0,4265	0,4945	0,5515	0,5945	0,6210	0,6415	0,6565	0,6650	0,6695	0,6735	0,6780	0,6800	0,6805	0,6820
MRSA	C02	6	7	control group	0,0070	0,0135	0,0185	0,0290	0,0485	0,0845	0,1345	0,1860	0,2415	0,3145	0,3885	0,4535	0,5095	0,5565	0,5925	0,6170	0,6385	0,6555	0,6650	0,6705	0,6735	0,6760	0,6770	0,6735	0,6710
MRSA	C03	6	8	irradiated group	0,0050	0,0155	0,0195	0,0320	0,0475	0,0765	0,1185	0,1690	0,2215	0,2885	0,3645	0,4345	0,5005	0,5535	0,5945	0,6210	0,6415	0,6565	0,6640	0,6685	0,6725	0,6750	0,6780	0,6775	0,6790
MRSA	C04	6	8	control group	0,0100	0,0185	0,0245	0,0380	0,0625	0,1025	0,1535	0,2050	0,2635	0,3355	0,4055	0,4695	0,5235	0,5665	0,6005	0,6230	0,6435	0,6595	0,6680	0,6725	0,6745	0,6760	0,6760	0,6735	0,6710
				**Messzeitpunkt**	**0 h**	**0 h 30 min**	**1 h**	**1 h 30 min**	**2 h**	**2 h 30 min**	**3 h**	**3 h 30 min**	**4 h**	**4 h 30 min**	**5 h**	**5 h 30 min**	**6 h**	**6 h 30 min**	**7 h**	**7 h 30 min**	**8 h**	**8 h 30 min**	**9 h**	**9 h 30 min**	**10 h**	**10 h 30 min**	**11 h**	**11 h 30 min**	**12 h**
Erreger	Position - 12-Well-Mikro-titerplatte	Stage	Stichproben-anzahl	Versuchsgruppe	Blank corrected based on Raw Data (600)	Blank corrected based on Raw Data (600)	Blank corrected based on Raw Data (600)	Blank corrected based on Raw Data (600)	Blank corrected based on Raw Data (600)	Blank corrected based on Raw Data (600)	Blank corrected based on Raw Data (600)	Blank corrected based on Raw Data (600)	Blank corrected based on Raw Data (600)	Blank corrected based on Raw Data (600)	Blank corrected based on Raw Data (600)	Blank corrected based on Raw Data (600)	Blank corrected based on Raw Data (600)	Blank corrected based on Raw Data (600)	Blank corrected based on Raw Data (600)	Blank corrected based on Raw Data (600)	Blank corrected based on Raw Data (600)	Blank corrected based on Raw Data (600)	Blank corrected based on Raw Data (600)	Blank corrected based on Raw Data (600)	Blank corrected based on Raw Data (600)	Blank corrected based on Raw Data (600)	Blank corrected based on Raw Data (600)	Blank corrected based on Raw Data (600)	Blank corrected based on Raw Data (600)
Pseudomonas aeruginosa	A01	0	1	irradiated group	0,0095	0,0120	0,0205	0,0320	0,0515	0,0695	0,0900	0,1155	0,1460	0,1880	0,2360	0,2840	0,3275	0,3665	0,4120	0,4830	0,5530	0,6000	0,6300	0,6525	0,6680	0,6770	0,6825	0,6850	0,6860
Pseudomonas aeruginosa	A02	0	1	control group	0,0065	0,0100	0,0185	0,0290	0,0495	0,0675	0,0910	0,1165	0,1500	0,1950	0,2470	0,2980	0,3445	0,3845	0,4460	0,5210	0,5830	0,6260	0,6500	0,6655	0,6760	0,6810	0,6865	0,6880	0,6890
Pseudomonas aeruginosa	A03	0	2	irradiated group	0,0045	0,0100	0,0185	0,0300	0,0515	0,0705	0,0920	0,1195	0,1530	0,1980	0,2510	0,3010	0,3495	0,3925	0,4570	0,5280	0,5880	0,6330	0,6520	0,6655	0,6760	0,6820	0,6875	0,6880	0,6890
Pseudomonas aeruginosa	A04	0	2	control group	0,0065	0,0130	0,0235	0,0360	0,0575	0,0765	0,1000	0,1255	0,1600	0,2040	0,2550	0,3060	0,3515	0,3915	0,4480	0,5210	0,5860	0,6250	0,6460	0,6645	0,6750	0,6810	0,6865	0,6870	0,6880
Pseudomonas aeruginosa	C01	0	3	irradiated group	0,0145	0,0200	0,0305	0,0430	0,0625	0,0825	0,1030	0,1295	0,1590	0,2030	0,2530	0,3050	0,3505	0,3895	0,4330	0,5060	0,5690	0,6150	0,6420	0,6615	0,6740	0,6810	0,6875	0,6890	0,6890
Pseudomonas aeruginosa	C02	0	3	control group	0,0075	0,0140	0,0235	0,0350	0,0555	0,0745	0,0970	0,1245	0,1560	0,2030	0,2550	0,3060	0,3515	0,3945	0,4530	0,5260	0,5870	0,6300	0,6510	0,6675	0,6770	0,6840	0,6885	0,6890	0,6890
Pseudomonas aeruginosa	C03	0	4	irradiated group	0,0055	0,0120	0,0235	0,0370	0,0575	0,0765	0,0970	0,1255	0,1610	0,2070	0,2590	0,3090	0,3545	0,3995	0,4610	0,5310	0,5900	0,6340	0,6540	0,6695	0,6780	0,6830	0,6885	0,6890	0,6890
Pseudomonas aeruginosa	C04	0	4	control group	0,0085	0,0170	0,0265	0,0410	0,0645	0,0865	0,1080	0,1355	0,1680	0,2110	0,2610	0,3100	0,3555	0,3975	0,4520	0,5210	0,5840	0,6260	0,6480	0,6655	0,6770	0,6820	0,6875	0,6880	0,6880
Pseudomonas aeruginosa	A01	1	1	irradiated group	0,0305	0,0380	0,0500	0,0680	0,0965	0,1150	0,1445	0,1840	0,2305	0,2870	0,3485	0,4000	0,4435	0,5065	0,5660	0,6065	0,6360	0,6575	0,6695	0,6775	0,6820	0,6870	0,6885	0,6930	0,6950
Pseudomonas aeruginosa	A02	1	1	control group	0,0265	0,0350	0,0550	0,0760	0,1015	0,1300	0,1725	0,2320	0,2985	0,3590	0,4125	0,4690	0,5345	0,5895	0,6280	0,6495	0,6640	0,6735	0,6795	0,6835	0,6870	0,6910	0,6925	0,6960	0,6970
Pseudomonas aeruginosa	A03	1	2	irradiated group	0,0215	0,0260	0,0320	0,0380	0,0525	0,0670	0,0845	0,1040	0,1285	0,1630	0,2175	0,2720	0,3245	0,3705	0,4290	0,5045	0,5710	0,6125	0,6425	0,6615	0,6730	0,6820	0,6855	0,6910	0,6930
Pseudomonas aeruginosa	A04	1	2	control group	0,0195	0,0300	0,0520	0,0740	0,0995	0,1280	0,1685	0,2170	0,2785	0,3380	0,3885	0,4430	0,5105	0,5715	0,6160	0,6435	0,6600	0,6715	0,6785	0,6825	0,6860	0,6900	0,6905	0,6950	0,6960
Pseudomonas aeruginosa	C01	1	3	irradiated group	0,0325	0,0400	0,0510	0,0660	0,0915	0,1140	0,1415	0,1790	0,2255	0,2800	0,3435	0,3960	0,4375	0,4935	0,5560	0,6045	0,6360	0,6565	0,6695	0,6785	0,6830	0,6890	0,6905	0,6940	0,6960
Pseudomonas aeruginosa	C02	1	3	control group	0,0205	0,0320	0,0510	0,0690	0,0945	0,1230	0,1595	0,2160	0,2755	0,3390	0,3925	0,4460	0,5145	0,5745	0,6170	0,6455	0,6610	0,6725	0,6795	0,6835	0,6870	0,6910	0,6915	0,6960	0,6960
Pseudomonas aeruginosa	C03	1	4	irradiated group	0,0175	0,0250	0,0300	0,0360	0,0535	0,0720	0,0895	0,1100	0,1375	0,1800	0,2345	0,2870	0,3415	0,3885	0,4540	0,5285	0,5860	0,6235	0,6495	0,6665	0,6760	0,6840	0,6875	0,6920	0,6940
Pseudomonas aeruginosa	C04	1	4	control group	0,0255	0,0380	0,0660	0,0870	0,1165	0,1510	0,1965	0,2580	0,3205	0,3850	0,4385	0,4880	0,5485	0,5985	0,6320	0,6505	0,6650	0,6735	0,6795	0,6835	0,6860	0,6910	0,6905	0,6940	0,6960
Pseudomonas aeruginosa	A01	2	1	irradiated group	0,0320	0,0345	0,0440	0,0610	0,0780	0,1015	0,1280	0,1645	0,2130	0,2755	0,3295	0,3835	0,4285	0,4750	0,5335	0,5780	0,6065	0,6285	0,6585	0,6730	0,6775	0,6815	0,6835	0,6840	0,6835
Pseudomonas aeruginosa	A02	2	1	control group	0,0270	0,0315	0,0500	0,0690	0,0880	0,1135	0,1520	0,2065	0,2740	0,3355	0,3885	0,4395	0,5025	0,5590	0,5985	0,6240	0,6375	0,6495	0,6735	0,6830	0,6835	0,6855	0,6865	0,6870	0,6855
Pseudomonas aeruginosa	A03	2	2	irradiated group	0,0290	0,0345	0,0440	0,0590	0,0750	0,0965	0,1200	0,1495	0,1940	0,2465	0,3035	0,3555	0,4005	0,4590	0,5265	0,5760	0,6095	0,6325	0,6635	0,6760	0,6785	0,6835	0,6845	0,6850	0,6845
Pseudomonas aeruginosa	A04	2	2	control group	0,0250	0,0335	0,0560	0,0750	0,0960	0,1265	0,1690	0,2255	0,2920	0,3585	0,4065	0,4615	0,5255	0,5760	0,6105	0,6300	0,6395	0,6525	0,6755	0,6830	0,6835	0,6855	0,6865	0,6860	0,6845
Pseudomonas aeruginosa	C01	2	3	irradiated group	0,0350	0,0395	0,0520	0,0710	0,0930	0,1225	0,1560	0,1975	0,2540	0,3175	0,3765	0,4315	0,4725	0,5220	0,5715	0,6030	0,6245	0,6405	0,6685	0,6800	0,6815	0,6845	0,6855	0,6860	0,6855
Pseudomonas aeruginosa	C02	2	3	control group	0,0290	0,0365	0,0560	0,0780	0,1030	0,1375	0,1860	0,2475	0,3170	0,3795	0,4235	0,4825	0,5415	0,5860	0,6175	0,6350	0,6435	0,6535	0,6765	0,6840	0,6845	0,6865	0,6875	0,6870	0,6865
Pseudomonas aeruginosa	C03	2	4	irradiated group	0,0260	0,0345	0,0460	0,0630	0,0830	0,1075	0,1370	0,1755	0,2280	0,2865	0,3505	0,4035	0,4535	0,5140	0,5655	0,6020	0,6255	0,6435	0,6705	0,6800	0,6815	0,6845	0,6855	0,6860	0,6845
Pseudomonas aeruginosa	C04	2	4	control group	0,0370	0,0515	0,0770	0,1070	0,1380	0,1815	0,2420	0,3105	0,3800	0,4365	0,4825	0,5355	0,5815	0,6150	0,6335	0,6430	0,6485	0,6575	0,6795	0,6850	0,6845	0,6865	0,6865	0,6860	0,6855
Pseudomonas aeruginosa	A01	3	1	irradiated group	0,0255	0,0300	0,0350	0,0495	0,0625	0,0795	0,1005	0,1275	0,1650	0,2160	0,2670	0,3170	0,3640	0,4105	0,4820	0,5460	0,5930	0,6260	0,6490	0,6625	0,6700	0,6760	0,6815	0,6835	0,6840
Pseudomonas aeruginosa	A02	3	1	control group	0,0315	0,0410	0,0560	0,0785	0,1015	0,1345	0,1785	0,2405	0,3080	0,3670	0,4140	0,4760	0,5410	0,5925	0,6280	0,6490	0,6620	0,6690	0,6760	0,6805	0,6820	0,6850	0,6875	0,6895	0,6880
Pseudomonas aeruginosa	A03	3	2	irradiated group	0,0275	0,0350	0,0460	0,0615	0,0755	0,0945	0,1175	0,1515	0,1960	0,2500	0,3060	0,3550	0,4020	0,4715	0,5410	0,5940	0,6270	0,6470	0,6630	0,6715	0,6760	0,6810	0,6845	0,6865	0,6860
Pseudomonas aeruginosa	A04	3	2	control group	0,0245	0,0350	0,0530	0,0745	0,0955	0,1255	0,1665	0,2205	0,2840	0,3480	0,3940	0,4500	0,5220	0,5755	0,6150	0,6390	0,6580	0,6660	0,6740	0,6785	0,6800	0,6840	0,6865	0,6875	0,6870
Pseudomonas aeruginosa	C01	3	3	irradiated group	0,0425	0,0510	0,0680	0,0885	0,1105	0,1405	0,1775	0,2255	0,2870	0,3410	0,3970	0,4450	0,5060	0,5615	0,6030	0,6310	0,6510	0,6620	0,6720	0,6775	0,6790	0,6830	0,6865	0,6875	0,6870
Pseudomonas aeruginosa	C02	3	3	control group	0,0315	0,0470	0,0660	0,0895	0,1165	0,1525	0,2095	0,2725	0,3380	0,3980	0,4480	0,5080	0,5660	0,6085	0,6370	0,6540	0,6660	0,6720	0,6780	0,6815	0,6830	0,6860	0,6885	0,6895	0,6890
Pseudomonas aeruginosa	C03	3	4	irradiated group	0,0375	0,0500	0,0680	0,0855	0,1055	0,1325	0,1715	0,2165	0,2750	0,3340	0,3920	0,4410	0,5060	0,5645	0,6100	0,6380	0,6570	0,6660	0,6750	0,6795	0,6810	0,6840	0,6875	0,6875	0,6870
Pseudomonas aeruginosa	C04	3	4	control group	0,0265	0,0400	0,0610	0,0805	0,1015	0,1335	0,1755	0,2325	0,2950	0,3550	0,4050	0,4580	0,5230	0,5775	0,6180	0,6410	0,6590	0,6680	0,6750	0,6785	0,6810	0,6840	0,6865	0,6875	0,6870
Pseudomonas aeruginosa	A01	4	1	irradiated group	0,0285	0,0280	0,0325	0,0395	0,0535	0,0710	0,0900	0,1095	0,1425	0,1830	0,2300	0,2830	0,3280	0,3635	0,4205	0,4970	0,5500	0,5945	0,6195	0,6385	0,6500	0,6595	0,6655	0,6685	0,6695
Pseudomonas aeruginosa	A02	4	1	control group	0,0325	0,0380	0,0575	0,0815	0,1045	0,1370	0,1830	0,2385	0,3005	0,3500	0,3850	0,4250	0,4770	0,5245	0,5655	0,5970	0,6200	0,6375	0,6485	0,6565	0,6630	0,6675	0,6705	0,6715	0,6715
Pseudomonas aeruginosa	A03	4	2	irradiated group	0,0335	0,0380	0,0465	0,0625	0,0795	0,1020	0,1250	0,1575	0,2035	0,2550	0,3040	0,3540	0,3950	0,4645	0,5325	0,5830	0,6130	0,6335	0,6485	0,6575	0,6650	0,6695	0,6725	0,6745	0,6745
Pseudomonas aeruginosa	A04	4	2	control group	0,0255	0,0340	0,0535	0,0735	0,0975	0,1270	0,1670	0,2255	0,2915	0,3560	0,3990	0,4580	0,5230	0,5705	0,6045	0,6270	0,6420	0,6525	0,6585	0,6635	0,6690	0,6715	0,6735	0,6735	0,6735
Pseudomonas aeruginosa	C01	4	3	irradiated group	0,0375	0,0440	0,0545	0,0705	0,0905	0,1150	0,1530	0,1885	0,2385	0,2920	0,3370	0,3680	0,4230	0,4945	0,5515	0,5910	0,6170	0,6365	0,6495	0,6585	0,6660	0,6715	0,6735	0,6755	0,6755
Pseudomonas aeruginosa	C02	4	3	control group	0,0305	0,0380	0,0565	0,0805	0,1035	0,1330	0,1820	0,2375	0,3015	0,3530	0,3840	0,4120	0,4500	0,4805	0,5105	0,5390	0,5640	0,5865	0,6045	0,6195	0,6320	0,6415	0,6505	0,6555	0,6585

Pseudomonas aeruginosa	C03	4	4	irradiated group	0,0355	0,0410	0,0595	0,0755	0,0955	0,1160	0,1450	0,1795	0,2265	0,2790	0,3300	0,3810	0,4270	0,4875	0,5425	0,5830	0,6150	0,6345	0,6485	0,6575	0,6650	0,6695	0,6725	0,6735	0,6735
Pseudomonas aeruginosa	C04	4	4	control group	0,0295	0,0390	0,0585	0,0815	0,1065	0,1400	0,1860	0,2465	0,3085	0,3680	0,4160	0,4720	0,5380	0,5885	0,6255	0,6450	0,6590	0,6675	0,6715	0,6745	0,6770	0,6785	0,6785	0,6785	0,6775
Pseudomonas aeruginosa	A01	5	1	irradiated group	0,0340	0,0405	0,0555	0,0750	0,0970	0,1270	0,1660	0,2105	0,2625	0,3070	0,3480	0,4090	0,4765	0,5330	0,5740	0,6080	0,6345	0,6535	0,6660	0,6730	0,6765	0,6770	0,6795	0,6795	0,6785
Pseudomonas aeruginosa	A02	5	1	control group	0,0280	0,0295	0,0475	0,0620	0,0830	0,1140	0,1520	0,2045	0,2525	0,2950	0,3540	0,4130	0,4685	0,5160	0,5520	0,5830	0,6085	0,6255	0,6400	0,6500	0,6565	0,6600	0,6645	0,6665	0,6685
Pseudomonas aeruginosa	A03	5	2	irradiated group	0,0240	0,0285	0,0335	0,0400	0,0520	0,0720	0,0900	0,1165	0,1385	0,1770	0,2210	0,2670	0,3105	0,3510	0,3940	0,4620	0,5185	0,5585	0,5900	0,6140	0,6325	0,6450	0,6565	0,6625	0,6675
Pseudomonas aeruginosa	A04	5	2	control group	0,0170	0,0255	0,0405	0,0550	0,0720	0,0970	0,1260	0,1705	0,2245	0,2800	0,3310	0,3700	0,4405	0,5120	0,5690	0,6080	0,6365	0,6525	0,6650	0,6710	0,6745	0,6750	0,6775	0,6775	0,6765
Pseudomonas aeruginosa	C01	5	3	irradiated group	0,0370	0,0455	0,0615	0,0810	0,1030	0,1270	0,1690	0,2155	0,2675	0,3040	0,3420	0,3870	0,4545	0,5220	0,5670	0,5990	0,6245	0,6435	0,6580	0,6670	0,6725	0,6750	0,6785	0,6785	0,6785
Pseudomonas aeruginosa	C02	5	3	control group	0,0180	0,0225	0,0405	0,0540	0,0730	0,0960	0,1310	0,1835	0,2465	0,3090	0,3590	0,4070	0,4865	0,5540	0,6000	0,6310	0,6545	0,6655	0,6740	0,6780	0,6805	0,6800	0,6825	0,6805	0,6795
Pseudomonas aeruginosa	C03	5	4	irradiated group	0,0130	0,0205	0,0255	0,0350	0,0470	0,0640	0,0790	0,1015	0,1265	0,1720	0,2210	0,2750	0,3245	0,3640	0,4260	0,4960	0,5575	0,5995	0,6290	0,6480	0,6585	0,6640	0,6705	0,6715	0,6725
Pseudomonas aeruginosa	C04	5	4	control group	0,0180	0,0265	0,0445	0,0640	0,0800	0,1070	0,1450	0,1945	0,2555	0,3110	0,3610	0,4110	0,4845	0,5470	0,5890	0,6180	0,6395	0,6525	0,6640	0,6680	0,6725	0,6730	0,6765	0,6755	0,6755
Pseudomonas aeruginosa	A01	6	1	irradiated group	0,0385	0,0385	0,0520	0,0655	0,0755	0,0915	0,1165	0,1460	0,1745	0,1990	0,2460	0,3330	0,4160	0,4710	0,5340	0,5770	0,6095	0,6320	0,6490	0,6610	0,6700	0,6755	0,6805	0,6835	0,6860
Pseudomonas aeruginosa	A02	6	1	control group	0,0255	0,0265	0,0420	0,0555	0,0725	0,0955	0,1335	0,1900	0,2545	0,3150	0,3560	0,4350	0,5080	0,5660	0,6050	0,6310	0,6485	0,6590	0,6670	0,6730	0,6770	0,6805	0,6845	0,6865	0,6880
Pseudomonas aeruginosa	A03	6	2	irradiated group	0,0175	0,0215	0,0230	0,0275	0,0465	0,0625	0,0805	0,1080	0,1365	0,1650	0,1950	0,2170	0,2870	0,3650	0,4210	0,4760	0,5245	0,5620	0,5910	0,6110	0,6260	0,6355	0,6425	0,6475	0,6520
Pseudomonas aeruginosa	A04	6	2	control group	0,0205	0,0245	0,0420	0,0585	0,0765	0,1025	0,1425	0,1980	0,2605	0,3180	0,3650	0,4410	0,5140	0,5700	0,6080	0,6330	0,6495	0,6600	0,6670	0,6730	0,6770	0,6805	0,6835	0,6855	0,6870
Pseudomonas aeruginosa	C01	6	3	irradiated group	0,0445	0,0455	0,0620	0,0775	0,0985	0,1265	0,1605	0,2010	0,2425	0,2810	0,3440	0,4200	0,4790	0,5270	0,5680	0,5980	0,6235	0,6410	0,6540	0,6640	0,6710	0,6765	0,6815	0,6845	0,6870
Pseudomonas aeruginosa	C02	6	3	control group	0,0205	0,0235	0,0400	0,0575	0,0765	0,1015	0,1435	0,2040	0,2725	0,3330	0,3800	0,4590	0,5280	0,5800	0,6160	0,6400	0,6535	0,6630	0,6700	0,6750	0,6790	0,6815	0,6845	0,6875	0,6880
Pseudomonas aeruginosa	C03	6	4	irradiated group	0,0395	0,0465	0,0720	0,0915	0,1165	0,1515	0,1895	0,2310	0,2795	0,3270	0,3630	0,4200	0,4770	0,5250	0,5640	0,5930	0,6135	0,6300	0,6440	0,6540	0,6620	0,6685	0,6755	0,6805	0,6830
Pseudomonas aeruginosa	C04	6	4	control group	0,0215	0,0265	0,0440	0,0615	0,0815	0,1085	0,1525	0,2120	0,2755	0,3330	0,3760	0,4470	0,5190	0,5750	0,6120	0,6360	0,6505	0,6600	0,6680	0,6730	0,6770	0,6805	0,6835	0,6855	0,6870
Pseudomonas aeruginosa	A01	0	5	irradiated group	0,0075	0,0095	0,0155	0,0275	0,0485	0,0655	0,0865	0,1100	0,1415	0,1845	0,2335	0,2825	0,3265	0,3670	0,4080	0,4790	0,5450	0,5925	0,6230	0,6450	0,6595	0,6665	0,6715	0,6740	0,6740
Pseudomonas aeruginosa	A02	0	5	control group	0,0055	0,0075	0,0145	0,0285	0,0495	0,0685	0,0915	0,1170	0,1505	0,1955	0,2515	0,3035	0,3505	0,3920	0,4570	0,5320	0,5890	0,6245	0,6460	0,6600	0,6695	0,6735	0,6765	0,6780	0,6770
Pseudomonas aeruginosa	A03	0	6	irradiated group	0,0015	0,0065	0,0135	0,0265	0,0485	0,0675	0,0905	0,1160	0,1495	0,1945	0,2485	0,2995	0,3505	0,3950	0,4580	0,5310	0,5880	0,6245	0,6470	0,6600	0,6685	0,6725	0,6755	0,6770	0,6770
Pseudomonas aeruginosa	A04	0	6	control group	0,0025	0,0075	0,0165	0,0295	0,0515	0,0715	0,0935	0,1190	0,1535	0,1975	0,2505	0,3005	0,3485	0,3880	0,4450	0,5210	0,5820	0,6165	0,6400	0,6570	0,6665	0,6715	0,6735	0,6750	0,6750
Pseudomonas aeruginosa	C01	0	7	irradiated group	0,0085	0,0105	0,0165	0,0295	0,0515	0,0705	0,0925	0,1180	0,1515	0,1955	0,2465	0,2985	0,3435	0,3820	0,4330	0,5090	0,5720	0,6115	0,6370	0,6550	0,6665	0,6715	0,6745	0,6770	0,6760
Pseudomonas aeruginosa	C02	0	7	control group	0,0035	0,0075	0,0135	0,0285	0,0475	0,0665	0,0895	0,1160	0,1515	0,1975	0,2525	0,3035	0,3495	0,3930	0,4560	0,5300	0,5900	0,6255	0,6480	0,6610	0,6695	0,6735	0,6765	0,6780	0,6770
Pseudomonas aeruginosa	C03	0	8	irradiated group	0,0015	0,0055	0,0135	0,0275	0,0485	0,0705	0,0915	0,1180	0,1555	0,2005	0,2545	0,3105	0,3565	0,4000	0,4650	0,5370	0,5930	0,6285	0,6500	0,6620	0,6705	0,6735	0,6765	0,6770	0,6770
Pseudomonas aeruginosa	C04	0	8	control group	0,0015	0,0045	0,0125	0,0245	0,0445	0,0645	0,0855	0,1100	0,1435	0,1825	0,2335	0,2835	0,3315	0,3760	0,4230	0,4950	0,5630	0,6035	0,6310	0,6490	0,6615	0,6685	0,6715	0,6740	0,6740
Pseudomonas aeruginosa	A01	1	5	irradiated group	0,0225	0,0275	0,0300	0,0370	0,0495	0,0690	0,0875	0,1100	0,1380	0,1785	0,2300	0,2825	0,3310	0,3755	0,4235	0,4930	0,5525	0,5950	0,6250	0,6465	0,6600	0,6680	0,6750	0,6780	0,6795
Pseudomonas aeruginosa	A02	1	5	control group	0,0235	0,0295	0,0450	0,0640	0,0815	0,1100	0,1485	0,1970	0,2600	0,3225	0,3710	0,4275	0,4970	0,5625	0,6055	0,6320	0,6485	0,6600	0,6670	0,6725	0,6770	0,6790	0,6820	0,6840	0,6835
Pseudomonas aeruginosa	A03	1	6	irradiated group	0,0225	0,0285	0,0340	0,0470	0,0615	0,0790	0,0995	0,1250	0,1600	0,2055	0,2640	0,3195	0,3660	0,4195	0,4955	0,5600	0,6055	0,6330	0,6500	0,6625	0,6710	0,6760	0,6800	0,6820	0,6815
Pseudomonas aeruginosa	A04	1	6	control group	0,0185	0,0265	0,0460	0,0670	0,0885	0,1180	0,1595	0,2120	0,2730	0,3315	0,3860	0,4445	0,5100	0,5705	0,6085	0,6340	0,6495	0,6620	0,6670	0,6715	0,6770	0,6790	0,6820	0,6840	0,6835
Pseudomonas aeruginosa	C01	1	7	irradiated group	0,0285	0,0305	0,0390	0,0570	0,0745	0,0990	0,1275	0,1610	0,2080	0,2575	0,3200	0,3745	0,4230	0,4765	0,5425	0,5890	0,6195	0,6420	0,6550	0,6655	0,6730	0,6770	0,6810	0,6830	0,6825
Pseudomonas aeruginosa	C02	1	7	control group	0,0205	0,0275	0,0440	0,0630	0,0845	0,1130	0,1515	0,2050	0,2700	0,3305	0,3840	0,4395	0,5080	0,5675	0,6115	0,6360	0,6525	0,6630	0,6680	0,6735	0,6780	0,6810	0,6830	0,6850	0,6845
Pseudomonas aeruginosa	C03	1	8	irradiated group	0,0125	0,0185	0,0220	0,0290	0,0425	0,0600	0,0775	0,1010	0,1280	0,1685	0,2220	0,2775	0,3290	0,3765	0,4465	0,5200	0,5765	0,6200	0,6410	0,6575	0,6680	0,6730	0,6780	0,6810	0,6805
Pseudomonas aeruginosa	C04	1	8	control group	0,0245	0,0345	0,0560	0,0770	0,1045	0,1390	0,1875	0,2450	0,3110	0,3695	0,4210	0,4785	0,5400	0,5895	0,6215	0,6430	0,6545	0,6650	0,6690	0,6735	0,6780	0,6800	0,6830	0,6840	0,6835
Pseudomonas aeruginosa	A01	2	5	irradiated group	0,0280	0,0355	0,0395	0,0530	0,0710	0,0925	0,1140	0,1455	0,1835	0,2340	0,2930	0,3420	0,3895	0,4315	0,4880	0,5470	0,5915	0,6230	0,6435	0,6570	0,6640	0,6690	0,6720	0,6755	0,6755
Pseudomonas aeruginosa	A02	2	5	control group	0,0350	0,0485	0,0655	0,0880	0,1220	0,1645	0,2160	0,2835	0,3495	0,4040	0,4490	0,5120	0,5705	0,6135	0,6390	0,6540	0,6615	0,6680	0,6735	0,6760	0,6770	0,6780	0,6780	0,6795	0,6795
Pseudomonas aeruginosa	A03	2	6	irradiated group	0,0590	0,0695	0,0835	0,1020	0,1330	0,1605	0,2050	0,2635	0,3245	0,3790	0,4270	0,4920	0,5585	0,6075	0,6330	0,6500	0,6605	0,6670	0,6725	0,6760	0,6770	0,6780	0,6780	0,6805	0,6795
Pseudomonas aeruginosa	A04	2	6	control group	0,0270	0,0365	0,0555	0,0800	0,1080	0,1465	0,1980	0,2545	0,3185	0,3760	0,4260	0,4850	0,5455	0,5945	0,6290	0,6480	0,6585	0,6650	0,6705	0,6730	0,6750	0,6770	0,6770	0,6785	0,6775
Pseudomonas aeruginosa	C01	2	7	irradiated group	0,0260	0,0275	0,0315	0,0320	0,0370	0,0485	0,0630	0,0795	0,0975	0,1180	0,1490	0,1840	0,2245	0,2645	0,3000	0,3350	0,3675	0,3980	0,4515	0,5040	0,5460	0,5790	0,6050	0,6255	0,6385
Pseudomonas aeruginosa	C02	2	7	control group	0,0310	0,0425	0,0605	0,0830	0,1150	0,1515	0,1980	0,2615	0,3295	0,3910	0,4350	0,4950	0,5555	0,6045	0,6330	0,6510	0,6605	0,6670	0,6725	0,6750	0,6760	0,6770	0,6770	0,6795	0,6775
Pseudomonas aeruginosa	C03	2	8	irradiated group	0,0320	0,0415	0,0575	0,0760	0,0990	0,1275	0,1660	0,2165	0,2755	0,3360	0,3960	0,4510	0,5095	0,5685	0,6100	0,6390	0,6535	0,6630	0,6695	0,6740	0,6760	0,6770	0,6780	0,6795	0,6795
Pseudomonas aeruginosa	C04	2	8	control group	0,0260	0,0395	0,0555	0,0790	0,1090	0,1445	0,1880	0,2475	0,3125	0,3750	0,4230	0,4750	0,5355	0,5895	0,6250	0,6460	0,6575	0,6640	0,6705	0,6740	0,6750	0,6760	0,6770	0,6795	0,6785
Pseudomonas aeruginosa	A01	3	5	irradiated group	0,0245	0,0280	0,0320	0,0335	0,0450	0,0580	0,0735	0,0910	0,1130	0,1545	0,1920	0,2415	0,2880	0,3320	0,3725	0,4355	0,5135	0,5685	0,6105	0,6380	0,6585	0,6715	0,6810	0,6835	0,6860
Pseudomonas aeruginosa	A02	3	5	control group	0,0295	0,0360	0,0510	0,0675	0,0910	0,1130	0,1425	0,1840	0,2360	0,2865	0,3310	0,3665	0,4000	0,4450	0,4935	0,5375	0,5755	0,6045	0,6265	0,6430	0,6565	0,6675	0,6760	0,6785	0,6810
Pseudomonas aeruginosa	A03	3	6	irradiated group	0,0295	0,0320	0,0360	0,0465	0,0660	0,0870	0,1085	0,1370	0,1750	0,2345	0,2840	0,3365	0,3800	0,4210	0,4825	0,5365	0,5805	0,6135	0,6375	0,6540	0,6675	0,6765	0,6840	0,6855	0,6870
Pseudomonas aeruginosa	A04	3	6	control group	0,0225	0,0300	0,0460	0,0645	0,0860	0,1110	0,1445	0,1910	0,2380	0,2915	0,3330	0,3655	0,4010	0,4500	0,5035	0,5485	0,5855	0,6115	0,6325	0,6480	0,6605	0,6705	0,6780	0,6805	0,6820
Pseudomonas aeruginosa	C01	3	7	irradiated group	0,0135	0,0200	0,0210	0,0235	0,0260	0,0320	0,0395	0,0560	0,0690	0,0905	0,1110	0,1305	0,1640	0,2050	0,2455	0,2865	0,3255	0,3565	0,3895	0,4450	0,5065	0,5585	0,5990	0,6245	0,6440
Pseudomonas aeruginosa	C02	3	7	control group	0,0285	0,0350	0,0470	0,0645	0,0870	0,1070	0,1375	0,1770	0,2260	0,2705	0,3070	0,3425	0,3760	0,4100	0,4535	0,5005	0,5425	0,5775	0,6065	0,6280	0,6455	0,6585	0,6700	0,6745	0,6780
Pseudomonas aeruginosa	C03	3	8	irradiated group	0,0335	0,0420	0,0500	0,0685	0,0910	0,1190	0,1525	0,1970	0,2520	0,3145	0,3780	0,4305	0,4820	0,5310	0,5685	0,5955	0,6185	0,6365	0,6505	0,6600	0,6695	0,6765	0,6820	0,6835	0,6850
Pseudomonas aeruginosa	C04	3	8	control group	0,0295	0,0380	0,0530	0,0745	0,1010	0,1310	0,1695	0,2210	0,2740	0,3225	0,3590	0,3895	0,4280	0,4720	0,5165	0,5565	0,5905	0,6135	0,6335	0,6480	0,6605	0,6705	0,6780	0,6795	0,6810
Pseudomonas aeruginosa	A01	4	5	irradiated group	0,0370	0,0395	0,0440	0,0530	0,0670	0,0810	0,0980	0,1190	0,1405	0,1725	0,2080	0,2515	0,2925	0,3325	0,3705	0,4045	0,4505	0,5015	0,5490	0,5835	0,6105	0,6285	0,6430	0,6540	0,6605
Pseudomonas aeruginosa	A02	4	5	control group	0,0210	0,0235	0,0340	0,0460	0,0590	0,0730	0,0920	0,1180	0,1545	0,2015	0,2500	0,2985	0,3385	0,3825	0,4455	0,5075	0,5575	0,5965	0,6220	0,6395	0,6525	0,6615	0,6680	0,6730	0,6745
Pseudomonas aeruginosa	A03	4	6	irradiated group	0,0190	0,0205	0,0210	0,0230	0,0240	0,0250	0,0300	0,0400	0,0515	0,0635	0,0780	0,0955	0,1245	0,1565	0,1955	0,2345	0,2685	0,2985	0,3680	0,4435	0,5055	0,5505	0,5840	0,6090	0,6255
Pseudomonas aeruginosa	A04	4	6	control group	0,0140	0,0185	0,0290	0,0440	0,0550	0,0680	0,0870	0,1120	0,1475	0,1915	0,2390	0,2845	0,3275	0,3675	0,4365	0,4985	0,5525	0,5915	0,6200	0,6385	0,6515	0,6605	0,6670	0,6720	0,6745
Pseudomonas aeruginosa	C01	4	7	irradiated group	0,0170	0,0185	0,0230	0,0260	0,0340	0,0450	0,0570	0,0700	0,0845	0,1035	0,1280	0,1615	0,2065	0,2475	0,2845	0,3195	0,3495	0,4065	0,4710	0,5285	0,5725	0,6035	0,6270	0,6440	0,6535
Pseudomonas aeruginosa	C02	4	7	control group	0,0190	0,0215	0,0300	0,0450	0,0580	0,0720	0,0910	0,1190	0,1555	0,2025	0,2540	0,3045	0,3465	0,3945	0,4635	0,5275	0,5755	0,6105	0,6340	0,6495	0,6605	0,6675	0,6730	0,6770	0,6775
Pseudomonas aeruginosa	C03	4	8	irradiated group	0,0130	0,0185	0,0190	0,0230	0,0270	0,0340	0,0480	0,0600	0,0755	0,0925	0,1160	0,1485	0,1935	0,2365	0,2785	0,3155	0,3655	0,4475	0,5150	0,5645	0,6005	0,6235	0,6410	0,6530	0,6605
Pseudomonas aeruginosa	C04	4	8	control group	0,0140	0,0205	0,0310	0,0470	0,0600	0,0750	0,0930	0,1210	0,1575	0,2035	0,2530	0,2985	0,3435	0,3805	0,4405	0,5035	0,5565	0,5945	0,6210	0,6385	0,6525	0,6615	0,6670	0,6720	0,6735
Pseudomonas aeruginosa	A01	5	5	irradiated group	0,0255	0,0325	0,0370	0,0455	0,0615	0,0715	0,0940	0,1110	0,1395	0,1695	0,2115	0,2580	0,2995	0,3350	0,3695	0,4240	0,4915	0,5435	0,5850	0,6130	0,6340	0,6490	0,6585	0,6645	0,6685
Pseudomonas aeruginosa	A02	5	5	control group	0,0195	0,0215	0,0330	0,0465	0,0605	0,0765	0,0990	0,1290	0,1715	0,2245	0,2775	0,3200	0,3625	0,4300	0,4985	0,5550	0,5965	0,6255	0,6460	0,6610	0,6700	0,6770	0,6795	0,6805	0,6815
Pseudomonas aeruginosa	A03	5	6	irradiated group	0,0085	0,0105	0,0150	0,0195	0,0285	0,0395	0,0520	0,0630	0,0785	0,1025	0,1325	0,1670	0,2025	0,2310	0,2605	0,3220	0,4025	0,4685	0,5170	0,5530	0,5790	0,6000	0,6135	0,6275	0,6375
Pseudomonas aeruginosa	A04	5	6	control group	0,0145	0,0185	0,0330	0,0465	0,0595	0,0765	0,0990	0,1300	0,1695	0,2185	0,2655	0,3130	0,3555	0,4230	0,4915	0,5510	0,5925	0,6225	0,6440	0,6600	0,6690	0,6750	0,6775	0,6795	0,6795
Pseudomonas aeruginosa	C01	5	7	irradiated group	0,0115	0,0115	0,0140	0,0155	0,0175	0,0195	0,0290	0,0400	0,0535	0,0695	0,0855	0,1070	0,1395	0,1800	0,2255	0,2670	0,3005	0,3305	0,3900	0,4630	0,5260	0,5730	0,6065	0,6275	0,6435
Pseudomonas aeruginosa	C02	5	7	control group	0,0245	0,0275	0,0460	0,0635	0,0845	0,1105	0,1400	0,1810	0,2345	0,2935	0,3425	0,3870	0,4485	0,5110	0,5645	0,6060	0,6325	0,6515	0,6650	0,6750	0,6800	0,6840	0,6845	0,6845	0,6845
Pseudomonas aeruginosa	C03	5	8	irradiated group	0,0095	0,0145	0,0150	0,0205	0,0255	0,0355	0,0500	0,0650	0,0795	0,1045	0,1395	0,1720	0,2115	0,2420	0,2835	0,3680	0,4495	0,5105	0,5550	0,5880	0,6110	0,6280	0,6415	0,6505	0,6585
Pseudomonas aeruginosa	C04	5	8	control group	0,0185	0,0245	0,0420	0,0565	0,0715	0,0925	0,1190	0,1590	0,2095	0,2605	0,3105	0,3540	0,3985	0,4660	0,5295	0,5800	0,6145	0,6385	0,6550	0,6670	0,6740	0,6780	0,6805	0,6815	0,6805
Pseudomonas aeruginosa	A01	6	5	irradiated group	0,0180	0,0195	0,0240	0,0290	0,0385	0,0390	0,0560	0,0695	0,0885	0,1010	0,1250	0,1600	0,1950	0,2275	0,2635	0,2925	0,3305	0,3950	0,4550	0,5040	0,5430	0,5725	0,5950	0,6115	0,6250
Pseudomonas aeruginosa	A02	6	5	control group	0,0210	0,0235	0,0400	0,0510	0,0695	0,0860	0,1140	0,1505	0,2015	0,2580	0,3110	0,3500	0,4130	0,4815	0,5425	0,5835	0,6125	0,6320	0,6440	0,6520	0,6600	0,6655	0,6680	0,6695	0,6720
Pseudomonas aeruginosa	A03	6	6	irradiated group	0,0070	0,0105	0,0130	0,0140	0,0155	0,0170	0,0200	0,0265	0,0395	0,0520	0,0620	0,0760	0,1000	0,1305	0,1595	0,1855	0,2075	0,2620	0,3390	0,4010	0,4550	0,4955	0,5270	0,5505	0,5690
Pseudomonas aeruginosa	A04	6	6	control group	0,0190	0,0225	0,0400	0,0540	0,0725	0,0900	0,1160	0,1545	0,2055	0,2600	0,3070	0,3490	0,4060	0,4775	0,5365	0,5805	0,6105	0,6300	0,6430	0,6510	0,6590	0,6645	0,6680	0,6695	0,6710
Pseudomonas aeruginosa	C01	6	7	irradiated group	0,0100	0,0135	0,0170	0,0170	0,0175	0,0200	0,0250	0,0345	0,0505	0,0650	0,0810	0,1000	0,1280	0,1675	0,2165	0,2615	0,2965	0,3260	0,3720	0,4420	0,5100	0,5605	0,5970	0,6195	0,6340
Pseudomonas aeruginosa	C02	6	7	control group	0,0200	0,0255	0,0410	0,0590	0,0775	0,0980	0,1270	0,1675	0,2205	0,2750	0,3280	0,3670	0,4400	0,5095	0,5645	0,6005	0,6245	0,6400	0,6500	0,6560	0,6620	0,6665	0,6690	0,6705	0,6720
Pseudomonas aeruginosa	C03	6	8	irradiated group	0,0250	0,0355	0,0450	0,0570	0,0705	0,0890	0,1170	0,1465	0,1885	0,2200	0,2590	0,3360	0,4160	0,4785	0,5245	0,5575	0,5805	0,6010	0,6180	0,6310	0,6450	0,6545	0,6610	0,6655	0,6690
Pseudomonas aeruginosa	C04	6	8	control group	0,0230	0,0295	0,0480	0,0700	0,0925	0,1110	0,1420	0,1825	0,2335	0,2870	0,3330	0,3710	0,4270	0,4955	0,5495	0,5875	0,6135	0,6330	0,6450	0,6520	0,6600	0,6635	0,6670	0,6685	0,6700

				Messzeitpunkt	0 h	0 h 30 min	1 h	1 h 30 min	2 h	2 h 30 min	3 h	3 h 30 min	4 h	4 h 30 min	5 h	5 h 30 min	6 h	6 h 30 min	7 h	7 h 30 min	8 h	8 h 30 min	9 h	9 h 30 min	10 h	10 h 30 min	11 h	11 h 30 min	12 h
Erreger	Position - 12-Well-Mikrotiterplatte	Stage	Stichprobenanzahl	Versuchsgruppe	Blank corrected based on Raw Data (600)	Blank corrected based on Raw Data (600)	Blank corrected based on Raw Data (600)	Blank corrected based on Raw Data (600)	Blank corrected based on Raw Data (600)	Blank corrected based on Raw Data (600)	Blank corrected based on Raw Data (600)	Blank corrected based on Raw Data (600)	Blank corrected based on Raw Data (600)	Blank corrected based on Raw Data (600)	Blank corrected based on Raw Data (600)	Blank corrected based on Raw Data (600)	Blank corrected based on Raw Data (600)	Blank corrected based on Raw Data (600)	Blank corrected based on Raw Data (600)	Blank corrected based on Raw Data (600)	Blank corrected based on Raw Data (600)	Blank corrected based on Raw Data (600)	Blank corrected based on Raw Data (600)	Blank corrected based on Raw Data (600)	Blank corrected based on Raw Data (600)	Blank corrected based on Raw Data (600)	Blank corrected based on Raw Data (600)	Blank corrected based on Raw Data (600)	Blank corrected based on Raw Data (600)
Staphylococcus aureus	A01	0	1	irradiated group	0,0085	0,0165	0,0275	0,0555	0,0950	0,1420	0,1980	0,2740	0,3595	0,4360	0,4815	0,5020	0,5120	0,5175	0,5180	0,5140	0,5140	0,5095	0,5115	0,5160	0,5180	0,5180	0,5235	0,5235	0,5280
Staphylococcus aureus	A02	0	1	control group	0,0085	0,0165	0,0285	0,0565	0,0970	0,1460	0,2030	0,2750	0,3625	0,4370	0,4885	0,5110	0,5200	0,5265	0,5290	0,5290	0,5260	0,5225	0,5225	0,5280	0,5280	0,5310	0,5305	0,5315	0,5340
Staphylococcus aureus	A03	0	2	irradiated group	0,0065	0,0155	0,0285	0,0595	0,1020	0,1540	0,2110	0,2830	0,3675	0,4410	0,4905	0,5100	0,5210	0,5275	0,5290	0,5290	0,5250	0,5215	0,5215	0,5270	0,5280	0,5310	0,5305	0,5335	0,5350
Staphylococcus aureus	A04	0	2	control group	0,0065	0,0175	0,0325	0,0655	0,1110	0,1640	0,2240	0,2950	0,3765	0,4480	0,4935	0,5140	0,5220	0,5285	0,5300	0,5300	0,5270	0,5255	0,5265	0,5320	0,5330	0,5350	0,5345	0,5375	0,5390
Staphylococcus aureus	C01	0	3	irradiated group	0,0085	0,0165	0,0265	0,0545	0,0960	0,1460	0,2030	0,2760	0,3585	0,4340	0,4825	0,5070	0,5170	0,5235	0,5250	0,5250	0,5230	0,5225	0,5235	0,5280	0,5320	0,5320	0,5325	0,5335	0,5340
Staphylococcus aureus	C02	0	3	control group	0,0065	0,0135	0,0265	0,0535	0,0940	0,1430	0,2000	0,2710	0,3555	0,4300	0,4815	0,5040	0,5150	0,5215	0,5240	0,5250	0,5240	0,5225	0,5255	0,5310	0,5330	0,5350	0,5345	0,5375	0,5390
Staphylococcus aureus	C03	0	4	irradiated group	0,0025	0,0135	0,0275	0,0555	0,0990	0,1510	0,2070	0,2780	0,3615	0,4360	0,4855	0,5060	0,5150	0,5215	0,5240	0,5250	0,5240	0,5235	0,5265	0,5340	0,5360	0,5390	0,5375	0,5395	0,5410
Staphylococcus aureus	C04	0	4	control group	0,0075	0,0165	0,0325	0,0635	0,1090	0,1600	0,2180	0,2890	0,3685	0,4420	0,4865	0,5060	0,5160	0,5205	0,5210	0,5210	0,5210	0,5215	0,5245	0,5320	0,5350	0,5370	0,5375	0,5395	0,5420
Staphylococcus aureus	A01	1	1	irradiated group	0,0070	0,0120	0,0250	0,0505	0,0940	0,1475	0,2065	0,2800	0,3580	0,4245	0,4710	0,5005	0,5155	0,5230	0,5290	0,5300	0,5320	0,5345	0,5395	0,5455	0,5500	0,5530	0,5560	0,5585	0,5595
Staphylococcus aureus	A02	1	1	control group	0,0030	0,0060	0,0150	0,0365	0,0830	0,1405	0,2025	0,2770	0,3550	0,4255	0,4760	0,5045	0,5205	0,5300	0,5360	0,5380	0,5410	0,5445	0,5495	0,5555	0,5590	0,5620	0,5640	0,5675	0,5675
Staphylococcus aureus	A03	1	2	irradiated group	0,0020	0,0090	0,0190	0,0395	0,0750	0,1235	0,1775	0,2380	0,3150	0,3875	0,4530	0,4925	0,5125	0,5230	0,5310	0,5340	0,5350	0,5375	0,5425	0,5465	0,5500	0,5560	0,5580	0,5615	0,5615
Staphylococcus aureus	A04	1	2	control group	0,0040	0,0100	0,0220	0,0525	0,1080	0,1685	0,2335	0,3110	0,3820	0,4505	0,4910	0,5165	0,5285	0,5350	0,5400	0,5430	0,5450	0,5485	0,5535	0,5585	0,5620	0,5650	0,5660	0,5685	0,5685
Staphylococcus aureus	C01	1	3	irradiated group	0,0060	0,0120	0,0240	0,0485	0,0890	0,1425	0,1995	0,2670	0,3460	0,4125	0,4650	0,4995	0,5165	0,5260	0,5330	0,5350	0,5370	0,5395	0,5445	0,5485	0,5520	0,5580	0,5590	0,5625	0,5635
Staphylococcus aureus	C02	1	3	control group	0,0040	0,0100	0,0240	0,0555	0,1090	0,1715	0,2415	0,3210	0,3920	0,4555	0,4940	0,5175	0,5305	0,5380	0,5410	0,5430	0,5460	0,5495	0,5545	0,5605	0,5630	0,5660	0,5680	0,5705	0,5705
Staphylococcus aureus	C03	1	4	irradiated group	0,0020	0,0090	0,0190	0,0405	0,0790	0,1285	0,1825	0,2480	0,3250	0,3955	0,4550	0,4935	0,5135	0,5240	0,5310	0,5340	0,5350	0,5385	0,5425	0,5465	0,5510	0,5570	0,5590	0,5625	0,5625
Staphylococcus aureus	C04	1	4	control group	0,0030	0,0120	0,0250	0,0575	0,1160	0,1765	0,2415	0,3190	0,3890	0,4535	0,4920	0,5155	0,5275	0,5350	0,5390	0,5410	0,5440	0,5475	0,5525	0,5575	0,5610	0,5640	0,5650	0,5685	0,5685
Staphylococcus aureus	A01	2	1	irradiated group	0,0135	0,0175	0,0320	0,0665	0,1160	0,1725	0,2380	0,3165	0,3885	0,4445	0,4850	0,5090	0,5225	0,5305	0,5330	0,5365	0,5380	0,5410	0,5470	0,5485	0,5515	0,5530	0,5550	0,5550	0,5540
Staphylococcus aureus	A02	2	1	control group	0,0105	0,0155	0,0280	0,0645	0,1170	0,1745	0,2480	0,3275	0,4015	0,4605	0,4980	0,5200	0,5325	0,5395	0,5420	0,5455	0,5480	0,5520	0,5570	0,5580	0,5595	0,5620	0,5630	0,5630	0,5620
Staphylococcus aureus	A03	2	2	irradiated group	0,0085	0,0155	0,0300	0,0615	0,1120	0,1625	0,2250	0,2985	0,3755	0,4405	0,4820	0,5090	0,5235	0,5315	0,5370	0,5385	0,5400	0,5430	0,5470	0,5505	0,5525	0,5550	0,5560	0,5560	0,5560
Staphylococcus aureus	A04	2	2	control group	0,0085	0,0165	0,0330	0,0765	0,1360	0,1935	0,2670	0,3485	0,4175	0,4705	0,5040	0,5240	0,5345	0,5415	0,5440	0,5465	0,5490	0,5520	0,5570	0,5575	0,5595	0,5610	0,5620	0,5620	0,5610
Staphylococcus aureus	C01	2	3	irradiated group	0,0125	0,0195	0,0330	0,0685	0,1160	0,1735	0,2350	0,3115	0,3845	0,4425	0,4840	0,5100	0,5245	0,5325	0,5380	0,5395	0,5410	0,5440	0,5480	0,5515	0,5545	0,5560	0,5570	0,5580	0,5570
Staphylococcus aureus	C02	2	3	control group	0,0105	0,0165	0,0290	0,0675	0,1210	0,1825	0,2540	0,3375	0,4085	0,4645	0,5000	0,5220	0,5335	0,5415	0,5430	0,5465	0,5480	0,5520	0,5560	0,5585	0,5605	0,5620	0,5630	0,5630	0,5620
Staphylococcus aureus	C03	2	4	irradiated group	0,0075	0,0145	0,0300	0,0635	0,1160	0,1735	0,2370	0,3145	0,3885	0,4475	0,4870	0,5120	0,5255	0,5335	0,5370	0,5395	0,5400	0,5440	0,5480	0,5505	0,5535	0,5550	0,5550	0,5560	0,5550
Staphylococcus aureus	C04	2	4	control group	0,0105	0,0175	0,0340	0,0755	0,1340	0,1935	0,2650	0,3455	0,4135	0,4675	0,5010	0,5210	0,5335	0,5395	0,5420	0,5445	0,5470	0,5500	0,5540	0,5555	0,5585	0,5600	0,5610	0,5610	0,5600
Staphylococcus aureus	A01	3	1	irradiated group	0,0135	0,0180	0,0350	0,0740	0,1280	0,1850	0,2570	0,3395	0,4075	0,4600	0,4980	0,5220	0,5345	0,5435	0,5455	0,5505	0,5550	0,5590	0,5630	0,5675	0,5695	0,5720	0,5735	0,5750	0,5750
Staphylococcus aureus	A02	3	1	control group	0,0115	0,0150	0,0310	0,0690	0,1220	0,1780	0,2490	0,3335	0,4065	0,4620	0,5000	0,5240	0,5365	0,5465	0,5485	0,5535	0,5580	0,5630	0,5670	0,5705	0,5725	0,5750	0,5775	0,5780	0,5780
Staphylococcus aureus	A03	3	2	irradiated group	0,0095	0,0160	0,0380	0,0790	0,1330	0,1920	0,2630	0,3455	0,4165	0,4700	0,5050	0,5270	0,5385	0,5475	0,5505	0,5545	0,5590	0,5640	0,5680	0,5705	0,5715	0,5750	0,5765	0,5770	0,5770
Staphylococcus aureus	A04	3	2	control group	0,0105	0,0170	0,0360	0,0800	0,1380	0,1950	0,2670	0,3485	0,4185	0,4730	0,5080	0,5300	0,5405	0,5495	0,5515	0,5555	0,5620	0,5660	0,5700	0,5725	0,5735	0,5770	0,5785	0,5790	0,5790
Staphylococcus aureus	C01	3	3	irradiated group	0,0105	0,0160	0,0350	0,0730	0,1270	0,1840	0,2540	0,3355	0,4065	0,4590	0,4990	0,5230	0,5365	0,5465	0,5495	0,5535	0,5580	0,5620	0,5660	0,5705	0,5715	0,5750	0,5765	0,5770	0,5770
Staphylococcus aureus	C02	3	3	control group	0,0095	0,0140	0,0320	0,0710	0,1260	0,1850	0,2560	0,3385	0,4095	0,4660	0,5030	0,5280	0,5405	0,5495	0,5515	0,5565	0,5610	0,5660	0,5700	0,5725	0,5745	0,5780	0,5795	0,5800	0,5800
Staphylococcus aureus	C03	3	4	irradiated group	0,0055	0,0160	0,0350	0,0770	0,1330	0,1910	0,2610	0,3425	0,4105	0,4650	0,5030	0,5250	0,5365	0,5455	0,5485	0,5525	0,5570	0,5610	0,5660	0,5685	0,5705	0,5740	0,5755	0,5760	0,5760
Staphylococcus aureus	C04	3	4	control group	0,0095	0,0160	0,0370	0,0800	0,1380	0,1970	0,2660	0,3475	0,4155	0,4700	0,5060	0,5280	0,5395	0,5485	0,5505	0,5545	0,5600	0,5640	0,5690	0,5715	0,5725	0,5760	0,5775	0,5780	0,5780
Staphylococcus aureus	A01	4	1	irradiated group	0,0170	0,0220	0,0410	0,0800	0,1330	0,1895	0,2620	0,3450	0,4125	0,4650	0,5030	0,5255	0,5380	0,5460	0,5505	0,5520	0,5560	0,5595	0,5640	0,5645	0,5655	0,5655	0,5645	0,5635	0,5635
Staphylococcus aureus	A02	4	1	control group	0,0080	0,0120	0,0270	0,0640	0,1150	0,1715	0,2410	0,3250	0,3965	0,4560	0,4950	0,5185	0,5330	0,5420	0,5455	0,5490	0,5530	0,5565	0,5600	0,5625	0,5635	0,5635	0,5625	0,5625	0,5625
Staphylococcus aureus	A03	4	2	irradiated group	0,0080	0,0150	0,0320	0,0700	0,1220	0,1775	0,2410	0,3200	0,3945	0,4580	0,4980	0,5235	0,5380	0,5470	0,5535	0,5550	0,5580	0,5605	0,5640	0,5665	0,5675	0,5675	0,5665	0,5655	0,5655
Staphylococcus aureus	A04	4	2	control group	0,0050	0,0130	0,0300	0,0710	0,1280	0,1855	0,2540	0,3380	0,4085	0,4650	0,5020	0,5225	0,5360	0,5440	0,5475	0,5510	0,5540	0,5575	0,5620	0,5625	0,5645	0,5625	0,5625	0,5625	0,5615
Staphylococcus aureus	C01	4	3	irradiated group	0,0110	0,0180	0,0350	0,0730	0,1230	0,1805	0,2450	0,3240	0,3985	0,4560	0,4980	0,5225	0,5360	0,5460	0,5525	0,5540	0,5570	0,5595	0,5630	0,5665	0,5675	0,5665	0,5655	0,5655	0,5655
Staphylococcus aureus	C02	4	3	control group	0,0090	0,0140	0,0310	0,0690	0,1240	0,1825	0,2530	0,3360	0,4085	0,4660	0,5050	0,5285	0,5420	0,5510	0,5545	0,5570	0,5610	0,5635	0,5670	0,5685	0,5705	0,5695	0,5685	0,5685	0,5675
Staphylococcus aureus	C03	4	4	irradiated group	0,0070	0,0160	0,0320	0,0690	0,1210	0,1755	0,2400	0,3180	0,3915	0,4550	0,4960	0,5205	0,5360	0,5450	0,5505	0,5520	0,5560	0,5585	0,5620	0,5645	0,5655	0,5645	0,5645	0,5635	0,5635
Staphylococcus aureus	C04	4	4	control group	0,0100	0,0180	0,0360	0,0810	0,1400	0,1985	0,2690	0,3500	0,4175	0,4730	0,5100	0,5305	0,5430	0,5510	0,5555	0,5580	0,5610	0,5645	0,5680	0,5685	0,5705	0,5685	0,5685	0,5675	0,5665
Staphylococcus aureus	A01	5	1	irradiated group	0,0160	0,0220	0,0365	0,0730	0,1230	0,1800	0,2485	0,3280	0,3980	0,4540	0,4915	0,5155	0,5285	0,5370	0,5400	0,5440	0,5485	0,5530	0,5580	0,5625	0,5660	0,5675	0,5695	0,5705	0,5700
Staphylococcus aureus	A02	5	1	control group	0,0110	0,0170	0,0305	0,0700	0,1210	0,1780	0,2475	0,3310	0,4040	0,4640	0,5005	0,5245	0,5365	0,5470	0,5490	0,5550	0,5585	0,5630	0,5690	0,5715	0,5740	0,5765	0,5775	0,5795	0,5770
Staphylococcus aureus	A03	5	2	irradiated group	0,0100	0,0180	0,0335	0,0680	0,1170	0,1710	0,2335	0,3090	0,3840	0,4480	0,4885	0,5155	0,5285	0,5380	0,5450	0,5480	0,5515	0,5550	0,5600	0,5645	0,5670	0,5705	0,5715	0,5725	0,5710
Staphylococcus aureus	A04	5	2	control group	0,0110	0,0180	0,0345	0,0790	0,1350	0,1930	0,2615	0,3440	0,4150	0,4710	0,5065	0,5285	0,5395	0,5490	0,5520	0,5570	0,5605	0,5660	0,5710	0,5725	0,5750	0,5775	0,5785	0,5805	0,5780
Staphylococcus aureus	C01	5	3	irradiated group	0,0140	0,0200	0,0345	0,0660	0,1140	0,1690	0,2325	0,3080	0,3810	0,4420	0,4845	0,5125	0,5255	0,5350	0,5410	0,5450	0,5475	0,5520	0,5570	0,5615	0,5660	0,5675	0,5695	0,5715	0,5700
Staphylococcus aureus	C02	5	3	control group	0,0080	0,0140	0,0295	0,0680	0,1190	0,1790	0,2475	0,3300	0,4030	0,4630	0,5015	0,5255	0,5375	0,5480	0,5510	0,5560	0,5595	0,5630	0,5700	0,5725	0,5750	0,5775	0,5795	0,5795	0,5780
Staphylococcus aureus	C03	5	4	irradiated group	0,0080	0,0180	0,0335	0,0740	0,1230	0,1810	0,2485	0,3260	0,3970	0,4560	0,4955	0,5185	0,5305	0,5400	0,5450	0,5480	0,5515	0,5560	0,5610	0,5655	0,5680	0,5705	0,5725	0,5735	0,5710
Staphylococcus aureus	C04	5	4	control group	0,0100	0,0170	0,0355	0,0790	0,1340	0,1920	0,2625	0,3450	0,4120	0,4690	0,5055	0,5255	0,5355	0,5460	0,5490	0,5530	0,5565	0,5610	0,5670	0,5705	0,5720	0,5745	0,5765	0,5775	0,5750
Staphylococcus aureus	A01	6	1	irradiated group	0,0175	0,0210	0,0355	0,0750	0,1260	0,1815	0,2480	0,3270	0,4030	0,4630	0,5055	0,5330	0,5520	0,5645	0,5685	0,5740	0,5810	0,5895	0,5965	0,6015	0,6050	0,6085	0,6110	0,6145	0,6150
Staphylococcus aureus	A02	6	1	control group	0,0085	0,0130	0,0265	0,0640	0,1130	0,1685	0,2360	0,3180	0,3930	0,4560	0,4995	0,5280	0,5460	0,5585	0,5625	0,5700	0,5770	0,5855	0,5935	0,5985	0,6010	0,6045	0,6080	0,6115	0,6120
Staphylococcus aureus	A03	6	2	irradiated group	0,0075	0,0140	0,0305	0,0670	0,1160	0,1705	0,2310	0,3060	0,3840	0,4520	0,4985	0,5300	0,5490	0,5625	0,5695	0,5750	0,5820	0,5895	0,5955	0,6025	0,6050	0,6085	0,6120	0,6155	0,6160
Staphylococcus aureus	A04	6	2	control group	0,0105	0,0170	0,0325	0,0760	0,1320	0,1915	0,2580	0,3390	0,4130	0,4750	0,5145	0,5410	0,5580	0,5705	0,5745	0,5810	0,5880	0,5975	0,6035	0,6085	0,6110	0,6135	0,6170	0,6195	0,6200
Staphylococcus aureus	C01	6	3	irradiated group	0,0135	0,0180	0,0345	0,0710	0,1210	0,1785	0,2420	0,3180	0,3950	0,4580	0,5025	0,5320	0,5510	0,5635	0,5695	0,5750	0,5820	0,5895	0,5955	0,6015	0,6050	0,6085	0,6120	0,6155	0,6160
Staphylococcus aureus	C02	6	3	control group	0,0085	0,0160	0,0295	0,0720	0,1260	0,1845	0,2530	0,3350	0,4100	0,4720	0,5125	0,5400	0,5570	0,5695	0,5725	0,5790	0,5870	0,5955	0,6015	0,6065	0,6090	0,6125	0,6160	0,6195	0,6190
Staphylococcus aureus	C03	6	4	irradiated group	0,0105	0,0160	0,0335	0,0730	0,1250	0,1815	0,2460	0,3240	0,4000	0,4640	0,5065	0,5350	0,5520	0,5645	0,5695	0,5750	0,5820	0,5895	0,5965	0,6025	0,6050	0,6085	0,6120	0,6155	0,6150
Staphylococcus aureus	C04	6	4	control group	0,0125	0,0190	0,0355	0,0830	0,1370	0,1965	0,2630	0,3420	0,4150	0,4750	0,5125	0,5400	0,5570	0,5685	0,5735	0,5800	0,5870	0,5955	0,6025	0,6055	0,6090	0,6115	0,6150	0,6185	0,6190
Staphylococcus aureus	A01	0	5	irradiated group	0,0065	0,0100	0,0165	0,0355	0,0750	0,1240	0,1775	0,2470	0,3365	0,4260	0,5010	0,5410	0,5595	0,5660	0,5690	0,5705	0,5625	0,5665	0,5630	0,5680	0,5680	0,5715	0,5815	0,5925	0,5875
Staphylococcus aureus	A02	0	5	control group	0,0035	0,0080	0,0135	0,0325	0,0750	0,1250	0,1805	0,2500	0,3375	0,4260	0,5020	0,5430	0,5605	0,5700	0,5760	0,5765	0,5715	0,5765	0,5780	0,5760	0,5790	0,5785	0,5885	0,5955	0,6025
Staphylococcus aureus	A03	0	6	irradiated group	-0,0005	0,0040	0,0115	0,0315	0,0750	0,1270	0,1825	0,2500	0,3355	0,4240	0,4990	0,5380	0,5555	0,5630	0,5660	0,5685	0,5655	0,5715	0,5740	0,5730	0,5760	0,5785	0,5895	0,5985	0,6015
Staphylococcus aureus	A04	0	6	control group	-0,0005	0,0050	0,0145	0,0365	0,0830	0,1360	0,1925	0,2600	0,3445	0,4310	0,5040	0,5410	0,5555	0,5620	0,5660	0,5645	0,5655	0,5705	0,5750	0,5730	0,5790	0,5835	0,5925	0,6015	0,6015
Staphylococcus aureus	C01	0	7	irradiated group	0,0045	0,0080	0,0165	0,0355	0,0770	0,1270	0,1815	0,2500	0,3395	0,4260	0,4990	0,5410	0,5595	0,5680	0,5740	0,5755	0,5715	0,5755	0,5690	0,5720	0,5710	0,5705	0,5785	0,5875	0,5935
Staphylococcus aureus	C02	0	7	control group	0,0005	0,0060	0,0125	0,0335	0,0770	0,1250	0,1795	0,2480	0,3355	0,4240	0,5000	0,5400	0,5585	0,5660	0,5740	0,5755	0,5725	0,5775	0,5790	0,5780	0,5800	0,5835	0,5915	0,5995	0,6025
Staphylococcus aureus	C03	0	8	irradiated group	-0,0005	0,0040	0,0125	0,0355	0,0790	0,1280	0,1835	0,2500	0,3375	0,4270	0,5010	0,5390	0,5565	0,5640	0,5680	0,5725	0,5725	0,5775	0,5780	0,5790	0,5840	0,5865	0,5975	0,6045	0,6075
Staphylococcus aureus	C04	0	8	control group	0,0025	0,0090	0,0175	0,0395	0,0860	0,1380	0,1935	0,2620	0,3485	0,4350	0,5080	0,5440	0,5605	0,5650	0,5690	0,5715	0,5725	0,5765	0,5800	0,5830	0,5860	0,5905	0,5995	0,6075	0,6075
Staphylococcus aureus	A01	1	5	irradiated group	0,0125	0,0170	0,0285	0,0540	0,0960	0,1510	0,2160	0,2925	0,3825	0,4570	0,5145	0,5515	0,5700	0,5820	0,5865	0,5870	0,5910	0,5920	0,5965	0,6015	0,6070	0,6125	0,6200	0,6210	0,6260
Staphylococcus aureus	A02	1	5	control group	0,0075	0,0120	0,0205	0,0500	0,1050	0,1690	0,2430	0,3275	0,4145	0,4940	0,5445	0,5745	0,5910	0,6020	0,6045	0,6080	0,6130	0,6160	0,6215	0,6265	0,6320	0,6335	0,6380	0,6400	0,6430
Staphylococcus aureus	A03	1	6	irradiated group	0,0055	0,0130	0,0245	0,0510	0,0980	0,1530	0,2170	0,2905	0,3855	0,4620	0,5215	0,5575	0,5750	0,5860	0,5905	0,5920	0,5950	0,5970	0,6005	0,6055	0,6110	0,6165	0,6220	0,6260	0,6300
Staphylococcus aureus	A04	1	6	control group	0,0055	0,0130	0,0305	0,0740	0,1390	0,2030	0,2820	0,3715	0,4525	0,5200	0,5595	0,5825	0,5950	0,6030	0,6045	0,6080	0,6120	0,6150	0,6205	0,6265	0,6310	0,6325	0,6360	0,6390	0,6420
Staphylococcus aureus	C01	1	7	irradiated group	0,0105	0,0160	0,0295	0,0610	0,1090	0,1670	0,2360	0,3155	0,4025	0,4770	0,5285	0,5615	0,5790	0,5900	0,5945	0,5960	0,5990	0,6020	0,6065	0,6115	0,6170	0,6205	0,6260	0,6280	0,6320
Staphylococcus aureus	C02	1	7	control group	0,0015	0,0090	0,0205	0,0530	0,1100	0,1750	0,2510	0,3395	0,4305	0,5040	0,5495	0,5775	0,5930	0,6030	0,6055	0,6090	0,6140	0,6170	0,6225	0,6275	0,6330	0,6355	0,6380	0,6410	0,6440
Staphylococcus aureus	C03	1	8	irradiated group	0,0035	0,0130	0,0265	0,0590	0,1130	0,1740	0,2440	0,3285	0,4215	0,4930	0,5405	0,5695	0,5850	0,5940	0,5985	0,6000	0,6040	0,6070	0,6115	0,6175	0,6240	0,6285	0,6320	0,6350	0,6380
Staphylococcus aureus	C04	1	8	control group	0,0035	0,0090	0,0255	0,0600	0,1230	0,1880	0,2600	0,3485	0,4335	0,5060	0,5505	0,5785	0,5930	0,6040	0,6065	0,6080	0,6120	0,6160	0,6205	0,6265	0,6320	0,6335	0,6370	0,6390	0,6420
Staphylococcus aureus	A01	2	5	irradiated group	0,0180	0,0230	0,0375	0,0720	0,1230	0,1820	0,2510	0,3345	0,4190	0,4885	0,5350	0,5660	0,5815	0,5915	0,5965	0,5975	0,6025	0,6060	0,6100	0,6160	0,6205	0,6225	0,6245	0,6270	0,6295
Staphylococcus aureus	A02	2	5	control group	0,0140	0,0180	0,0325	0,0740	0,1330	0,1980	0,2750	0,3665	0,4490	0,5175	0,5590	0,5850	0,5995	0,6095	0,6115	0,6145	0,6195	0,6250	0,6290	0,6330	0,6345	0,6355	0,6375	0,6390	0,6415
Staphylococcus aureus	A03	2	6	irradiated group	0,0090	0,0180	0,0325	0,0650	0,1170	0,1770	0,2380	0,3185	0,4030	0,4815	0,5310	0,5640	0,5805	0,5915	0,5985	0,5985	0,6025	0,6060	0,6100	0,6160	0,6205	0,6225	0,6255	0,6290	0,6305
Staphylococcus aureus	A04	2	6	control group	0,0100	0,0160	0,0335	0,0800	0,1420	0,2070	0,2800	0,3715	0,4530	0,5185	0,5590	0,5850	0,5975	0,6065	0,6095	0,6115	0,6165	0,6220	0,6260	0,6310	0,6325	0,6325	0,6345	0,6380	0,6395
Staphylococcus aureus	C01	2	7	irradiated group	0,0140	0,0200	0,0335	0,0680	0,1170	0,1760	0,2420	0,3225	0,4070	0,4815	0,5320	0,5670	0,5845	0,5945	0,6005	0,6025	0,6065	0,6090	0,6120	0,6190	0,6225	0,6245	0,6275	0,6300	0,6325
Staphylococcus aureus	C02	2	7	control group	0,0090	0,0150	0,0325	0,0760	0,1340	0,1990	0,2760	0,3685	0,4480	0,5155	0,5580	0,5850	0,5995	0,6085	0,6115	0,6145	0,6195	0,6240	0,6280	0,6330	0,6335	0,6345	0,6375	0,6390	0,6405
Staphylococcus aureus	C03	2	8	irradiated group	0,0110	0,0160	0,0325	0,0720	0,1280	0,1910	0,2630	0,3535	0,4340	0,5055	0,5490	0,5780	0,5915	0,6015	0,6045	0,6065	0,6105	0,6150	0,6180	0,6260	0,6285	0,6295	0,6315	0,6340	0,6365
Staphylococcus aureus	C04	2	8	control group	0,0080	0,0160	0,0355	0,0830	0,1450	0,2100	0,2820	0,3735	0,4530	0,5185	0,5600	0,5850	0,5995	0,6075	0,6115	0,6135	0,6185	0,6230	0,6270	0,6320	0,6335	0,6335	0,6355	0,6380	0,6395
Staphylococcus aureus	A01	3	5	irradiated group	0,0195	0,0230	0,0390	0,0760	0,1305	0,1905	0,2605	0,3460	0,4290	0,4975	0,5440	0,5765	0,5940	0,6055	0,6110	0,6155	0,6195	0,6250	0,6300	0,6380	0,6405	0,6440	0,6475	0,6485	0,6495
Staphylococcus aureus	A02	3	5	control group	0,0135	0,0170	0,0320	0,0730	0,1305	0,1935	0,2695	0,3620	0,4440	0,5145	0,5600	0,5885	0,6050	0,6165	0,6200	0,6265	0,6295	0,6360	0,6420	0,6470	0,6495	0,6530	0,6555	0,6555	0,6575
Staphylococcus aureus	A03	3	6	irradiated group	0,0095	0,0160	0,0330	0,0700	0,1255	0,1855	0,2535	0,3390	0,4210	0,4965	0,5450	0,5775	0,5960	0,6065	0,6140	0,6175	0,6215	0,6270	0,6320	0,6390	0,6425	0,6470	0,6495	0,6505	0,6515
Staphylococcus aureus	A04	3	6	control group	0,0115	0,0180	0,0390	0,0870	0,1495	0,2125	0,2905	0,3830	0,4620	0,5265	0,5680	0,5925	0,6090	0,6165	0,6210	0,6265	0,6305	0,6370	0,6420	0,6460	0,6475	0,6520	0,6545	0,6555	0,6565
Staphylococcus aureus	C01	3	7	irradiated group	0,0135	0,0220	0,0370	0,0750	0,1285	0,1885	0,2575	0,3420	0,4250	0,4965	0,5460	0,5795	0,5970	0,6085	0,6160	0,6205	0,6235	0,6280	0,6340	0,6410	0,6445	0,6480	0,6505	0,6515	0,6535
Staphylococcus aureus	C02	3	7	control group	0,0095	0,0160	0,0320	0,0740	0,1315	0,1935	0,2715	0,3630	0,4450	0,5165	0,5620	0,5905	0,6080	0,6185	0,6220	0,6275	0,6315	0,6370	0,6430	0,6480	0,6505	0,6530	0,6565	0,6575	0,6575
Staphylococcus aureus	C03	3	8	irradiated group	0,0075	0,0170	0,0370	0,0770	0,1335	0,1955	0,2685	0,3550	0,4370	0,5085	0,5550	0,5845	0,6020	0,6125	0,6180	0,6225	0,6255	0,6310	0,6360	0,6440	0,6455	0,6500	0,6525	0,6535	0,6555
Staphylococcus aureus	C04	3	8	control group	0,0085	0,0180	0,0380	0,0850	0,1455	0,2065	0,2815	0,3740	0,4530	0,5195	0,5640	0,5905	0,6070	0,6165	0,6210	0,6265	0,6295	0,6360	0,6410	0,6460	0,6485	0,6520	0,6535	0,6555	0,6565
Staphylococcus aureus	A01	4	5	irradiated group	0,0170	0,0195	0,0365	0,0740	0,1270	0,1885	0,2580	0,3435	0,4240	0,4890	0,5385	0,5690	0,5870	0,5970	0,6010	0,6040	0,6085	0,6145	0,6185	0,6240	0,6265	0,6290	0,6325	0,6345	0,6355
Staphylococcus aureus	A02	4	5	control group	0,0130	0,0155	0,0305	0,0720	0,1300	0,1965	0,2710	0,3645	0,4460	0,5160	0,5605	0,5880	0,6040	0,6130	0,6150	0,6190	0,6235	0,6295	0,6335	0,6370	0,6395	0,6400	0,6435	0,6455	0,6465
Staphylococcus aureus	A03	4	6	irradiated group	0,0090	0,0145	0,0315	0,0670	0,1210	0,1815	0,2450	0,3265	0,4080	0,4820	0,5315	0,5660	0,5840	0,5960	0,6030	0,6050	0,6085	0,6135	0,6185	0,6240	0,6275	0,6300	0,6335	0,6345	0,6365
Staphylococcus aureus	A04	4	6	control group	0,0090	0,0155	0,0335	0,0770	0,1390	0,2045	0,2780	0,3695	0,4490	0,5180	0,5605	0,5880	0,6030	0,6120	0,6140	0,6170	0,6215	0,6265	0,6315	0,6350	0,6365	0,6380	0,6415	0,6425	0,6445
Staphylococcus aureus	C01	4	7	irradiated group	0,0160	0,0195	0,0365	0,0680	0,1190	0,1765	0,2410	0,3195	0,4030	0,4750	0,5275	0,5630	0,5850	0,5960	0,6030	0,6060	0,6085	0,6135	0,6165	0,6230	0,6275	0,6290	0,6325	0,6345	0,6355
Staphylococcus aureus	C02	4	7	control group	0,0120	0,0185	0,0345	0,0800	0,1370	0,2035	0,2800	0,3725	0,4520	0,5170	0,5625	0,5890	0,6060	0,6140	0,6160	0,6200	0,6235	0,6295	0,6335	0,6370	0,6385	0,6400	0,6435	0,6445	0,6455
Staphylococcus aureus	C03	4	8	irradiated group	0,0100	0,0165	0,0365	0,0740	0,1290	0,1935	0,2630	0,3485	0,4290	0,5010	0,5455	0,5760	0,5940	0,6040	0,6080	0,6100	0,6135	0,6195	0,6235	0,6290	0,6315	0,6340	0,6365	0,6385	0,6395
Staphylococcus aureus	C04	4	8	control group	0,0160	0,0205	0,0425	0,0910	0,1520	0,2175	0,2920	0,3845	0,4600	0,5250	0,5665	0,5910	0,6070	0,6140	0,6160	0,6200	0,6245	0,6285	0,6335	0,6360	0,6385	0,6410	0,6435	0,6445	0,6455
Staphylococcus aureus	A01	5	5	irradiated group	0,0155	0,0210	0,0360	0,0755	0,1295	0,1890	0,2680	0,3535	0,4330	0,5005	0,5460	0,5770	0,5960	0,6075	0,6110	0,6165	0,6230	0,6280	0,6345	0,6400	0,6425	0,6440	0,6485	0,6505	0,6520
Staphylococcus aureus	A02	5	5	control group	0,0145	0,0190	0,0350	0,0765	0,1355	0,1990	0,2790	0,3705	0,4520	0,5225	0,5660	0,5940	0,6120	0,6225	0,6250	0,6305	0,6370	0,6420	0,6495	0,6520	0,6535	0,6540	0,6585	0,6595	0,6610
Staphylococcus aureus	A03	5	6	irradiated group	0,0085	0,0170	0,0350	0,0775	0,1325	0,1940	0,2660	0,3555	0,4360	0,5075	0,5520	0,5810	0,6000	0,6095	0,6150	0,6195	0,6260	0,6320	0,6385	0,6430	0,6465	0,6470	0,6505	0,6525	0,6540
Staphylococcus aureus	A04	5	6	control group	0,0095	0,0180	0,0360	0,0815	0,1435	0,2050	0,2820	0,3735	0,4550	0,5225	0,5660	0,5930	0,6110	0,6195	0,6220	0,6275	0,6340	0,6390	0,6455	0,6480	0,6515	0,6520	0,6555	0,6565	0,6590
Staphylococcus aureus	C01	5	7	irradiated group	0,0105	0,0180	0,0360	0,0745	0,1295	0,1900	0,2660	0,3505	0,4320	0,4995	0,5470	0,5790	0,5990	0,6095	0,6150	0,6195	0,6260	0,6300	0,6365	0,6420	0,6455	0,6460	0,6505	0,6515	0,6540
Staphylococcus aureus	C02	5	7	control group	0,0095	0,0170	0,0330	0,0775	0,1355	0,1990	0,2800	0,3735	0,4540	0,5205	0,5660	0,5930	0,6110	0,6205	0,6230	0,6285	0,6350	0,6410	0,6475	0,6500	0,6525	0,6530	0,6565	0,6575	0,6600
Staphylococcus aureus	C03	5	8	irradiated group	0,0045	0,0150	0,0330	0,0755	0,1325	0,1940	0,2680	0,3555	0,4370	0,5075	0,5530	0,5830	0,6020	0,6125	0,6160	0,6205	0,6260	0,6320	0,6385	0,6430	0,6465	0,6470	0,6515	0,6525	0,6540
Staphylococcus aureus	C04	5	8	control group	0,0095	0,0200	0,0400	0,0875	0,1515	0,2130	0,2910	0,3855	0,4620	0,5275	0,5700	0,5960	0,6130	0,6215	0,6250	0,6295	0,6360	0,6410	0,6475	0,6500	0,6525	0,6530	0,6565	0,6575	0,6600
Staphylococcus aureus	A01	6	5	irradiated group	0,0200	0,0225	0,0375	0,0755	0,1290	0,1875	0,2555	0,3425	0,4250	0,4925	0,5415	0,5700	0,5925	0,6030	0,6075	0,6110	0,6140	0,6195	0,6240	0,6305	0,6340	0,6360	0,6390	0,6425	0,6440
Staphylococcus aureus	A02	6	5	control group	0,0140	0,0175	0,0305	0,0735	0,1310	0,1945	0,2695	0,3625	0,4460	0,5175	0,5615	0,5880	0,6075	0,6160	0,6195	0,6250	0,6280	0,6335	0,6370	0,6415	0,6440	0,6450	0,6490	0,6515	0,6520
Staphylococcus aureus	A03	6	6	irradiated group	0,0100	0,0155	0,0285	0,0675	0,1210	0,1815	0,2485	0,3315	0,4160	0,4895	0,5375	0,5690	0,5905	0,6020	0,6085	0,6120	0,6140	0,6195	0,6240	0,6305	0,6340	0,6360	0,6400	0,6425	0,6440
Staphylococcus aureus	A04	6	6	control group	0,0120	0,0165	0,0325	0,0825	0,1440	0,2075	0,2845	0,3785	0,4580	0,5245	0,5665	0,5910	0,6085	0,6170	0,6195	0,6240	0,6270	0,6325	0,6370	0,6405	0,6430	0,6450	0,6480	0,6505	0,6510
Staphylococcus aureus	C01	6	7	irradiated group	0,0140	0,0185	0,0325	0,0705	0,1220	0,1805	0,2475	0,3315	0,4160	0,4875	0,5375	0,5700	0,5925	0,6030	0,6095	0,6140	0,6160	0,6205	0,6240	0,6305	0,6350	0,6370	0,6400	0,6435	0,6450
Staphylococcus aureus	C02	6	7	control group	0,0110	0,0165	0,0295	0,0745	0,1330	0,1965	0,2725	0,3665	0,4500	0,5185	0,5635	0,5910	0,6105	0,6190	0,6215	0,6260	0,6290	0,6345	0,6390	0,6425	0,6460	0,6470	0,6500	0,6525	0,6530
Staphylococcus aureus	C03	6	8	irradiated group	0,0080	0,0155	0,0295	0,0735	0,1290	0,1915	0,2625	0,3505	0,4330	0,5035	0,5495	0,5780	0,5975	0,6070	0,6115	0,6160	0,6190	0,6235	0,6280	0,6345	0,6380	0,6400	0,6420	0,6455	0,6470
Staphylococcus aureus	C04	6	8	control group	0,0100	0,0155	0,0305	0,0805	0,1420	0,2055	0,2815	0,3735	0,4550	0,5215	0,5645	0,5910	0,6095	0,6160	0,6205	0,6250	0,6280	0,6325	0,6370	0,6415	0,6440	0,6460	0,6480	0,6505	0,6520
