# Supplementary material for: Antimicrobial effects of repeated 405 nm low-level-laser photobiomodulation: an In-vitro study on surgical wound pathogens
Source: Lasers Med Sci. 2026 May 27;41(1):103. doi: 10.1007/s10103-026-04897-2 (PMC13212771; doi:10.1007/s10103-026-04897-2)
Supplement: Supplementary file 2 — Supplementary Material 2 Supplementary Data S2: Threshold data for all strains. [file 10103_2026_4897_MOESM2_ESM.pdf]

Strain	Stage	Threshold	Control median [Q1-Q3], h)	Irradiated median [Q1-Q3], (h)	p_corr
MRSA	Stage_1	0.1	2.80 [2.70–2.85]	3.16 [3.07–3.25]	0.005 **
		0.2	3.79 [3.71–3.85]	4.23 [4.12–4.31]	0.004 **
		0.3	4.59 [4.50–4.64]	5.03 [4.88–5.11]	0.003 **
		0.4	5.34 [5.28–5.40]	5.82 [5.72–5.89]	0.002 **
		0.5	6.33 [6.28–6.37]	6.85 [6.80–6.98]	0.001 **
	Stage_2	0.1	2.77 [2.73–2.84]	3.07 [2.98–3.12]	0.005 **
		0.2	3.77 [3.73–3.84]	4.13 [4.03–4.15]	0.004 **
		0.3	4.56 [4.51–4.63]	4.90 [4.81–4.96]	0.003 **
		0.4	5.32 [5.28–5.39]	5.71 [5.62–5.75]	0.002 **
		0.5	6.34 [6.26–6.39]	6.74 [6.70–6.82]	0.001 **
	Stage_3	0.1	2.78 [2.71–2.83]	2.98 [2.94–3.02]	0.005 **
		0.2	3.80 [3.72–3.85]	4.03 [3.99–4.08]	0.004 **
		0.3	4.59 [4.51–4.62]	4.81 [4.77–4.84]	0.003 **
		0.4	5.36 [5.30–5.39]	5.59 [5.54–5.61]	0.002 **
		0.5	6.36 [6.34–6.38]	6.53 [6.46–6.66]	0.001 **
	Stage_4	0.1	2.79 [2.72–2.84]	3.04 [3.00–3.07]	0.005 **
		0.2	3.81 [3.73–3.86]	4.10 [4.05–4.13]	0.004 **
		0.3	4.61 [4.52–4.66]	4.87 [4.85–4.89]	0.003 **
		0.4	5.37 [5.28–5.42]	5.65 [5.63–5.67]	0.002 **
		0.5	6.38 [6.32–6.44]	6.61 [6.54–6.69]	0.001 **
	Stage_5	0.1	2.69 [2.58–2.81]	2.90 [2.83–3.12]	0.004 **
		0.2	3.68 [3.55–3.80]	3.98 [3.90–4.19]	0.003 **
		0.3	4.49 [4.36–4.58]	4.76 [4.71–4.96]	0.005 **
		0.4	5.23 [5.10–5.36]	5.51 [5.47–5.72]	0.002 **
		0.5	6.24 [6.09–6.37]	6.42 [6.37–6.63]	0.010 *
	Stage_6	0.1	2.73 [2.64–2.78]	2.85 [2.82–2.92]	0.005 **
		0.2	3.71 [3.61–3.79]	3.93 [3.86–4.00]	0.004 **
		0.3	4.51 [4.40–4.56]	4.71 [4.64–4.76]	0.003 **
		0.4	5.24 [5.10–5.35]	5.42 [5.32–5.54]	0.100 n.s.
		0.5	6.18 [5.92–6.44]	6.25 [6.05–6.47]	0.279 n.s.
Escherichia coli	Stage_1	0.1	2.18 [2.13–2.21]	2.16 [2.13–2.18]	1.000 n.s.
		0.2	2.77 [2.73–2.85]	2.78 [2.74–2.82]	1.000 n.s.
		0.3	3.32 [3.27–3.42]	3.33 [3.25–3.41]	1.000 n.s.
		0.4	3.87 [3.80–3.97]	3.88 [3.79–3.95]	0.959 n.s.
		0.5	4.72 [4.60–4.78]	4.73 [4.57–4.88]	1.000 n.s.
	Stage_2	0.1	2.14 [2.10–2.19]	2.10 [2.07–2.15]	0.420 n.s.
		0.2	2.77 [2.75–2.81]	2.73 [2.71–2.74]	0.105 n.s.
		0.3	3.30 [3.25–3.40]	3.28 [3.25–3.31]	1.000 n.s.
		0.4	3.84 [3.80–3.92]	3.83 [3.80–3.85]	0.884 n.s.
		0.5	4.73 [4.72–4.85]	4.78 [4.69–4.79]	1.000 n.s.
	Stage_3	0.1	2.13 [2.09–2.14]	2.08 [2.06–2.15]	0.442 n.s.
		0.2	2.71 [2.66–2.75]	2.73 [2.70–2.76]	1.000 n.s.
		0.3	3.24 [3.18–3.28]	3.26 [3.24–3.28]	1.000 n.s.
		0.4	3.76 [3.72–3.79]	3.79 [3.77–3.84]	0.415 n.s.
		0.5	4.47 [4.40–4.55]	4.60 [4.41–4.80]	0.764 n.s.
	Stage_4	0.1	2.02 [1.99–2.08]	2.12 [2.07–2.14]	0.030 *
		0.2	2.62 [2.53–2.71]	2.73 [2.71–2.74]	0.030 *
		0.3	3.14 [3.03–3.22]	3.27 [3.22–3.28]	0.020 *
		0.4	3.64 [3.53–3.70]	3.79 [3.78–3.88]	0.005 **
		0.5	4.37 [4.30–4.46]	4.68 [4.40–5.21]	0.065 n.s.
	Stage_5	0.1	1.89 [1.81–1.93]	2.06 [2.04–2.08]	0.005 **
		0.2	2.47 [2.44–2.57]	2.65 [2.61–2.69]	0.002 **
		0.3	2.99 [2.92–3.12]	3.18 [3.16–3.21]	0.004 **
		0.4	3.49 [3.44–3.60]	3.72 [3.68–3.74]	0.003 **
		0.5	4.24 [4.21–4.30]	4.44 [4.36–4.63]	0.002 **
	Stage_6	0.1	1.86 [1.83–1.98]	2.03 [1.96–2.07]	0.028 *
		0.2	2.47 [2.44–2.57]	2.64 [2.57–2.69]	0.021 *
		0.3	2.97 [2.93–3.15]	3.20 [3.11–3.28]	0.020 *
		0.4	3.46 [3.43–3.67]	3.74 [3.63–3.81]	0.010 *
		0.5	4.31 [4.23–4.38]	4.49 [4.42–4.56]	0.005 **
Pseudomonas aeruginosa	Stage_1	0.1	2.05 [1.93–2.25]	3.13 [2.27–3.37]	0.007 **
		0.2	3.34 [3.14–3.44]	4.56 [3.77–4.77]	0.005 **
		0.3	4.19 [3.94–4.24]	5.47 [4.70–5.71]	0.004 **
		0.4	5.09 [4.82–5.14]	6.45 [5.60–6.73]	0.003 **
		0.5	5.91 [5.69–5.94]	7.17 [6.58–7.44]	0.002 **
	Stage_2	0.1	1.85 [1.70–2.03]	2.41 [2.04–2.65]	0.021 *
		0.2	3.06 [2.89–3.23]	3.80 [3.38–4.14]	0.014 *
		0.3	3.87 [3.66–4.02]	4.67 [4.24–5.05]	0.025 *
		0.4	4.74 [4.50–4.89]	5.58 [5.08–6.09]	0.020 *
		0.5	5.64 [5.44–5.78]	6.55 [6.01–7.03]	0.015 *
	Stage_3	0.1	2.03 [1.97–2.26]	2.71 [1.94–3.53]	0.390 n.s.
		0.2	3.30 [3.18–3.64]	4.12 [3.37–4.90]	0.140 n.s.
		0.3	4.20 [3.97–4.64]	5.05 [4.25–5.93]	0.152 n.s.
		0.4	5.35 [4.88–6.00]	6.11 [5.11–7.01]	0.260 n.s.
		0.5	6.33 [5.72–7.05]	6.92 [6.01–7.73]	0.161 n.s.
	Stage_4	0.1	2.58 [1.91–3.16]	3.15 [2.26–4.60]	0.130 n.s.
		0.2	3.87 [3.16–4.48]	4.78 [3.78–6.04]	0.190 n.s.
		0.3	4.76 [3.99–5.52]	5.89 [4.77–7.27]	0.152 n.s.
		0.4	5.91 [5.05–6.66]	7.13 [5.85–8.38]	0.114 n.s.
		0.5	7.06 [5.93–7.46]	8.01 [6.65–9.16]	0.166 n.s.

	Stage_5	0.1	2.55 [2.32–2.92]	3.33 [2.33–4.44]	0.210 n.s.
		0.2	3.73 [3.57–4.18]	4.82 [3.73–5.94]	0.105 n.s.
		0.3	4.63 [4.46–5.17]	5.94 [4.77–7.27]	0.150 n.s.
		0.4	5.66 [5.40–6.21]	7.16 [5.89–7.91]	0.140 n.s.
		0.5	6.41 [6.18–6.95]	7.96 [6.64–8.72]	0.112 n.s.
	Stage_6	0.1	2.50 [2.37–2.66]	3.03 [2.19–5.24]	0.195 n.s.
		0.2	3.62 [3.48–3.91]	4.81 [3.66–6.64]	0.130 n.s.
		0.3	4.51 [4.26–4.86]	5.70 [4.80–7.94]	0.084 n.s.
		0.4	5.50 [5.18–5.86]	6.36 [5.50–9.04]	0.063 n.s.
		0.5	6.19 [5.88–6.62]	7.24 [6.36–9.81]	0.050 n.s.
Enterococcus faecalis	Stage_1	0.1	2.03 [2.02–2.09]	1.97 [1.94–2.00]	0.008 **
		0.2	2.65 [2.64–2.71]	2.59 [2.55–2.61]	0.005 **
		0.3	3.14 [3.10–3.20]	3.08 [3.03–3.09]	0.006 **
		0.4	3.62 [3.54–3.72]	3.56 [3.47–3.58]	0.260 n.s.
		0.5	4.47 [4.14–5.28]	4.35 [4.04–4.86]	0.505 n.s.
	Stage_2	0.1	2.05 [1.99–2.10]	1.98 [1.94–2.03]	0.084 n.s.
		0.2	2.69 [2.63–2.73]	2.63 [2.58–2.66]	0.063 n.s.
		0.3	3.17 [3.10–3.23]	3.11 [3.05–3.16]	0.166 n.s.
		0.4	3.66 [3.53–3.77]	3.60 [3.48–3.70]	0.328 n.s.
		0.5	n.r.	n.r.	
	Stage_3	0.1	2.05 [2.02–2.09]	1.99 [1.96–2.00]	0.003 **
		0.2	2.69 [2.66–2.71]	2.60 [2.57–2.62]	0.004 **
		0.3	3.16 [3.14–3.21]	3.08 [3.03–3.12]	0.006 **
		0.4	3.66 [3.58–3.76]	3.58 [3.47–3.67]	0.105 n.s.
		0.5	5.31 [4.19–7.80]	n.r.	
	Stage_4	0.1	2.05 [1.99–2.10]	2.02 [1.93–2.06]	0.558 n.s.
		0.2	2.68 [2.63–2.73]	2.63 [2.58–2.68]	0.420 n.s.
		0.3	3.17 [3.11–3.24]	3.12 [3.04–3.20]	0.702 n.s.
		0.4	3.69 [3.54–3.82]	3.64 [3.47–3.80]	0.328 n.s.
		0.5	n.r.	n.r.	
	Stage_5	0.1	2.05 [2.02–2.07]	1.96 [1.95–1.97]	0.003 **
		0.2	2.68 [2.65–2.71]	2.59 [2.57–2.60]	0.004 **
		0.3	3.16 [3.12–3.21]	3.07 [3.03–3.10]	0.006 **
		0.4	3.65 [3.57–3.75]	3.55 [3.46–3.67]	0.105 n.s.
		0.5	5.61 [4.16–7.81]	n.r.	
	Stage_6	0.1	2.05 [2.02–2.08]	2.00 [1.98–2.01]	0.030 *
		0.2	2.69 [2.65–2.71]	2.62 [2.60–2.63]	0.020 *
		0.3	3.17 [3.12–3.21]	3.11 [3.06–3.13]	0.100 n.s.
		0.4	3.66 [3.56–3.75]	3.61 [3.50–3.68]	0.161 n.s.
		0.5	n.r.	n.r.	
Staphylococcus aureus	Stage_1	0.1	1.91 [1.83–1.95]	2.05 [1.93–2.18]	0.195 n.s.
		0.2	2.69 [2.60–2.73]	2.91 [2.77–3.10]	0.075 n.s.
		0.3	3.35 [3.24–3.42]	3.59 [3.44–3.81]	0.084 n.s.
		0.4	3.99 [3.81–4.12]	4.22 [4.01–4.50]	0.130 n.s.
		0.5	4.84 [4.46–5.17]	5.18 [4.75–5.63]	0.105 n.s.
	Stage_2	0.1	1.71 [1.67–1.78]	1.83 [1.79–1.85]	0.021 *
		0.2	2.53 [2.46–2.60]	2.70 [2.64–2.71]	0.005 **
		0.3	3.17 [3.11–3.26]	3.39 [3.31–3.42]	0.008 **
		0.4	3.79 [3.68–3.93]	4.04 [3.91–4.13]	0.030 *
		0.5	4.66 [4.36–5.00]	4.97 [4.64–5.31]	0.105 n.s.
	Stage_3	0.1	1.70 [1.64–1.76]	1.73 [1.70–1.75]	0.442 n.s.
		0.2	2.54 [2.47–2.59]	2.58 [2.56–2.61]	0.520 n.s.
		0.3	3.18 [3.11–3.25]	3.25 [3.23–3.27]	0.525 n.s.
		0.4	3.80 [3.68–3.92]	3.88 [3.88–3.94]	0.656 n.s.
		0.5	4.64 [4.36–4.95]	4.73 [4.53–5.01]	0.585 n.s.
	Stage_4	0.1	1.71 [1.66–1.77]	1.78 [1.74–1.80]	0.260 n.s.
		0.2	2.52 [2.47–2.62]	2.65 [2.57–2.68]	0.260 n.s.
		0.3	3.17 [3.11–3.28]	3.34 [3.23–3.38]	0.140 n.s.
		0.4	3.79 [3.68–3.94]	3.97 [3.87–4.04]	0.195 n.s.
		0.5	4.63 [4.37–4.96]	4.85 [4.63–5.04]	0.279 n.s.
	Stage_5	0.1	1.69 [1.66–1.77]	1.75 [1.72–1.81]	0.190 n.s.
		0.2	2.53 [2.47–2.63]	2.61 [2.55–2.71]	0.420 n.s.
		0.3	3.17 [3.10–3.29]	3.26 [3.19–3.41]	0.315 n.s.
		0.4	3.79 [3.66–3.96]	3.91 [3.78–4.10]	0.210 n.s.
		0.5	4.63 [4.34–4.97]	4.80 [4.46–5.20]	0.105 n.s.
	Stage_6	0.1	1.72 [1.66–1.75]	1.77 [1.74–1.80]	0.152 n.s.
		0.2	2.53 [2.47–2.60]	2.64 [2.60–2.66]	0.105 n.s.
		0.3	3.20 [3.11–3.28]	3.32 [3.27–3.37]	0.114 n.s.
		0.4	3.81 [3.67–3.93]	3.94 [3.86–4.03]	0.260 n.s.
		0.5	4.60 [4.35–4.84]	4.77 [4.58–4.96]	0.195 n.s.

Supplementary Data S2. Threshold data for all strains. Threshold = predefined OD_{600} threshold value. Data are presented as median [Q1–Q3] in hours (h), where Q1 represents the 25th percentile and Q3 the 75th percentile. p_corr = Bonferroni–Holm corrected p-values. $p < 0.05$ (*), $p < 0.01$ (**); n.s. = not significant. n.r. = threshold not reached within the measured range; therefore, no threshold time could be determined.
